# Monoterpene indole alkaloids from the aerial parts of *Ophiorrhiza brevidentata* and their immunological activities

**DOI:** 10.1007/s13659-025-00575-y

**Published:** 2026-01-11

**Authors:** Fan Xu, Zheng-Hui Li, Meng-Lin Feng, Jia-Yu Jin, Bao-Bao Shi, Ji-Kai Liu

**Affiliations:** 1 School of Pharmaceutical Sciences, South-Central Minzu University, Wuhan, 430074 People’s Republic of China; 2 International Cooperation Base for Active Substances in Traditional Chinese Medicine in Hubei Province, School of Pharmaceutical Sciences, South-Central Minzu University, Wuhan, 430074 People’s Republic of China

**Keywords:** Monoterpene indole alkaloids, ophiorrhiza brevidentata, immunological activity

## Abstract

Phytochemical study of the EtOAc extract of *Ophiorrhiza brevidentata* resulted in the discovery of 10 new monoterpene indole alkaloids (**1–10**), along with 13 known compounds (**11 − 23**). Compound **1** possess an unprecedented skeleton consisting of fused 6/5/6/7/6 polycyclic systems. The structural characterization of these compounds was achieved using Nuclear Magnetic Resonance, Mass Spectrometry and Quantum Chemical Calculations. Compounds **3–5** and **9** exhibited strong inhibition on lipopolysaccharide-induced B cell proliferation with IC_50_ values ranging from 3.6–9.1 µM with excellent selectivity indices (SI > 10).

**Graphical abstract:**

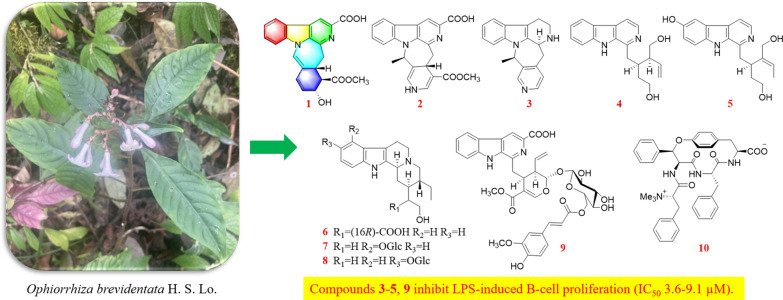

**Supplementary Information:**

The online version contains supplementary material available at 10.1007/s13659-025-00575-y.

## Introduction

The immune system is a highly complex and finely regulated network of cells, tissues, and organs that plays a crucial role in maintaining the body’s homeostasis and health. Its primary functions include identifying and eliminating foreign invaders as well as monitoring and removing abnormal or cancerous cells [1]. However, when the immune system fails to distinguish between foreign antigens and the body’s own structures, it may produce autoantibodies that mistakenly attack healthy cells. This breakdown in self-tolerance can lead to the development of autoimmune diseases [2]. It is estimated that approximately 5–8% of the global population is affected by more than 150 different types of autoimmune disorders [3, 4]. Common autoimmune diseases encompass a spectrum of disorders, such as systemic lupus erythematosus, ankylosing spondylitis, Sjögren’s syndrome, rheumatoid arthritis, and scleroderma, among others [5]. It is noteworthy that curative therapies are presently unavailable for numerous autoimmune disorders. Consequently, pharmacological intervention constitutes the mainstay of treatment [6]. However, most current immunosuppressive drugs cause severe side effects, including hepatotoxicity, nephrotoxicity, metabolic disorders, teratogenicity, and infertility [7–9]. Therefore, developing novel agents with higher efficacy and lower toxicity is urgently needed for treating autoimmune diseases.

**Fig. 1:**
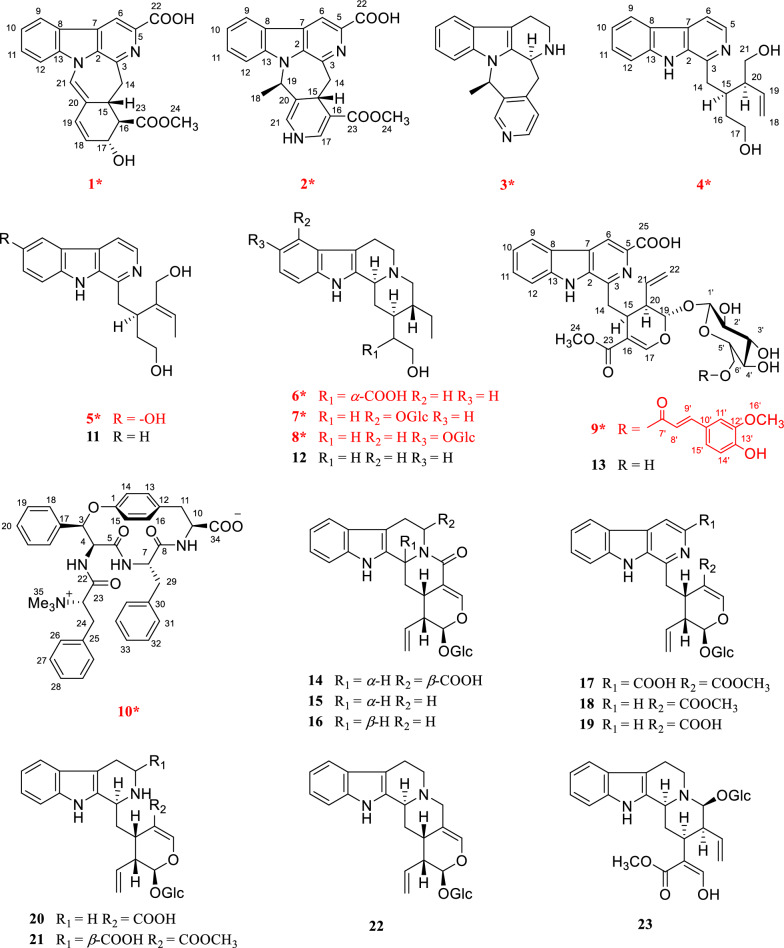
Chemical structures of compounds **1–23**

*Ophiorrhiza*, a genus within the Rubiaceae family, comprises approximately 200 species, with 70 occurring in China [10]. Members of the Rubiaceae family are known to biosynthesize a wide array of secondary metabolites, such as indole alkaloids, anthraquinones, triterpenoids, and iridoids [11]. Among these, alkaloids represent the major biologically active compounds in *Ophiorrhiza* species, contributing to their diverse pharmacological properties such as anti-inflammatory, antioxidant, analgesic, anticancer, and antiviral effects [10]. Several novel, structurally distinct alkaloids exhibiting significant biological activities have been isolated from *O. japonica* in our preliminary research [12–15]. Notably, ophiorrhines A and B feature a unique bridged carbon framework formed via an intramolecular [4 + 2] Diels–Alder reaction [15]. Ophiorrhines F and G—key biogenetic precursors to ophiorrhines A and B—are characterized by an opened C-ring structure and have demonstrated pronounced and selective inhibition of B cell proliferation [12]. Consequently, a further investigation of other *Ophiorrhiza* species was undertaken in search of immunosuppressive alkaloids. This effort resulted in the isolation of ten previously undescribed alkaloids, named ophiorbrevines A–J (**1–10**), together with 13 known analogues (**11–23**) (Fig. 1), from title species*.* Herein, the isolation, structure elucidation, and immunosuppressive activities of these compounds are reported.

## Results and discussion

**Fig. 2:**
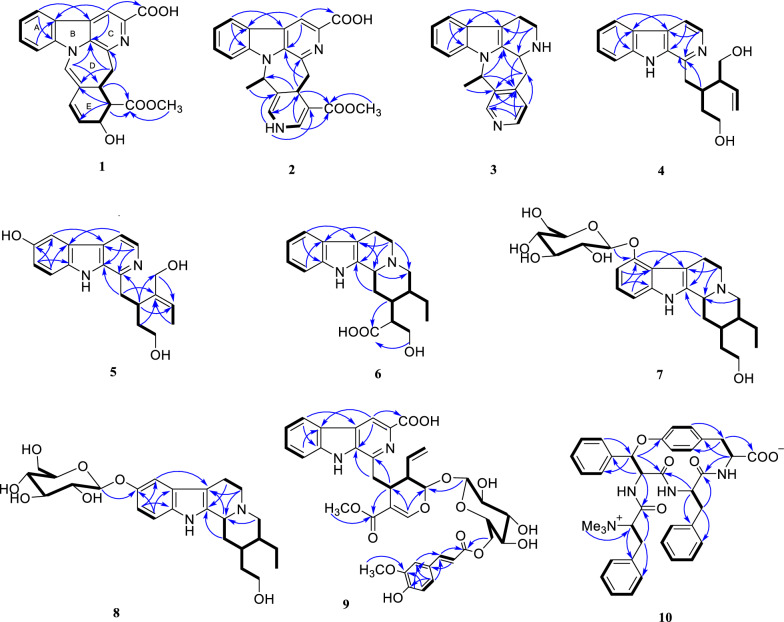
Key ^1^H-^1^H COSY and HMBC correlations for **1**–**10**

**Fig. 3:**
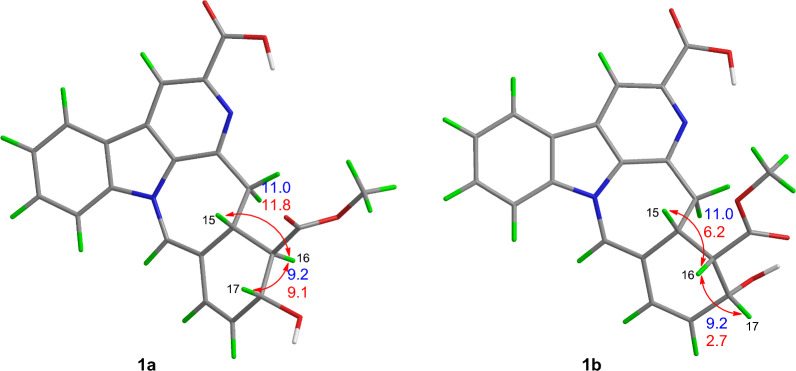
Calculated (red) and experimental (blue) spin–spin coupling constants of **1a** and **1b**

**Fig. 4:**
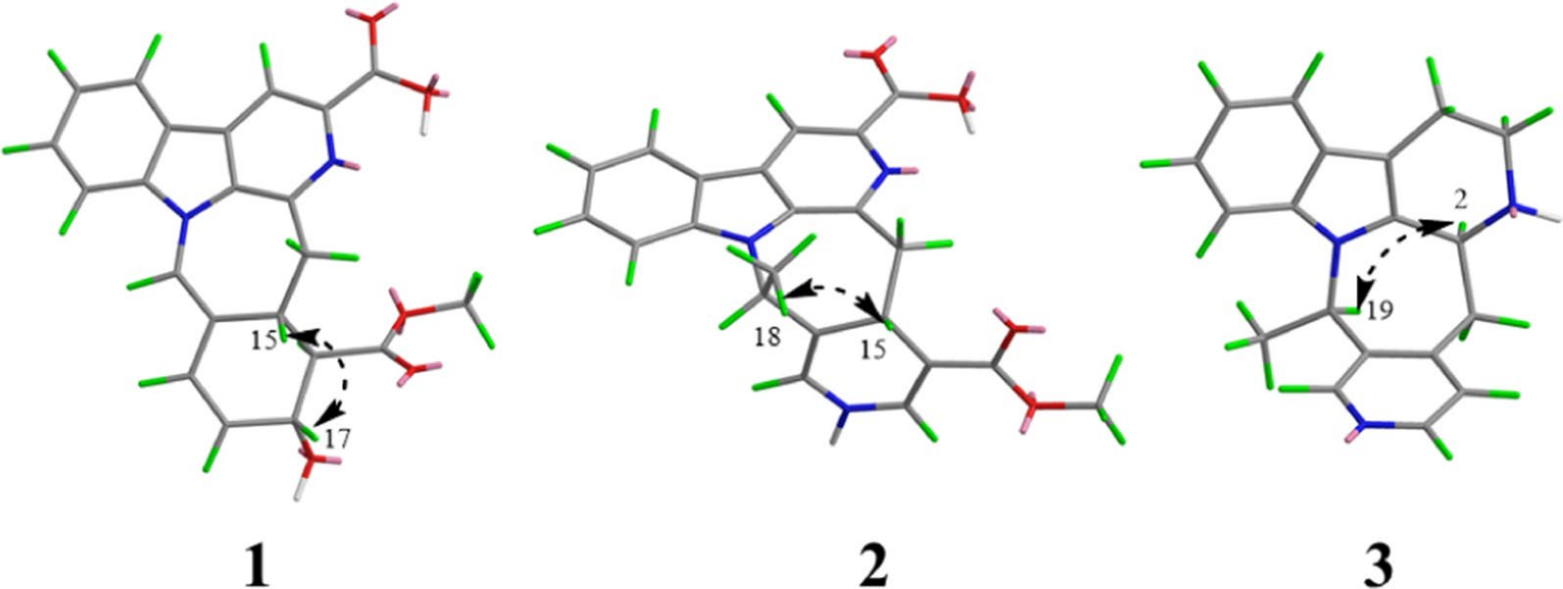
Key ROESY correlations for **1–3**

**Fig. 5:**
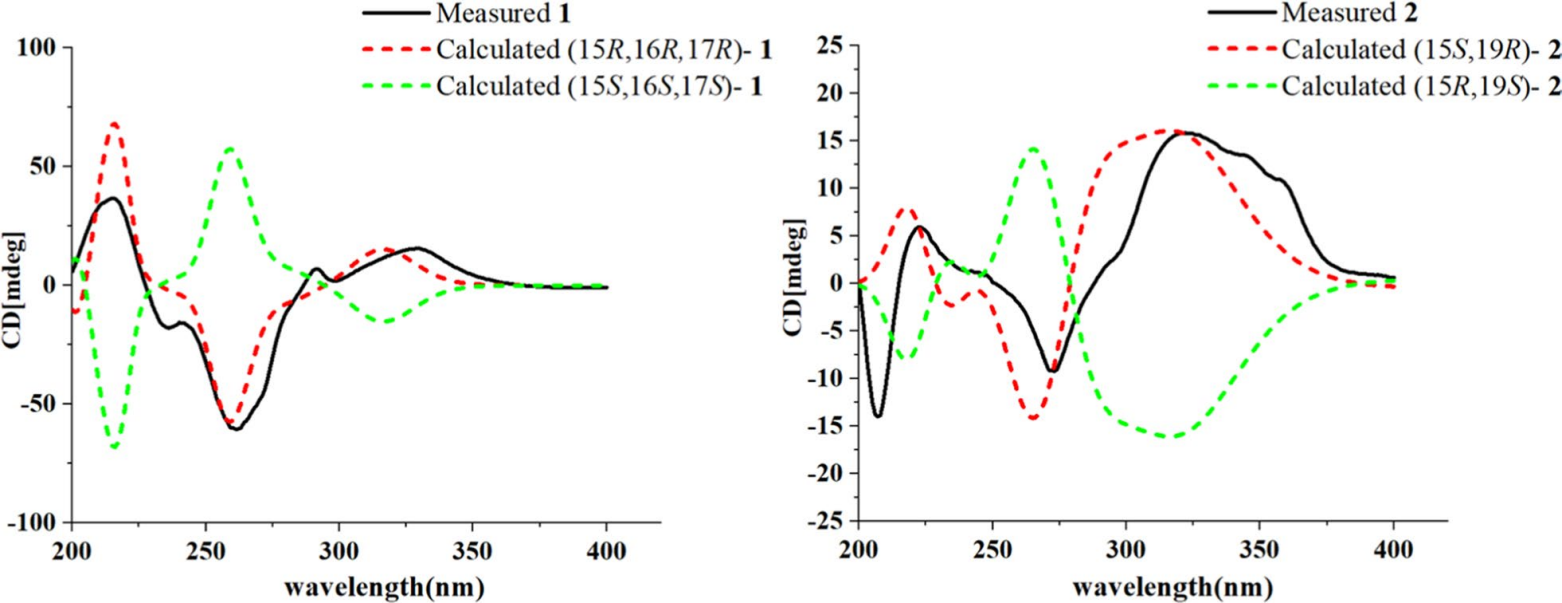
ECD calculations of **1** and **2**

Compound **1** was obtained as yellow powder, and its molecular formula was confirmed as C_22_H_18_O_5_N_2_ from ESIHRMS at *m/z* 391.12991 [M + H]^+^ (calcd. for C_22_H_19_N_2_O_5_^+^, 391.12885), with 13 degrees of unsaturation. The ^1^H NMR spectrum (Table 1) exhibited characteristic signals corresponding to a 1,2-disubstituted phenyl ring, observed at *δ*_H_ 8.54 (1H, d, *J* = 7.6 Hz), 7.58 (1H, t, *J* = 7.6 Hz), 7.86 (1H, t, *J* = 7.6 Hz), and 8.20 (1H, d,* J* = 7.6 Hz). In addition, signals for four alkene protons were detected at *δ*_H_ 7.84 (1H, m), 5.92 (1H, dd, *J* = 9.9, 2.1 Hz), 6.56 (1H, dd, *J* = 9.9, 2.1 Hz), and 8.90 (1H, s), along with a methoxy signal at *δ*_H_ 3.93 (3H, s). Compound **1** showed 22 carbon signals in its ^13^C NMR spectrum (Table 1), including one methoxy group, one methylene, eleven methines, and nine quaternary carbons. The HMBC correlation (Fig. 2) from H-9 to C-7/C-13, from H-12 to C-8, and H-6 to C-2/C-3/C-8 confirmed the presence of *β*-carboline moiety. The correlations from H-6 to the carboxyl carbon at *δ*_C_ 167.5 (C-22) suggested that a carboxyl group was located at C-5. The C_14_–C_19_ fragment (C_14_-C_15_-C_16_-C_17_-C_18_-C_19_), which was established by ^1^H–^1^H COSY, HMBC, and HSQC spectra (Fig. 2), was determined to be connected to the* β*-carboline moiety at C-3. This connection was evidenced by key HMBC correlations from H-14 to C-2/C-3 and from H-15 to C-3. The key HMBC correlations from H-12/H-16 to C-20 and from H-19 to C-15 established the linkage between C-15 and C-20. Further HMBC correlations from H-15 to C-21 and from H-21 to C-2, C-13, C-15, and C-19, along with the chemical shift of C-21 (*δ*_C_ 122.2) and C-20 (*δ*_C_ 167.5), established the presence of a seven-membered nitrogen heterocycles with a double bond between C-20 and C-21. The HMBC correlations from both H-16 and H-24 to C-23 indicated that the ester group was attached to C-16. Based on biosynthetic pathway analysis, H-15 was assigned the *β* configuration. ROESY correlations between H-15 and H-17 (Fig. 4) further indicate that these protons were cofacial. The configuration of remaining H-16 was determined by comparing the experimental coupling constants of H-15/H-16 and H-16/H-17 with computed values. The calculated values for the 16R* configuration [11.8 Hz (^3^*J*_H-15/H-16_) and 9.1 Hz (^3^*J*_H-16/H-17_), Fig. 3] showed closer agreement with the experimental data [11.0 Hz (^3^*J*_H-15/H-16_) and 9.2 Hz (^3^*J*_H-16/H-17_)] than those for the 16S* configuration [5.7 Hz (^3^*J*_H-15/H-16_) and 2.7 Hz (^3^*J*_H-16/H-17_)]. Finally, the absolute configuration of compound **1** was assigned as 15*R*,16*R*,17*R* by comparing the calculated and experimental electronic circular dichroism (ECD) spectra (Fig. 5).

**Table 1:** ^1^H and ^13^C NMR spectroscopic data of compounds **1**−**3** (DMSO and CD_3_OD)

No	**1**^a^		**2**^a^		**3**^b^	
	*δ*_C_, type	*δ*_H_, mult (*J* in Hz)	*δ*_C_, type	*δ*_H_, mult (*J* in Hz)	*δ*_C_, type	*δ*_H_, mult (*J* in Hz)
2	133.6, C		135.3, C		135.7, C	
3	143.9, C		143.8, C		48.3, CH	4.39, d (6.8)
5	137.1, C		136.9, C		49.8, CH_2_	3.16, ddd (11.4, 5.1, 2.7)
						3.06, td (10.7, 4.3)
6	115.1, CH	8.90, s	114.9, CH	8.76, s	22.5, CH_2_	2.93, tdd (10.3, 5.2, 2.1)
						2.88, m
7	129.8, C		129.7, C		108.3, C	
8	121.4, C		121.8, C		128.2, C	
9	122.2, CH	8.54, d (7.6)	122.0, CH	8.39, d (7.5)	118.8, CH	7.41, d (8.0)
10	122.2, CH	7.58, t (7.6)	120.6, CH	7.35, t (7.5)	119.9, CH	6.98, t (8.0)
11	129.1, CH	7.86, t (7.6)	128.7, CH	7.67, t (7.5)	122.2, CH	7.06, t (8.0)
12	111.2, CH	8.20, d (7.6)	111.9, CH	7.85, d (7.5)	111.9, CH	7.30, d (8.0)
13	140.1, C		140.8, C		138.4, C	
14	41.6, CH_2_	3.61, dd (15.3, 10.6)	48.1, CH_2_	3.04, m	34.9, CH_2_	3.44, dd (17.4, 4.3)
		3.44, d (15.3)		3.41, dd (16.0, 2.8)		2.83, m
15	36.5, CH	3.35 dd (11.0, 10.6)	28.5, CH	4.02, dd (11.7, 2.9)	144.8, C	
16	55.3, CH	2.79, dd (11.0, 9.2)	99.3, C		125.4, CH	7.24, d (5.2)
17	67.73, CH	4.58, d (9.2)	138.0, CH	7.42, d (5.5)	147.3, CH	8.27, d (5.2)
18	130.6, CH	5.92, dd (9.9, 2.1)	18.1, CH_3_	1.35, d (6.6)	16.7, CH_3_	1.45, d (7.0)
19	128.3, CH	6.56, dd (9.9, 2.1)	55.9, CH	5.78, q (6.5)	57.4, CH	4.41, q (7.0)
20	122.4, C		114.1, C		138.4, C	
21	122.2, CH	7.84, s	124.2, CH	6.70, d (4.3)	149.0, CH	8.37, s
22	167.5, C		167.1, C			
23	174.5, C		167.6, C			
24	52.0, CH_3_	3.93, s	50.9, CH_3_	3.68, s		
N–H				8.80, s		

^a^Measured on 600/150 MHz in DMSO-d6. ^b^Measured on 500/125 MHz in CD_3_OD

The molecular formula of compound **2**, an orange powder, was established as C_22_H_19_O_4_N_3_ from ESIHRMS data, which showed an [M + H]⁺ ion at *m/z* 390.14465 (calcd 390.14483). This formula corresponds to 15 degrees of unsaturation. The UV spectrum exhibited absorption maxima at 210, 240, and 270 nm, which were distinct from those of compound **1**. Its ^1^H NMR spectrum showed the presence of one methyl group (*δ*_H_ 1.35), one methoxy group (*δ*_H_ 3.68), and seven alkene protons (*δ*_H_ 8.76, 8.39, 7.35, 7.67, 7.85, 7.42, and 6.70) (Table 1). Its ^13^C NMR spectrum exhibited 22 distinct resonances, accounting for two methyl groups, one methylene, nine methines, and ten quaternary carbons (including two carbonyls). Careful analysis of the NMR data (Table 1) revealed that **2** was very similar to Mappianine B [16]. The key structural difference between the two compounds is the lack of an ethyl group at N-25 and the addition of a carboxyl group at C-5 in compound **2**. This assignment was supported by HMBC correlations from H-N_25_ to C-16 (*δ*_C_ 99.3)/C-20 (*δ*_C_ 114.1) and from H-6 to C-22 (*δ*_C_ 167.1) in spectrum. The combined analysis of the ROESY spectrum (Fig. 4), which showed correlations from H-18 to H-15, and the calculated ECD spectra (Fig. 5) confirmed the absolute configuration of **2** as 15*S*,19*R*.

Compound **3** was obtained as yellow powder, and its molecular formula was determined to be C_19_H_19_N_3_ from ESIHRMS, exhibiting a protonated ion peak at *m/z* 290.16501 [M + H]^+^ (calcd for C_19_H_20_N_3_^+^, 290.16517), consistent with 12 degrees of unsaturation. The ^1^H NMR spectrum exhibited signals characteristic of an indole moiety, including resonances at *δ*_H_ 7.41 (1H, d, *J* = 8.0 Hz), 6.98 (1H, t, *J* = 8.0 Hz), 7.06 (1H, t, *J* = 8.0 Hz), and 7.30 (1H, d, *J* = 8.0 Hz), along with a methyl signal observed at *δ*_H_ 1.45 (3H, d, *J* = 7.0 Hz). Compound **3** was characterized as a yellow powder with the molecular formula C_19_H_19_N_3_ (12 degrees of unsaturation), as evidenced by ESIHRMS (*m/z* 290.16501 [M + H]⁺; calcd 290.16517). The ^1^H NMR data supported the presence of an indole ring system [*δ*_H_ 7.41 (1H, d, *J* = 8.0 Hz), 6.98 (1H, t, *J* = 8.0 Hz), 7.06 (1H, t, *J* = 8.0 Hz), 7.30 (1H, d, *J* = 8.0 Hz)], along with a methyl doublet at *δ*_H_ 1.45 (3H, d, *J* = 7.0 Hz). Analysis of the ^13^C NMR and DEPT spectra indicated the presence of six quaternary carbons (*δ*_C_ 135.7, 108.3, 128.2, 138.4, 144.8, 138.4), nine methines (*δ*_C_ 48.3, 118.8, 119.9, 122.2, 111.9, 125.4, 147.3, 57.4, 149.0), three methylenes (*δ*_C_ 49.8, 22.5, 34.9), and one methyl group (*δ*_C_ 16.7). The NMR data for compound **3** closely matched those of 3,14-dihydrodecussine [17], with the key difference being the absence of an N_4_-methyl resonance. This observation is consistent with the ESIHRMS data, which indicated a molecular weight 14 units lower for compound **3**, supporting the lack of the N-methyl group. The ROESY correlation observed between H-19 and H-3confirmed that these protons are co-facial (Fig. 4). This observation, combined with the biosynthesis pathway, allows the absolute configuration to be inferred to be 3*S*,19*R.*

Compound **4** was characterized as a yellow powder with the molecular formula C_19_H_22_N_2_O_2_ (10 degrees of unsaturation), as determined from ESI-HRMS (*m/z* 311.17517 [M + H]⁺; calcd 311.17540). The ^1^H NMR data were consistent with the presence of a *β*-carboline moiety, evident from a characteristic set of six aromatic proton signals [*δ*_H_ 8.22 (1H, d, *J* = 5.4 Hz, H-5), 7.95 (1H, d, *J* = 5.4 Hz, H-6), 8.16 (1H, d, *J* = 8.0, H-9), 7.25 (1H, ddd, *J* = 8.0, 6.9, 1.1 Hz, H-10), 7.55 (1H, ddd, *J* = 8.0, 6.9, 1.1 Hz, H-11), 7.58 (1H, d, *J* = 8.0,, H-12)]. Additionally, signals for a terminal double bond were observed at δ_H_ 5.20 (1H, dd, *J* = 10.4 2.0 Hz, H-18a), 5.10 (1H, ddd, *J* = 17.2, 2.0, 0.9 Hz, H-18b), 5.85 (1H, ddd, *J* = 17.2, 10.4, 8.9 Hz, H-19). Analysis of the ^13^C NMR and DEPT data identified 19 carbon resonances, comprising five methylenes, nine methines, and five quaternary carbons (Table 2). The ^1^H and ^13^C NMR data of **4** were structurally similar to those of the known compound 10-hydroxy-iso-deppeaninol [18], except for the absence of hydroxyl substitution at C-10 in compound **4**. This presumption was confirmed by the ^1^H-^1^H COSY correlations of H-9/H-10/H-11/H-12. Considering the biogenetic relationship between **4** and 10-hydroxy-iso-deppeaninol, their stereochemical structures were predicted to be identical. Consequently, the absolute configuration of **4** was assigned as 15*R*,20*S.*

**Table 2:** ^1^H and ^13^C NMR spectroscopic data of compounds **4–5** (CD_3_OD)

No	**4**^a^		**5**^b^	
	*δ*_C_, type	*δ*_H_, mult (*J* in Hz)	*δ*_C_, type	*δ*_H_, mult (*J* in Hz)
2	136.5, C		137.3, C	
3	146.3, C		145.8, C	
5	138, CH	8.22, d (5.4)	136.7, CH	8.09, d (5.4)
6	114.2, CH	7.95, d (5.4)	114.2, CH	7.80, d (5.4)
7	130.2, C		130.0, C	
8	122.7, C		123.2, C	
9	122.6, CH	8.16, d (8.0)	106.7, CH	7.46, d (2.4)
10	120.8, CH	7.25, t (8.0)	152.3, C	
11	129.5, CH	7.55, t (8.0)	119.6, CH	7.09, dd (8.8, 2.4)
12	112.9, CH	7.58, d (8.0)	113.5, CH	7.39, d (8.8)
13	142.6, C		137.1, C	
14	37.5, CH_2_	3.18, m	39.3, CH_2_	3.33, m
15	36.4, CH	2.54, tt (7.6, 3.8)	36.7, CH	3.38, m
16	34.1, CH_2_	1.81,m	36.9, CH_2_	1.96, m
		1.51, m		1.87, m
17	61.5, CH_2_	3.46, m	61.2, CH_2_	3.60, m
				3.50, m
18	118.6, CH_2_	5.20, dd, (10.4, 2.0)	12.8, CH_3_	1.00, d (6.9)
		5.10, ddd (17.2, 2.0, 0.9)		
19	138.3, CH	5.85, ddd (17.2, 10.4, 8.9)	126.6, CH	5.37, q (6.9)
20	50.9, CH	2.29, m	140.0, C	
21	64.5, CH_2_	3.62, d (6.8)	65.5, CH_2_	4.26, m
				4.04, d (12.3)

^a^Measured on 600/150 MHz. ^b^Measured on 500/125 MHz

Compound **5** was characterized as a yellow powder with the molecular formula C_19_H_22_N_2_O_3_ (10 degrees of unsaturation), as determined by ESI-HRMS (*m/z* 327.17010 [M + H]⁺; calcd 327.17032). The ^1^H NMR spectrum supported the *β*-carboline structure, showing key proton signals at *δ*_H_ 8.09 (1H, d, *J* = 5.4 Hz, H-5), 7.80 (1H, d, *J* = 5.4 Hz, H-6), 7.46 (1H, m, H-9), 7.09 (1H, dd, *J* = 8.8, 2.4 Hz, H-11), and 7.39 (1H, d, *J* = 8.8 Hz, H-12). Furthermore, the signal for H-19 at *δ*_H_ 5.37 (1H, dd, *J* = 6.8 Hz) confirmed the presence of a double bond. The ^13^C NMR and DEPT spectra (Table 2) showed one methyl group, four methylene groups, seven methine groups, and seven quaternary carbons. The ^1^H and ^13^C NMR spectra of **5** showed a close resemblance to those of **12**, suggesting that they share the same core structure. The only difference was the additional hydroxyl substitution at C-10 in **5**, which was unambiguously confirmed by the observed HMBC correlations from H-12, H-11, and H-9 to the oxygenated carbon at C-10.

Compound **6** was characterized as a yellow powder with the molecular formula C_20_H_26_N_2_O_3_ (9 degrees of unsaturation), as assigned from the ESIHRMS signal at *m/z* 343.20145 [M + H]⁺ (calcd 343.20162). The structural features were supported by NMR data (Table 3): the ^1^H NMR spectrum indicated an indole moiety and a methyl group, while the ^13^C NMR and DEPT spectra detailed the carbon skeleton, consisting of five quaternary carbons, eight methines, six methylenes, and one methyl group. Comparing their NMR data, compound **6** had very similar structure to that of known compound, dihydro-18,19 sitsirikine [19], and the only difference in their structures was the existence of a carboxyl group in compound **6** instead of a ester group in dihydro-18,19 sitsirikine. This conclusion was confirmed by the key HMBC correlation from H-15/H-17 to C-22 (δ_C_ 179.5) (Fig. 2).

**Table 3:** ^1^H and ^13^C NMR Spectroscopic Data of Compounds **6 − 8** (CD_3_OD)

No	**6**^a^		**7**^a^		**8**^b^	
	*δ*_C_, type	*δ*_H_, mult (*J* in Hz)	*δ*_C_, type	*δ*_H_, mult (*J* in Hz)	*δ*_C_, type	*δ*_H_, mult (*J* in Hz)
2	132, C		134.6, C		137.1, C	
3	62.3, CH	3.99, t (9.5)	61.7, CH	3.22 m	61.7, CH	3.21, d (11.6)
5	53.8, CH_2_	3.53, m	54.7, CH_2_	3.04 m	54.4, CH_2_	3.09, t (5.8)
		3.12, m		2.57 td (11.7, 4.6)		2.57 td (11.5, 4.6)
6	20.8, CH_2_	3.11, m	24.4, CH_2_	3.23 m	22.3, CH_2_	2.93, m
		2.88, d (11.5)		3,03 m		2.67, dd (15.3, 4.4)
7	107.1, C		107.7, C		107.9, C	
8	127.7, C		119.5, C		128.7, C	
9	118.9, CH	7.39, d (7.6)	153.1, C		106.5, CH	7.15, d (2.4)
10	120.3, CH	6.99, t (7.6)	104.4, CH	6.67, d (7.8)	153.0, C	
11	122.9, CH	7.07, t (7.6)	122.5, CH	6.90, t (7.8)	113.7, CH	6.90, dd (8.7, 2.4)
12	112.3, CH	7.27, d (7.6)	106.8, CH	6.94, d (7.8)	112.2, CH	7.16, d (8.7)
13	138.4, C		139.7, C		134.5, C	
14	30.4, CH_2_	2.49, d (9.5)	35.7, CH_2_	2.43, d (13.0)	35.7, CH_2_	2.42, dt (13.2, 3.1)
		1.88, d (10.0)		1.19, m		1.21, m
15	39.4, CH	1.88, d (10.0)	38.1, CH	1.44, m	38.1, CH	1.4, m
16	51.2, CH	2.83, t (7.8)	36.6, CH_2_	1.98, m	36.6, CH_2_	1.96, dtd, (13.6, 7.8, 2.4)
				1.30, m		1.31, m
17	62.8, CH_2_	3.89, dd (10.9, 6.8)	60.6, CH_2_	3.72, ddd (12.7, 7.9, 4.8)	60.6, CH_2_	3.67, m
		3.67, dd (11.0, 7.8)		3.66, m		
18	10.3, CH_3_	0.94, t (7.5)	11.3, CH_3_	0.94, t (7.5)	11.3, CH_3_	0.93, t (7.6)
19	23.7, CH_2_	1.88, d (10.0)	24.5, CH_2_	1.71, m	24.5, CH_2_	1.70, dqd (15.2, 7.5, 2.3)
		1.39, dt (14.7, 7.7)		1.17, m		1.16, m
20	39.2, CH	2.02, m	42.8, CH	1.45, m	42.9, CH	1.43, m
21	59.6, CH_2_	3.46, d (11.5)	61.4, CH_2_	3.05, m	61.4, CH_2_	3.07, dd (11.6, 3.1)
		2.66, s		2.13, t (11.0)		2.13, t (10.1)
22	179.5, C					
1ʹ			102.2, CH	5.05, d (7.4)	104.4, CH	4.81, m
2ʹ			75.3, CH	3.48, dd (9.2, 7.4)	75.2, CH	3.43, m
3ʹ			78.5, CH	3.45, t (8.6)	78.1, CH	3.43, m
4ʹ			71.4, CH	3.37, m	71.5, CH	3.37, m
5ʹ			78.1, CH	3.40, m	78.1, CH	3.37, m
6ʹ			62.6, CH_2_	3.86, dd (12.1, 2.2)	62.6, CH_2_	3.87, d (11.9)
				3.68, d (5.3)		3.69, m

^a^Measured on 600/150 MHz. ^b^Measured on 600/125 MHz

The molecular formula of compound **7**, a yellow amorphous powder, was established as C_25_H_36_N_2_O_7_ (9 degrees of unsaturation) from high-resolution mass data. The ESIHRMS spectrum showed an [M + H]⁺ ion at *m/z* 477.25934, in excellent agreement with the calculated value of 477.25953 for C_25_H_37_N_2_O_7_⁺. The ^1^H NMR spectrum (Table 3) displayed characteristic signals consistent with a monosubstituted indole system, including resonances at *δ*_H_ 6.67 (1H, d, *J* = 7.8 Hz), 6.90 (1H, t, *J* = 7.8 Hz), and 6.94 (1H, t, *J* = 7.8 Hz), along with a methyl signal at *δ*_H_ 0.94 (3H, d, *J* = 7.5 Hz). The ^13^C NMR and DEPT spectra revealed the presence of five quaternary carbons, eleven methines, eight methylenes, and one methyl group. Careful analysis of the NMR data revealed that the structure of compound **7** was highly similar to that of **6**, with the key structural differences being the absence of the carboxyl group and the appearance of a glycosyl substitution at C-9 in compound **7**. This presumption was confirmed by the ^1^H-^1^H COSY correlations of H-3/H-14/H-15/H-16/H-17 and the key HMBC correlation from the anomeric proton H-1' to C-9 (*δ*_C_ 153.1). The *J* values (*J* = 7.4 Hz) of the anomeric proton revealed a *β*-configuration of the glucose residue. Furthermore, the stereocenters at C-3, C-15 and C-20 in **7** were assigned as identical to those in **6**, consistent with a biosynthetic pathway that does not involve bond cleavage at these positions.

The molecular formula of compound **8**, a yellow amorphous powder, was determined to be C_25_H_36_N_2_O_7_, corresponding to 9 degrees of unsaturation. This assignment was supported by ESIHRMS, which displayed an [M + H]⁺ ion at *m/z* 477.26126 (calcd 477.25953 for C_25_H_36_N_2_O_7_⁺). The ^1^H and ^13^C NMR data (Table 3) of **8** closely resembled those of **7**, indicating an identical core structure. The key difference was the position of the glycosyl substitution, which was located at C-10 in **8** instead of C-9 as in **7**. This hypothesis was supported by the ^1^H-^1^H COSY correlations of H-11/H-12 and the key HMBC correlation from the anomeric proton H-1' to C-10 (*δ*_C_ 153.0).

The molecular formula of compound **9**, a yellow amorphous powder, was determined to be C_38_H_38_N_2_O_14_, consistent with 21 degrees of unsaturation. This assignment was supported by ESIHRMS, which displayed an [M + H]⁺ ion at *m/z* 747.24042 (calcd 747.23958 for C_38_H_38_N_2_O_14_⁺). The ^1^H NMR spectra of **9** showed two methoxy protons at δ_H_ 2.85 (3H, s, H-24) and 3.43 (3H, s H-16'). The ^13^C NMR and DEPT spectra (Table 4) of **9** showed two methoxy groups (*δ*_C_ 55.8, 51.4), three methylenes, twenty methines, and thirteen quaternary carbons. Careful analysis of the NMR data revealed that **9** was very similar to 6'-trans-feruloyl-6'-lyaloside [20]. The key structural difference between the two compounds is the addition of a carboxyl group at C-5 in compound **10**. This assignment was supported by HMBC correlations from H-6 to C-24 (δ_C_ 166.7) in spectrum. Given the close biogenetic relationship and shared biosynthetic origin between compound** 9** and 6'-trans-feruloyl-6'-lyaloside, their stereochemical structures were inferred to be identical. Consequently, the absolute configuration of **9** was thus assigned as 15*S*,19*S*,20*R*.

**Table 4:** ^1^H and ^13^C NMR Spectroscopic Data of Compound **9** (CD_3_OD)

No	**9**		No	**9**	
	*δ*_C_, type	*δ*_H_, mult (*J* in Hz)		*δ*_C_, type	*δ*_H_, mult (*J* in Hz)
2	136.6, C		23	168.3, C	
3	150.0, C		25	166.7, C	
5	137.9, C		24	51.4, CH_3_	2.85, s
6	146.3, CH	8.70, s	1'	101.7, CH	4.75, d (7.8)
7	133.1, C		2'	74.4, CH	3.29, m
8	122.4, C		3'	77.9, CH	3.45, m
9	123.8, CH	8.29, d (7.9)	4'	72.5, CH	3.35, m
10	122.5, CH	7.40, t (7.9)	5'	76.0, CH	3.64, td (9.1, 2.6)
11	131.8, CH	7.72, t (7.9)	6'	64.2, CH_2_	4.85, d (9.8)
12	113.4, CH	7.67, d (7.9)			4.33, dd (11.9, 2.5)
13	144.5, C		7'	168.9, C	
14	34.8, CH_2_	3.45, t (9.1)	8'	115.0, CH	6.39, d (15.9)
15	37.1, CH	3.36, t (5.7)	9'	147.3, CH	7.55 d (15.9)
16	109.2, C		10'	126.6, C	
17	155.4, CH	7.46, s	11'	110.3, CH	6.69, d (2.2)
18			12'	148.6, C	
19	97.9, CH	5.75, d (9.2)	13'	150.0, C	
20	45.5, CH	2.64, td (8.7, 4.9)	14'	115.5, CH	5.92, dd (8.2, 2.0)
21	135.1, CH	5.92, m	15'	123.6, CH	6.64, dd (8.2, 2.0)
22	120.0, CH_2_	5.30, dd (13.8,7.7)	16'	55.8, CH_3_	3.43, s

Measured on 600/150 MHz

Compound **10** was obtained as yellow amorphous powder, and its molecular formula was confirmed as C_39_H_42_N_4_O_6_ from ESIHRMS at *m/z* 663.31771 [M + H]^+^ (calcd. for C_39_H_43_N_4_O_6_^+^, 663.31885), with 21 degrees of unsaturation. The ^1^H NMR data (Table 5) showed signals for nineteen aromatic protons, three N-methyl groups, and four amino methines. The ^13^C NMR spectrum revealed signals consistent with twenty-four aromatic carbons, four carbonyl groups, five methines, three methylenes, and three methyl groups. Through detailed analysis of the spectroscopic data, the structure of 11 was elucidated and found to be highly similar to that of ophiorrhisine A [21], a compound isolated from *Ophiorrhiza* nutans. The key structural difference was the lack of a hydroxyl substitution at the C-28 in compound **10**.

**Table 5:** ^1^H and ^13^C NMR Spectroscopic Data of Compound **10** (CD_3_OD)

No	**10**		No	**10**	
	*δ*_C_, type	*δ*_H_, mult (*J* in Hz)		*δ*_C_, type	*δ*_H_, mult (*J* in Hz)
1	156.6, C		20	130, CH	7.33, m
3	81.4, CH	5.78, d (9.1)	22	165.6, C	
4	58.3, CH	4.99, d (9.1)	23	75.3, CH	4.41, d (8.4)
5	169.8, C		24	34.6, CH_2_	3.11, dd (14.7, 6.2)
7	54.4, CH	4.64, dt (7.4, 3.9)			2.87, dd (14.7, 7.9)
8	170, C		25	135.9, C	
10	57.4, CH	4.77, dd (11.2, 6.7)	26	130.3, CH_2_	7.17, m
11	40.8, CH_2_	3.51, dd (13.4, 6.7)	27	130.1, CH_2_	7.18, m
		2.71, dd (13.4, 11.1)	28	128.6, CH	7.07, tt (6.6, 2.0)
12	133.3, C		29	40.3, CH_2_	2.58, dd (14.4, 6.7)
13	131.9, CH	7.15, m			2.43, dd (14.4, 6.7)
14	114.8, CH	7.13, m	30	137.6, C	
15	120.4, CH	6.84, m	31	130.2, CH_2_	6.85, m
16	133.7, CH	6.85, m	32	129.2, CH	6.91, t (7.6)
17	139.7, C		33	127.4, CH	6.72, t (7.3)
18	130.4, CH_2_	7.61, d (7.0)	34	178.1, C	
19	129.6, CH_2_	7.37, dd (8.1, 6.7)	35	52.8, CH_3_	2.64, s

Measured on 600/150 MHz

In addition to the new compounds mentioned above, 13 known compounds were obtained and identified as deppeaninol (**11**) [22], (–)-dihydrocorynanthenol (**12**) [23], desoxycordifoline (**13**) [24], 3*α*-5*α*-tetrahydrodeoxycordifoline lactam (**14**) [25], strictosamide (**15**) [26], vincosamide (**16**) [27], desoxycordifoline (**17**) [28], lyaloside (**18**) [29], lyalosidie acid (**19**) [30], strictosidinic acid (**20**) [31], 5*α*-carboxystrictosidine (**21**) [32], deoxystrictosamide (**22**) [33], and turbinatine (**23**) [34], by comparison with literature data.

The immunoactivity of the compounds **1** and **3–10** was determined by ^3^H-TdR incorporation method. The level of lymphocyte response to the stimulus could be inferred based on the amount of isotope incorporated into the cell (as measured by liquid scintillation) and compared with the active control drug immunosuppressant cyclosporin A (CsA), and the results were shown in Table 6. Based on a comprehensive analysis of cytotoxicity (MTT assay) and immunomodulatory activity, compounds **3–5** and **9** strongly inhibited lipopolysaccharide-induced B cell proliferation, with IC_50_ values ranging from 3.6 to 9.1 µM, and demonstrated excellent selectivity (SI > 10). Meanwhile, compounds **1**, **3–5**, and **10** exhibited moderate inhibitory activity against concanavalin A-induced T cell proliferation, with IC_50_ values from 15.7 to 58.6 μM.

**Table 6:** Lymphocyte toxicity and proliferative inhibitory activity of isolated compounds

Compounds	CC_50_ (μM)	ConA IC_50_ (μM)	SI	LPS IC_50_ (μM)	SI
**1**	40.5	15.7	2.6	14.8	2.7
**3**	57.4	44.1	1.3	3.6	15.8
**4**	> 100	23.9	> 4	8.4	> 11.8
**5**	> 100	58.6	> 1	9.1	> 10
**6**	> 100	> 100	/	22.4	> 4
**7**	/	/	/	> 100	/
**8**	/	13.8	/	/	/
**9**	71.7	> 100	< 1	5.3	13.5
**10**	96.2	49.8	1.9	14.6	6.6
CSAa	2.9	0.003	> 200	0.123	23.2

^a^Positive control

## Experimental section

*General Experimental Procedures* NMR spectroscopic data were acquired using Bruker Avance III 600 MHz and 500 MHz spectrometers. UV measurements were conducted on a UH-5300 UV–vis spectrophotometer. CD profiles were obtained with a Chirascan-plus circular dichroism spectrometer. Column chromatography (CC) was carried out with silica gel (Qingdao Marine Chemical Ltd., China), RP-18 gel (Fuji Silysia Chemical Ltd., Japan), and Sephadex LH-20 (Pharmacia Fine Chemical Co., Ltd., Sweden). TLC analysis was performed on GF 254 plates (Qingdao Haiyang Chemical Co., Ltd., China), and spots were visualized using Dragendorff’s reagent. Medium-pressure liquid chromatography (MPLC) separations were executed on a Biotage SP1 system. HPLC purification was conducted using an Agilent 1260 series system equipped with analytical semi-preparative or preparative Sunfire C_18_ columns (dimensions: 4.6 × 150 mm and 19 × 250 mm, respectively).

*Plant Material* *Ophiorrhiza brevidentata* H. S. Lo was collected from Baoshan and Tengchong County, Yunnan Province, P. R. China in March 2022, and was identified by Dr. Honglian Ai. The whole plant specimens were kept at School of Pharmaceutical Sciences, South-Central MinZu University.

*Extraction and isolation* The whole plants (14.5 kg) after crushing were soaking in 90% MeOH (20 L × 5) for a week. The solvent was subsequently removed by a rotary evaporator. The obtained samples were dissolved in water,adjusted pH to 2–3 with weak acids and extracted ( each one three times) with petroleum ether, petroleum ether: ethyl acetate = 1:1 and ethyl acetate respectively. The pH was adjusted to 7–8 after removing the organic layer, and extracted three times with ethyl acetate.This part of the ethyl acetate was collected, and the solvent was removed to obtain the sample, which weighed 32 g. The final ethyl acetate extract was divided into five fractions (A-E) by pass through silica gel (200–300 mesh) column chromatography with the elution gradient of CHCl_3_-CH_3_OH ( 1:0- 0:1).

Fraction B (9 g) was subjected to MPLC separation using a MeOH–H_2_O gradient (5:95 to 100:0, v/v), yielding four subfractions (B-1 to B-4). Subfraction B-1 was further fractionated on Sephadex LH-20 (eluent: MeOH) to afford eight components (B-1–1 to B-1–8). Compound **1** (2.2 mg, *t*_R_ = 28 min) was obtained from B-1–6 by purification on a preparative C_18_ HPLC column with an MeCN–H_2_O gradient (10:90 to 30:70, v/v). Fraction B-2 was separated on Sephadex LH-20 (MeOH) into four subfractions. Purification of B-2–2 via preparative C_18_ HPLC (MeCN–H_2_O, 10:90 to 30:70, v/v) afforded compound **9** (9.9 mg, *t*_R_ = 25 min). Fraction B-4 was chromatographed on Sephadex LH-20 (MeOH) to yield four subfractions. B-4–1 was further separated on Sephadex LH-20 (MeOH) into five parts. Purification of B-4–1-4 by preparative C_18_ HPLC (MeCN–H_2_O, 10:90 to 30:70, v/v) yielded compound **18** (10 mg, *t*_R_ = 28 min). B-4–2 was purified using the same type of column (MeCN–H_2_O, 10:90 to 40:60, v/v) to give compounds **4** (2.6 mg, *t*_R_ = 41 min) and **11** (3.2 mg, *t*_R_ = 44 min). B-4–4 was fractionated by silica gel (200–300 mesh) column chromatography with a CHCl_3_–MeOH gradient (20:1 to 0:1, v/v), yielding four subfractions. B-4–4-2 was purified by preparative C_18_ HPLC (MeCN–H_2_O, 20:80 to 40:60, v/v) to afford compound **3** (2.5 mg, *t*_R_ = 34 min).Similarly, B-4–4-4 was processed under analogous conditions (MeCN–H_2_O, 10:90 to 35:65, v/v) to yield compound **2** (1.2 mg, *t*_R_ = 28 min).

Fraction C (4 g) was fractionated by MPLC using a MeOH–H_2_O gradient (5:95 to 100:0, v/v), yielding five subfractions (C-1 to C-5). Subfraction C-2 was further separated on Sephadex LH-20 (eluted with MeOH) into six fractions. C-2–2 was purified by preparative C18 HPLC (MeCN–H_2_O, 25:75 to 50:50, v/v) to afford compound **5** (1.0 mg, *t*_R_ = 19 min), while C-2–3 was processed under similar conditions (MeCN–H_2_O, 30:70 to 50:50, v/v) to yield compound **12** (3.0 mg, *t*_R_ = 36 min). C-3 was subjected to MPLC with a MeOH–H_2_O gradient (5:95 to 100:0, v/v), yielding five subfractions (C-3–1 to C-3–5). Purification of C-3–2 by preparative C_18_ HPLC (MeCN–H_2_O, 10:90 to 30:70, v/v) afforded compounds **14** (1.5 mg, *t*_R_ = 18 min), **15** (24 mg, *t*_R_ = 44 min), and **16** (5 mg, *t*_R_ = 49.5 min). C-4 was chromatographed on Sephadex LH-20 (MeOH) to give seven fractions. From C-4–6, compound **13** (18 mg, *t*_R_ = 25 min) was obtained after purification via preparative C_18_ HPLC (MeCN–H₂O, 10:90 to 30:70, v/v). C-5 was fractionated on Sephadex LH-20 (MeOH) into seven subfractions. C-5–6 was purified using a preparative C_18_ HPLC column (MeCN–H_2_O, 10:90 to 30:70, v/v) to yield compound **17** (10 mg, *t*_R_ = 23 min).

Fraction D (11 g) was fractionated by MPLC using a MeOH–H_2_O gradient (5:95 to 100:0, v/v) to afford five subfractions (D-1 to D-5). Subfraction D-3 was chromatographed on Sephadex LH-20 (eluent: MeOH), yielding seven fractions. Purification of D-3–2 by preparative C18 HPLC (MeCN–H_2_O, 15:85 to 50:50, v/v) afforded compound **6** (2.1 mg, *t*_R_ = 21 min). Similarly, D-3–6 was processed under a gradient of MeCN–H_2_O (10:90 to 30:70, v/v) to give compound **10** (9.2 mg, *t*_R_ = 38 min). D-4 was separated on Sephadex LH-20 (MeOH) into four fractions. From D-4–2, compound **22** (5.1 mg, *t*_R_ = 27 min) was obtained after purification via preparative C18 HPLC (MeCN–H_2_O, 10:90 to 30:70, v/v). D-5 was fractionated on Sephadex LH-20 (MeOH) to give ten subfractions. D-5–3 was purified using a preparative C18 HPLC column (MeCN–H_2_O, 10:90 to 30:70, v/v) to yield compounds **7** (1.0 mg, *t*_R_ = 51 min) and **8** (3.8 mg, *t*_R_ = 56 min). D-5–5 was subjected to the same type of column (MeCN–H_2_O, 10:90 to 20:80, v/v) to afford compounds **20** (75 mg, *t*_R_ = 29 min) and **21** (1.2 mg, *t*_R_ = 24 min). Additionally, D-5–8 was purified under conditions of MeCN–H_2_O (10:90 to 30:70, v/v) to give compounds **23** (43 mg, *t*_R_ = 30 min) and **19** (75 mg, *t*_R_ = 34 min).

Ophiorbrevine A **(1):** yellow powder; $[\alpha]_{\text{D}}^{19}$-2.0 (*c* 0.50, MeOH);); UV (MeOH) *λ*_max_ (log *ε*) 235 (4.43), 260 (4.41), 325 (4.17) nm; the ^1^H (600 MHz) and ^13^C NMR (150 MHz) data (DMSO), see Table 1; HRMS(ESI) *m/z*: [M + H]^+^ calcd. for C_22_H_19_O_5_N_2_ 391.12885; Found 391.19910.

Ophiorbrevine B **(2):** orange powder, $[\alpha]_{\text{D}}^{19}$ + 165.0 (*c* 0.50, MeOH); UV (MeOH) *λ*_max_ (log *ε*) 210 (3.86), 240 (3.89), 270 (3.96) nm; the ^1^H (600 MHz) and ^13^C NMR (150 MHz) data (DMSO), see Table 1; HRMS(ESI) *m/z*: [M + H]^+^ calcd. for C_22_H_20_O_4_N_2_ 390.14483; Found 390.14465.

Ophiorbrevine C **(3):** yellow powder, $[\alpha]_{\text{D}}^{23}$ ‒268.0 (*c* 0.50, MeOH); UV (MeOH) *λ*_max_ (log *ε*) 225 (4.38), 270 (3.87) nm; the ^1^H (600 MHz) and ^13^C NMR (150 MHz) data (CDCl_3_), see Table 1; HRMS(ESI) *m/z*: [M + H]^+^ calcd. for C_19_H_20_N_3_ 290.16517; Found 290.16501.

Ophiorbrevine D **(4):** yellow powder, $[\alpha]_{\text{D}}^{22}$ ‒8.0 (*c* 0.50, MeOH); UV (MeOH) *λ*_max_ (log *ε*) 235 (4.44), 285 (4.04), 340 (3.65) nm; the ^1^H (600 MHz) and ^13^C NMR (150 MHz) data (CD_3_OD), see Table 2; HRMS(ESI) *m/z*: [M + H]^+^ calcd. for C_19_H_23_N_2_O_2_ 311.17540; Found 311.17517.

Ophiorbrevine E **(5):** yellow powder, $[\alpha]_{\text{D}}^{23}$ + 210.0 (*c* 0.50, MeOH); UV (MeOH) *λ*_max_ (log *ε*) 235 (4.33), 295 (4.10) nm; the ^1^H (600 MHz) and ^13^C NMR (150 MHz) data (CD_3_OD), see Table 2; HRMS(ESI) *m/z*: [M + H]^+^ calcd. for C_19_H_23_N_2_O_3_ 327.17032; Found 327.17010.

Ophiorbrevine F **(6):** yellow powder, $[\alpha]_{\text{D}}^{23}$ ‒42.0 (*c* 0.50, MeOH); UV (MeOH) *λ*_max_ (log *ε*) 220 (4.37), 275 (3.79) nm; the ^1^H (600 MHz) and ^13^C NMR (150 MHz) data (CD_3_OD), see Table 3; HRMS(ESI) *m/z*: [M + H]^+^ calcd. for C_20_H_27_O_3_N_2_ 343.20162; Found 343.20145.

Ophiorbrevine G **(7):** yellow powder, $[\alpha]_{\text{D}}^{23}$ + 42.0 (*c* 0.50, MeOH); UV (MeOH) *λ*_max_ (log *ε*) 225 (3.72), 270 (3.15) nm; the ^1^H (600 MHz) and ^13^C NMR (150 MHz) data (CD_3_OD), see Table 3; HRMS(ESI) *m/z*: [M + H]^+^ calcd. for C_25_H_37_O_7_N_2_ 477.25953; Found 477.25934.

Ophiorbrevine H **(8):** yellow powder, $[\alpha]_{\text{D}}^{22}$ ‒20.0 (*c* 0.50, MeOH); UV (MeOH) *λ*_max_ (log *ε*) 225 (4.39), 285 (3.90) nm; the ^1^H (600 MHz) and ^13^C NMR (150 MHz) data (CD_3_OD), see Table 3; HRMS(ESI) *m/z*: [M + H]^+^ calcd. for C_25_H_37_O_7_N_2_ 477.25953; Found 477.26126.

Ophiorbrevine I **(9):** yellow powder, $[\alpha]_{\text{D}}^{22}$ ‒106.0 (*c* 0.50, MeOH); UV (MeOH) *λ*_max_ (log *ε*) 240 (4.25), 270 (4.22), 310 (4.03) nm; the ^1^H (600 MHz) and ^13^C NMR (150 MHz) data (CD_3_OD), see Table 4; HRMS(ESI) *m/z*: [M + H]^+^ calcd. for C_38_H_39_O_14_N_2_ 747.23958; Found 747.24042.

Ophiorbrevine J **(10):** yellow powder, $[\alpha]_{\text{D}}^{23}$ ‒54.0 (*c* 0.50, MeOH); UV (MeOH) *λ*_max_ (log *ε*) 215 (4.59) nm; the ^1^H (600 MHz) and ^13^C NMR (150 MHz) data (CD_3_OD), see Table 5; HRMS(ESI) *m/z*: [M + H]^+^ calcd. for C_39_H_43_N_4_O_6_ 663.31885; Found 663.31771.

*Quantum Chemical Calculations* Conformational analyses and ECD calculations were performed using Gaussian 16, Spartan'14, and Chem3D. After determining the relative configuration of the compound according to NMR and MS, the absolute configuration was determined by quantum computing method. Firstly, Spartan'14 was used to search for the dominant conformations of compounds, and the conformational optimization and frequency of multiple low-energy dominant conformations were calculated at the B3LYP/def2svp theoretical level in Gaussian 16. The conformation with a relative energy of less than 3 kcal/mol calculated by the Boltzmann distribution formula was used to calculate the CD using the IEF-PCM solvent model (MeOH) at the theoretical level of wB97XD/Def2sVP. ECD curves of calculated and experimental values were generated by fitting them to SpecDis-1701 software.

*Immunosuppressive activity Assays* In this experiment, the non-specific toxicity and proliferation response of mouse lymphocytes were used as a model. The spleens of the mice sacrificed by devertebration were removed and used to prepare a single cell suspension. Red blood cell lysate was used to remove red blood cells and then cell concentration was regulated to obtain different concentrations of mouse spleen lymphocytes required for the experiment.

MTT (Methylthiazolyldiphenyl-tetrazolium bromide) method was used to detect the effect of compounds on the activity of mouse spleen lymphocytes. Mouse spleen lymphocyte suspension (8 × 10^5^/well) was inoculated in 96-well plates, and different concentrations (initial screening concentration: 100, 10, 1 μM, resieve concentration: 100, 50, 25, 12.5, 6.25, 3.125, 1.5625 μM) of compounds were added to a total volume of 200 *μ*L. Positive control drug was immunosuppressant cyclosporin A (CsA, 20, 10, 5, 2.5, 1.25, 0.625, 0.313, 0.156, 0.078, 0.039 μM), cell control was the corresponding solvent, and blank control was culture medium. MTT solution (5 mg/ml) was added 4 h before the end of the culture (37 ℃, 5% CO_2_, 48 h). Purple crystals Formazan dissolved in DMSO were added to the plate (150 *μ*L/well) at the end of the culture after removing the supernatant and the OD value was determined under a microplate reader (570 nM).

The effect of compounds on the proliferation of T and B lymphocytes in the spleen of mice was detected by ^3^H-TdR incorporation method. Mouse spleen lymphocyte suspension (5 × 10^5^/well) was inoculated in 96-well plates, ConA (final concentration 1 μg/ml) or LPS (final concentration 10 μg/ml), and different concentrations (initial screening concentration: 100, 10, 1 μM, resieve concentration: 100, 50, 25, 12.5, 6.25, 3.125, 1.5625 μM) of compounds were added to a total volume of 200 *μ*L. Positive control drug was immunosuppressant cyclosporin A ( CsA, 10, 5, 2.5, 1.25, 0.625, 0.313, 0.156, 0.078, 0.039, 0.019 μM), cell control was the corresponding solvent without ConA and LPS, and Stimulation of the drug-free control group. ^3^H-thymidine nucleotide (25*μ*L/well, 10 *μ* Ci/ml) was added 8 h before the end of the culture (37 ℃, 5% CO_2_, 48 h). Scintillation fluid were added to the cells collected by a cell collector on the fiberglass membrane at the end of the culture and the amount of ^3^H-TdR incorporated into the DNA of the cell was recorded under a Beta register. The condition of cell proliferation was expressed by cpm values.

Cell viability (%) = (OD_1_—OD_3_) / ( OD_2_—OD_3_) × 100%, OD_1_ was mean of the drug group, OD_2_ was mean of the cell control, and OD_3_ was the mean of blank control. Inhibition of cell proliferation (%) = (1- ( cpm_1_—cpm_3_) / (cpm_2_-cpm_3_)) × 100%, cpm_1_ was mean of the drug group, cpm_2_ was mean of the stimulated control, and cpm_3_ was mean of the cell control. Selection index (SI) = CC_50_ ( 50% Cytotoxic concentration) / IC_50_ ( 50% inhibitory concentration). SI greater than 10 is considered to have a biological effect. All index check results were processed using Excel 2016 and Graphpad Prism 9.

## Conclusion

In conclusion, ten new monoterpene indole alkaloids (**1–10**) and 13 known compounds (**11–23**) were isolated from *O. brevidentata*. The structures of all compounds were elucidated through comprehensive spectroscopic analysis, including NMR spectroscopy, high-resolution mass spectrometry, and quantum chemical calculations. Compound **1** features an unprecedented skeleton characterized by a fused 6/5/6/7/6 pentacyclic ring system. Compounds **3–5** and **9** displayed potent inhibitory activity against lipopolysaccharide-induced B cell proliferation, exhibiting IC_50_ values between 3.6 and 9.1 µM along with excellent selectivity (SI > 10), indicating their potential as immunosuppressive agents.

## Supplementary Information

Supplementary file 1.

## Data Availability

All data generated and analyzed during this study are included in this published article and its Additional file.
