# Supplementary material for: Monoterpene indole alkaloids from the aerial parts of Ophiorrhiza brevidentata and their immunological activities
Source: Nat Prod Bioprospect. 2026 Jan 11;16(1):20. doi: 10.1007/s13659-025-00575-y (PMC12790544; doi:10.1007/s13659-025-00575-y)
Supplement: Supplementary file 1 [file 13659_2025_575_MOESM1_ESM.docx]

**Monoterpene Indole Alkaloids from the Aerial Parts of *Ophiorrhiza brevidentata.* H. S. Lo and Their Immunological Activities**

Fan Xu ^a,†^, Zheng-Hui Li ^a,b,†^, Meng-Lin Feng ^a^, Jia-Yu Jin ^a^, Bao-Bao Shi ^a,b,⁎^ and Ji-Kai Liu ^a,b,⁎^

^a^School of Pharmaceutical Sciences, South-Central Minzu University, Wuhan 430074, People’s Republic of China

^b^International Cooperation Base for Active Substances in Traditional Chinese Medicine in Hubei Province, School of Pharmaceutical Sciences, South-Central Minzu University

*Corresponding authors.
E-mail addresses: 2021068@mail.scuec.edu.cn (B.-B. Shi);

liujikai@mail.scuec.edu.cn (J.-K. Liu).

^†^These authors contributed equally to this work.

Table of Contents

[**Supplementary of NMR, HRESIMS and CD spectra** 3](#_Toc207911300)

[**Figure S1.1 ^1^H NMR spectrum of 1.** 3](#_Toc207911301)

[**Figure S1.2 ^13^C NMR spectrum of 1** 3](#_Toc207911302)

[**Figure S1.3 HSQC spectrum of 1** 4](#_Toc207911303)

[**Figure S1.4 HMBC spectrum of 1** 4](#_Toc207911304)

[**Figure S1.5 COSY spectrum of 1** 5](#_Toc207911305)

[**Figure S1.6 ROESY spectrum of 1** 5](#_Toc207911306)

[**Figure S1.7 HRMS spectrum of 1** 6](#_Toc207911307)

[**Figure S1.8 CD spectrum of 1** 6](#_Toc207911308)

[**Figure S2.1 ^1^H NMR spectrum of 2.** 7](#_Toc207911309)

[**Figure S2.2 ^13^C NMR spectrum of 2.** 7](#_Toc207911310)

[**Figure S2.3 HSQC spectrum of 2** 8](#_Toc207911311)

[**Figure S2.4 HMBC spectrum of 2** 8](#_Toc207911312)

[**Figure S2.5 COSY spectrum of 2.** 9](#_Toc207911313)

[**Figure S2.6 ROESY spectrum of 2** 9](#_Toc207911314)

[**Figure S2.7 HRMS spectrum of 2** 10](#_Toc207911315)

[**Figure S2.8 CD spectrum of 2** 10](#_Toc207911316)

[**Figure S3.1 ^1^H NMR spectrum of 3** 11](#_Toc207911317)

[**Figure S3.2 ^13^C NMR spectrum of 3** 11](#_Toc207911318)

[**Figure S3.3 HSQC spectrum of 3** 12](#_Toc207911319)

[**Figure S3.4 HMBC spectrum of 3** 12](#_Toc207911320)

[**Figure S3.5 COSY spectrum of 3** 13](#_Toc207911321)

[**Figure S3.6 ROESY spectrum of 3** 13](#_Toc207911322)

[**Figure S3.7 HRMS spectrum of 3** 14](#_Toc207911323)

[**Figure S4.1 ^1^H NMR spectrum of 4** 15](#_Toc207911324)

[**Figure S4.2 ^13^C NMR spectrum of 4** 15](#_Toc207911325)

[**Figure S4.3 HSQC spectrum of 4** 16](#_Toc207911326)

[**Figure S4.4 HMBC spectrum of 4** 16](#_Toc207911327)

[**Figure S4.5 COSY spectrum of 4** 17](#_Toc207911328)

[**Figure S4.6 ROESY spectrum of 4** 17](#_Toc207911329)

[**Figure S4.7 HRMS spectrum of 4** 18](#_Toc207911330)

[**Figure S5.1 ^1^H NMR spectrum of 5** 19](#_Toc207911331)

[**Figure S5.2 ^13^C NMR spectrum of 5** 19](#_Toc207911332)

[**Figure S5.3 HSQC spectrum of 5** 20](#_Toc207911333)

[**Figure S5.4 HMBC spectrum of 5** 20](#_Toc207911334)

[**Figure S5.5 COSY spectrum of 5** 21](#_Toc207911335)

[**Figure S5.6 ROESY spectrum of 5** 21](#_Toc207911336)

[**Figure S5.7 HRMS spectrum of 5** 22](#_Toc207911337)

[**Figure S6.1 ^1^H NMR spectrum of 6** 23](#_Toc207911338)

[**Figure S6.2 ^13^C NMR spectrum of 6** 23](#_Toc207911339)

[**Figure S6.3 HSQC spectrum of 6** 24](#_Toc207911340)

[**Figure S6.4 HMBC spectrum of 6** 24](#_Toc207911341)

[**Figure S6.5 COSY spectrum of 6** 25](#_Toc207911342)

[**Figure S6.6 ROESY spectrum of 6** 25](#_Toc207911343)

[**Figure S6.7 HRMS spectrum of 6.** 26](#_Toc207911344)

[**Figure S7.1 ^1^H NMR spectrum of 7** 27](#_Toc207911345)

[**Figure S7.2 ^13^C NMR spectrum of 7** 27](#_Toc207911346)

[**Figure S7.3 HSQC spectrum of 7** 28](#_Toc207911347)

[**Figure S7.4 HMBC spectrum of 7** 28](#_Toc207911348)

[**Figure S7.5 COSY spectrum of 7** 29](#_Toc207911349)

[**Figure S7.6 ROESY spectrum of 7** 29](#_Toc207911350)

[**Figure S7.7 HRMS spectrum of 7** 30](#_Toc207911351)

[**Figure S8.1 ^1^H NMR spectrum of 8** 31](#_Toc207911352)

[**Figure S8.2 ^13^C NMR spectrum of 8** 31](#_Toc207911353)

[**Figure S8.3 HSQC spectrum of 8** 32](#_Toc207911354)

[**Figure S8.4 HMBC spectrum of 8** 32](#_Toc207911355)

[**Figure S8.5 COSY spectrum of 8** 33](#_Toc207911356)

[**Figure S8.6 ROESY spectrum of 8** 33](#_Toc207911357)

[**Figure S8.7 HRMS spectrum of 8** 34](#_Toc207911358)

[**Figure S9.1 ^1^H NMR spectrum of 9** 35](#_Toc207911359)

[**Figure S9.2 ^13^C NMR spectrum of 9** 35](#_Toc207911360)

[**Figure S9.3 HSQC spectrum of 9** 36](#_Toc207911361)

[**Figure S9.4 HMBC spectrum of 9** 36](#_Toc207911362)

[**Figure S9.5 COSY spectrum of 9** 37](#_Toc207911363)

[**Figure S9.6 ROESY spectrum of 9** 37](#_Toc207911364)

[**Figure S9.7 HRMS spectrum of 9** 38](#_Toc207911365)

[**Figure S10.1 ^1^H NMR spectrum of 10** 39](#_Toc207911366)

[**Figure S10.2 ^13^C NMR spectrum of 10** 39](#_Toc207911367)

[**Figure S10.3 HSQC spectrum of 10** 40](#_Toc207911368)

[**Figure S10.4 HMBC spectrum of 10** 40](#_Toc207911369)

[**Figure S10.5 COSY spectrum of 10** 41](#_Toc207911370)

[**Figure S10.6 ROESY spectrum of 10** 41](#_Toc207911371)

[**Figure S10.7 HRMS spectrum of 10** 42](#_Toc207911372)

[**Figure S11.1 ^1^H NMR spectrum of 11** 43](#_Toc207911373)

[**Figure S11.2 ^13^C NMR spectrum of 11** 43](#_Toc207911374)

[**Figure S12.1 ^1^H NMR spectrum of 12** 44](#_Toc207911375)

[**Figure S12.2 ^13^C NMR spectrum of 12** 44](#_Toc207911376)

[**Figure S13.1 ^1^H NMR spectrum of 13** 45](#_Toc207911377)

[**Figure S13.2 ^13^C NMR spectrum of 13** 45](#_Toc207911378)

[**Figure S14.1 ^1^H NMR spectrum of 14** 46](#_Toc207911379)

[**Figure S14.2 ^13^C NMR spectrum of 14** 46](#_Toc207911380)

[**Figure S15.1 ^1^H NMR spectrum of 15** 47](#_Toc207911381)

[**Figure S15.2 ^13^C NMR spectrum of 15** 47](#_Toc207911382)

[**Figure S16.1 ^1^H NMR spectrum of 16** 48](#_Toc207911383)

[**Figure S16.2 ^13^C NMR spectrum of 16** 48](#_Toc207911384)

[**Figure S17.1 ^1^H NMR spectrum of 17** 49](#_Toc207911385)

[**Figure S17.2 ^13^C NMR spectrum of 17** 49](#_Toc207911386)

[**Figure S18.1 ^1^H NMR spectrum of 18** 50](#_Toc207911387)

[**Figure S18.2 ^13^C NMR spectrum of 18** 50](#_Toc207911388)

[**Figure S19.1 ^1^H NMR spectrum of 19** 51](#_Toc207911389)

[**Figure S19.2 ^13^C NMR spectrum of 19** 51](#_Toc207911390)

[**Figure S20.1 ^1^H NMR spectrum of 20** 52](#_Toc207911391)

[**Figure S20.2 ^13^C NMR spectrum of 20** 52](#_Toc207911392)

[**Figure S21.1 ^1^H NMR spectrum of 21** 53](#_Toc207911393)

[**Figure S21.2 ^13^C NMR spectrum of 21** 53](#_Toc207911394)

[**Figure S21.1 ^1^H NMR spectrum of 21** 54](#_Toc207911395)

[**Figure S22.2 ^13^C NMR spectrum of 22** 54](#_Toc207911396)

[**Figure S23.1 ^1^H NMR spectrum of 23** 55](#_Toc207911397)

[**Figure S23.2 ^13^C NMR spectrum of 23** 55](#_Toc207911398)

[**Data attribution for known compounds** 56](#_Toc207911399)

[**Computational details** 62](#_Toc207911400)

[**Table S1.** Energy analysis for conformers of **1-1~1-8** at M062X/def2svp level in the gas phase (T=298.15 K) 62](#_Toc207911401)

[**Figure S1.** Optimized conformations of **1** (the relative populations are in parentheses) 62](#_Toc207911402)

[**Figure S2.** Calculated ECD spectra for **1** at the wB97xd/def2svp level in methanol with IEFPCM model (σ = 0.35 eV). Experimental CD spectra of **1** (black line) in MeOH. 63](#_Toc207911403)

[**Table S2.** Energy analysis for conformers of **2-1~2-2** at M062X/def2svp level in the gas phase (T=298.15 K) 63](#_Toc207911404)

[**Figure S3.** Optimized conformations of **2** (the relative populations are in parentheses) 63](#_Toc207911405)

[**Figure S4.** Calculated ECD spectra for **2** at the wB97xd/def2svp level in methanol with IEFPCM model (σ = 0.35 eV). Experimental CD spectra of **2** (black line) in MeOH. 64](#_Toc207911406)

# **Supplementary of NMR, HRESIMS and CD spectra**


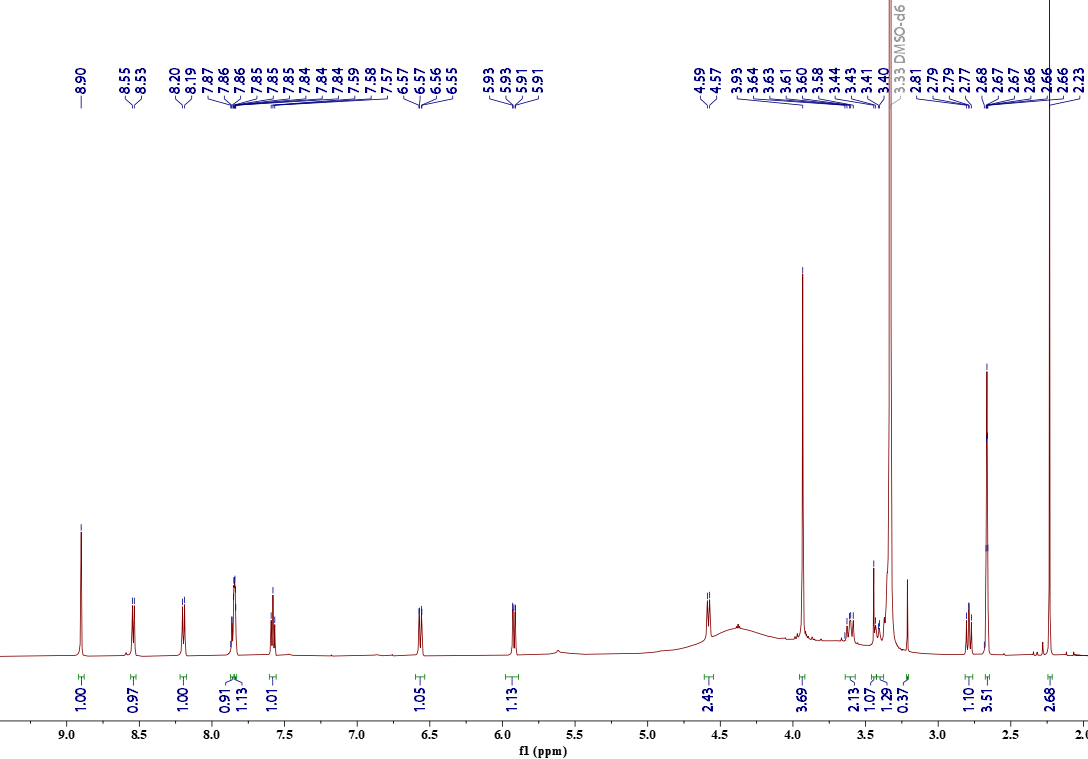


**Figure S1.1 ^1^H NMR spectrum of 1.**


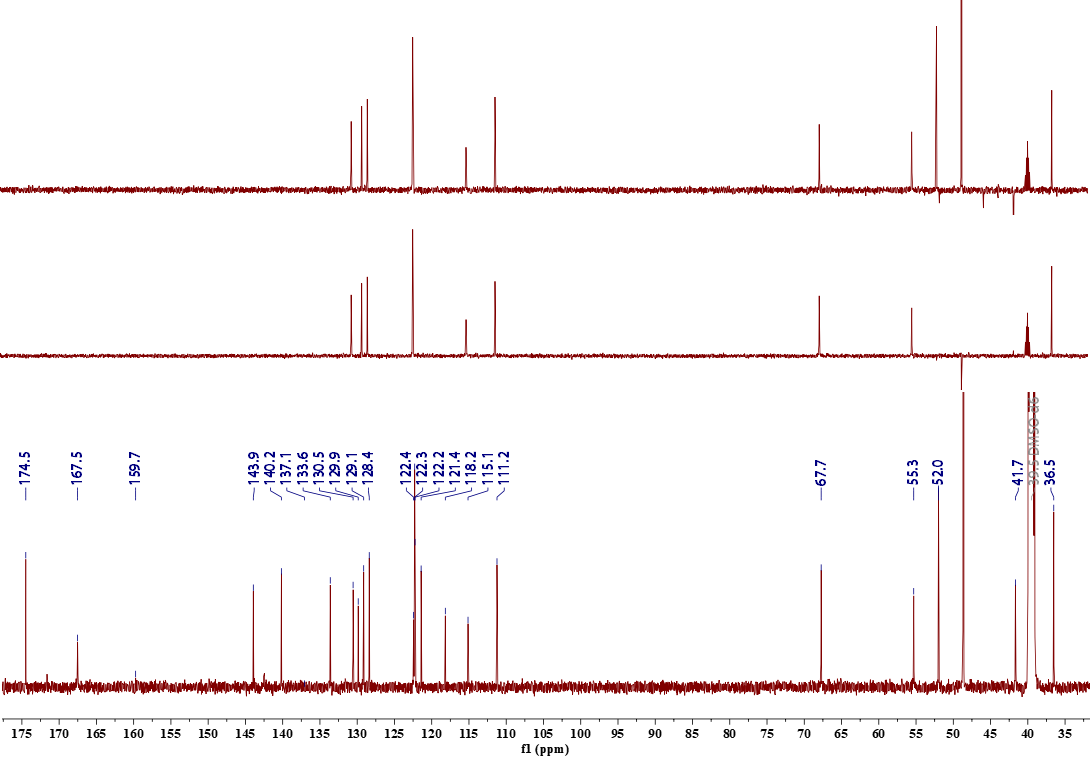


**Figure S1.2 ^13^C NMR spectrum of 1**


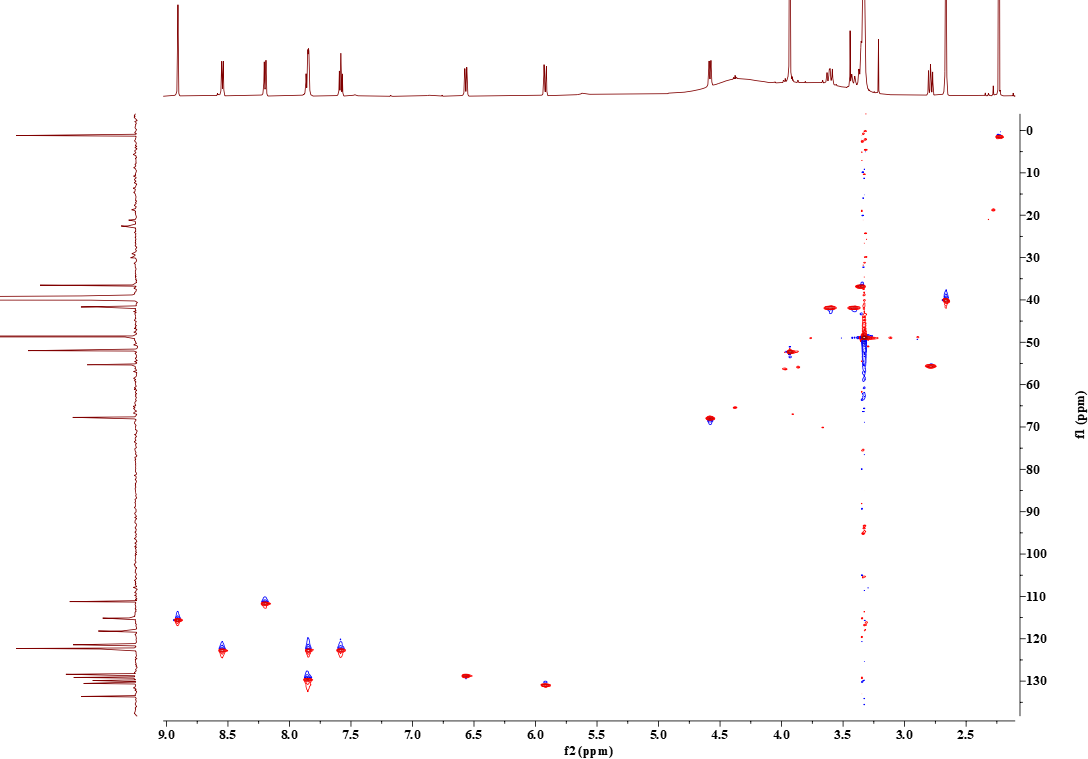


**Figure S1.3 HSQC spectrum of 1**


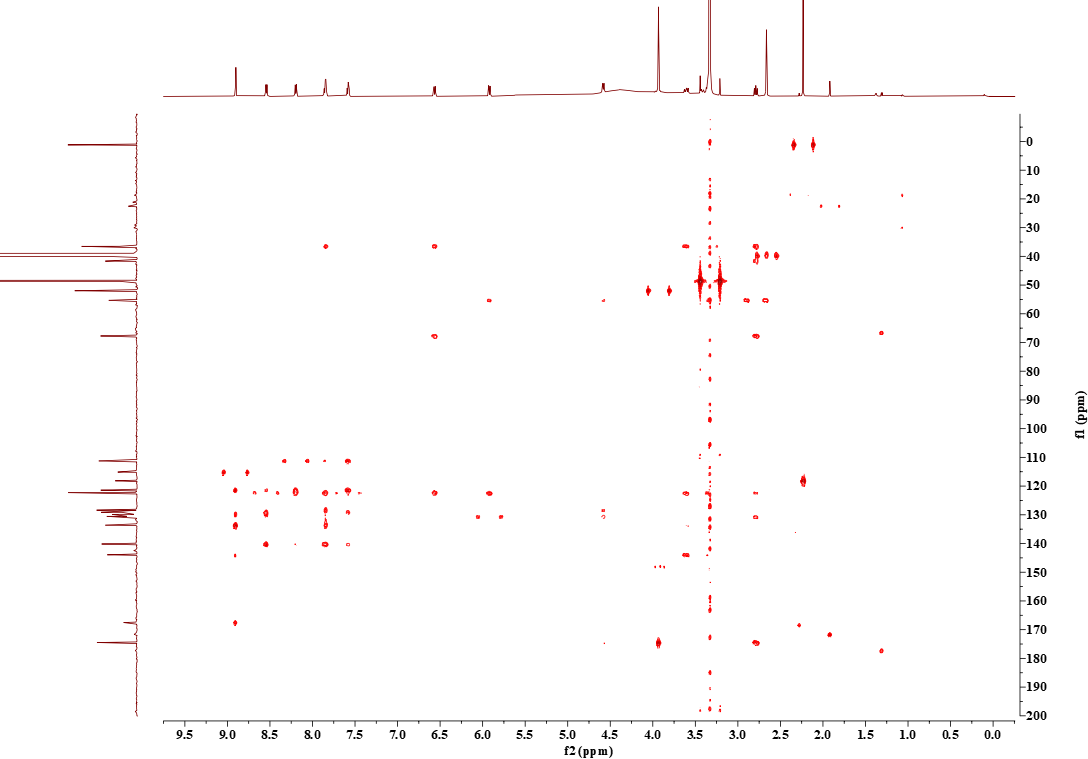


**Figure S1.4 HMBC spectrum of 1**


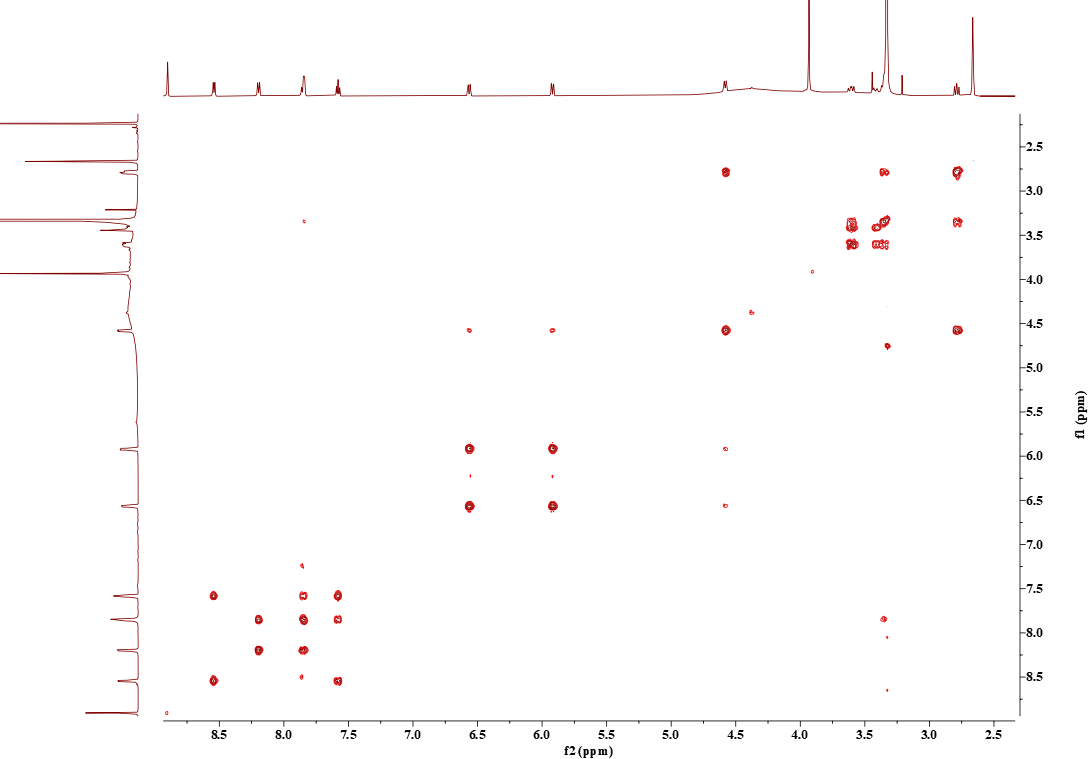


**Figure S1.5 COSY spectrum of 1**


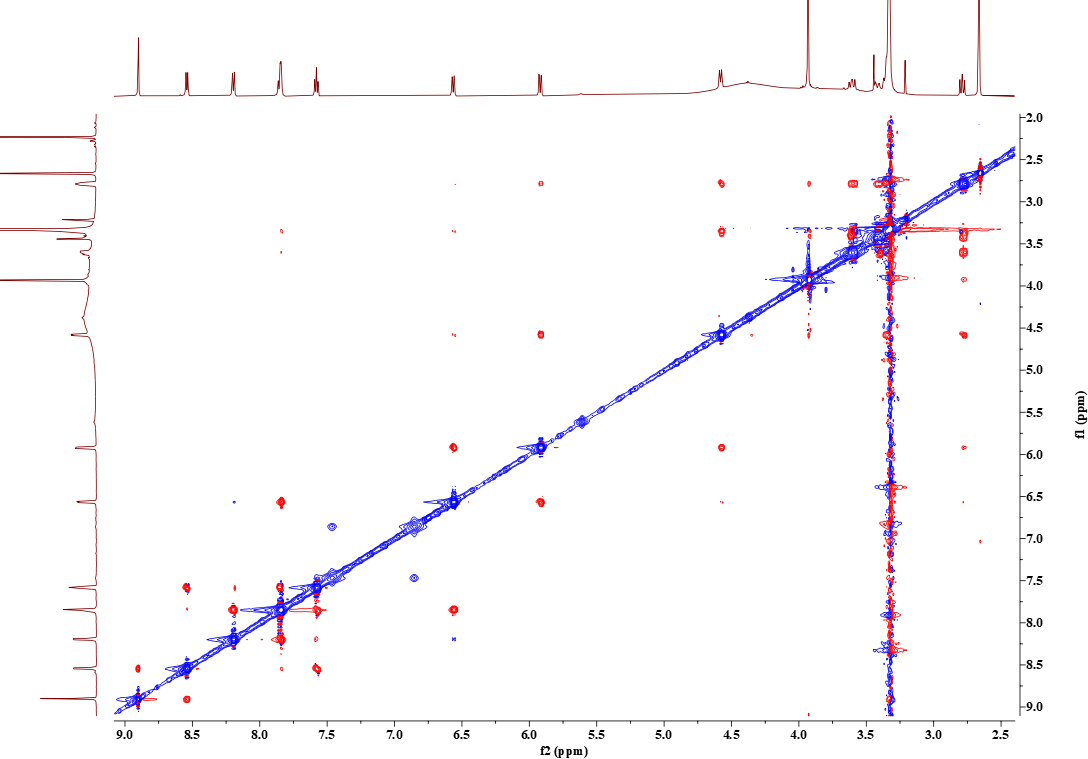


**Figure S1.6 ROESY spectrum of 1**

**Figure S1.7 HRMS spectrum of 1**


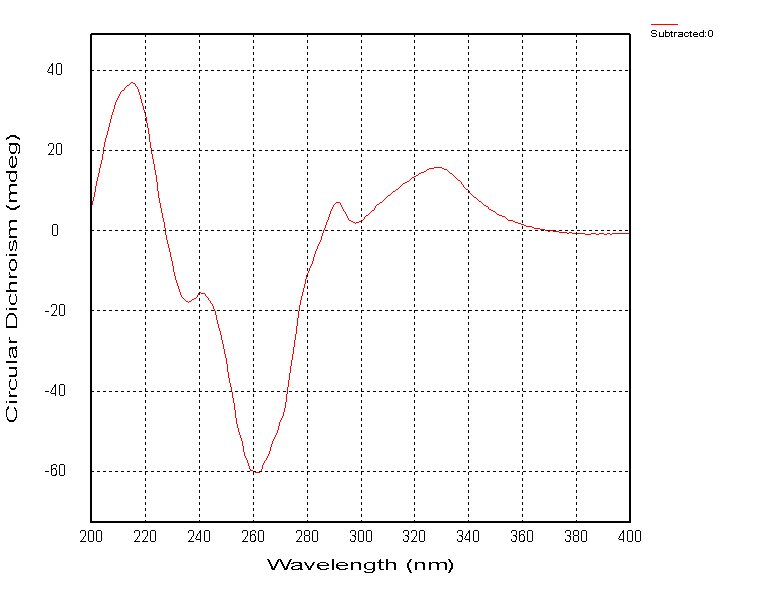


**Figure S1.8 CD spectrum of 1**

**
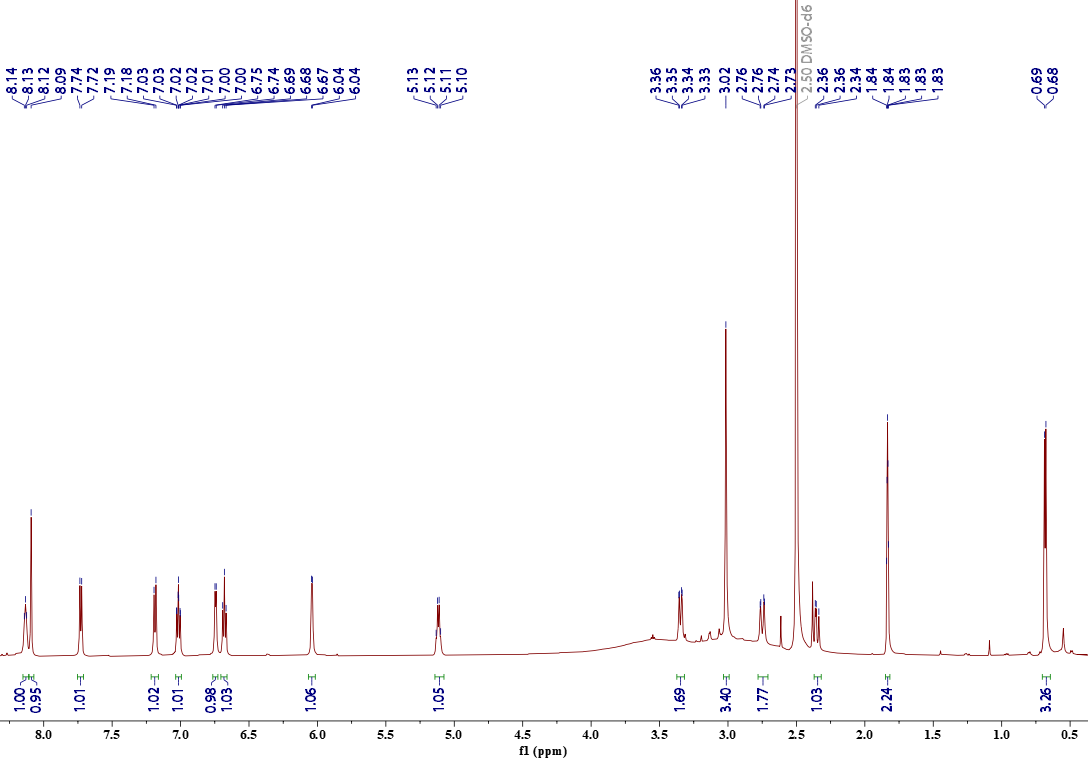
**

**Figure S2.1 ^1^H NMR spectrum of 2.**


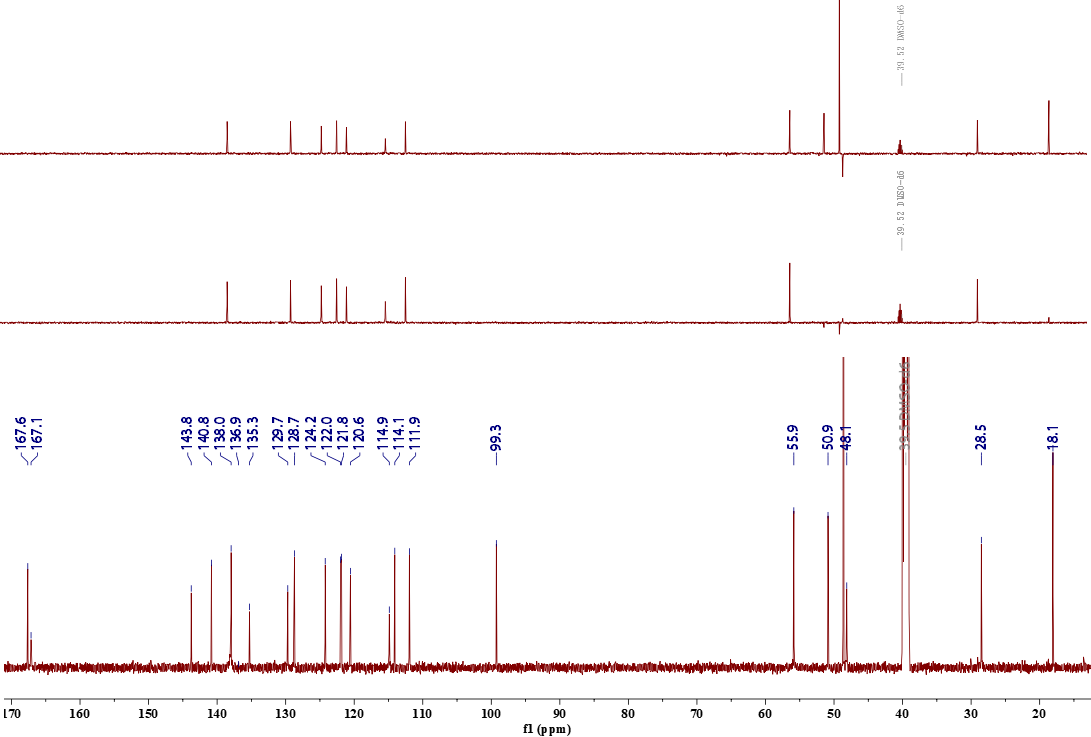


**Figure S2.2 ^13^C NMR spectrum of 2.**


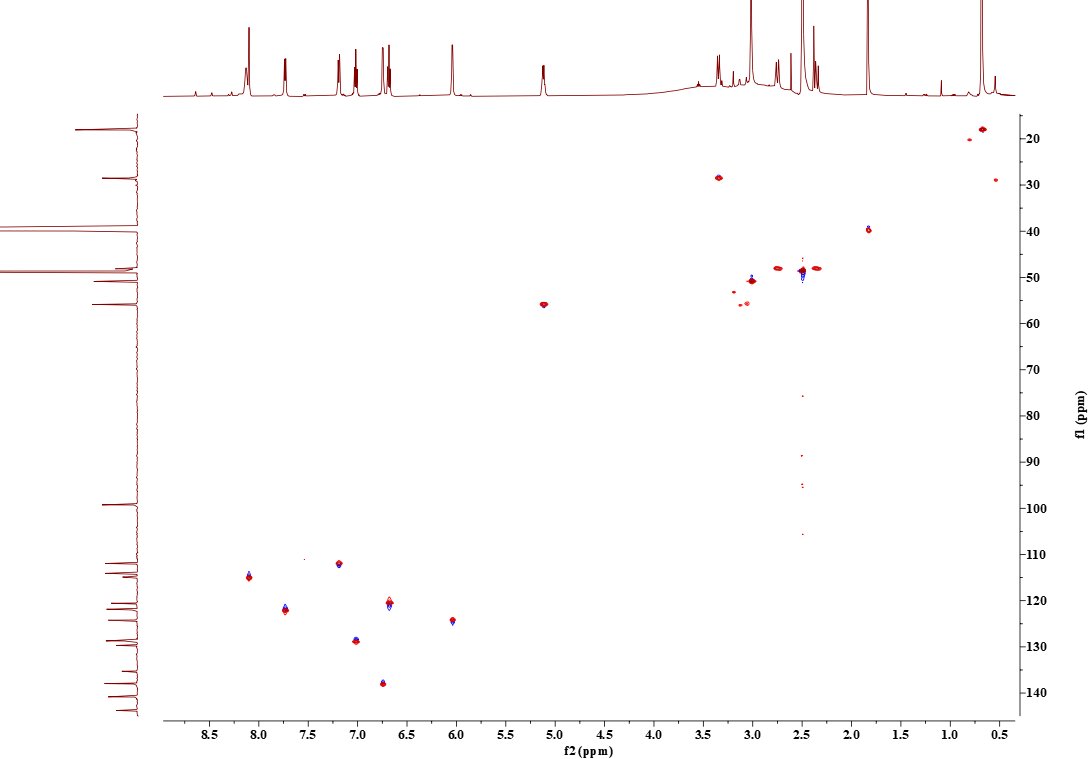


**Figure S2.3 HSQC spectrum of 2**


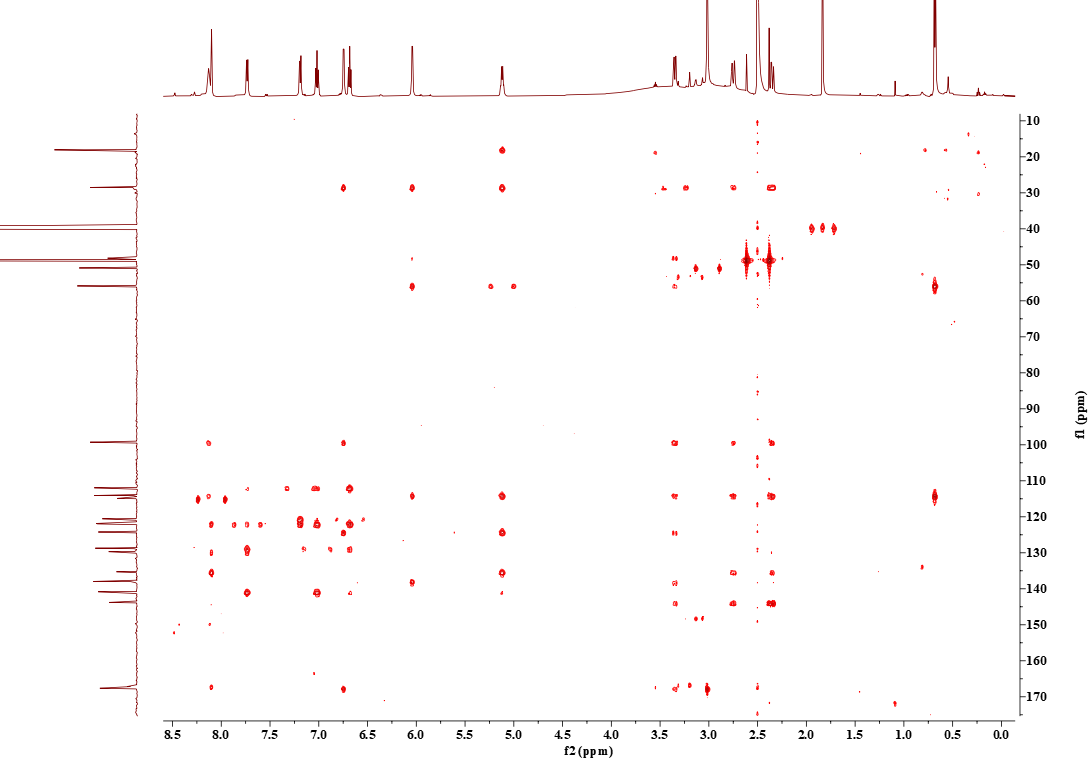


**Figure S2.4 HMBC spectrum of 2**


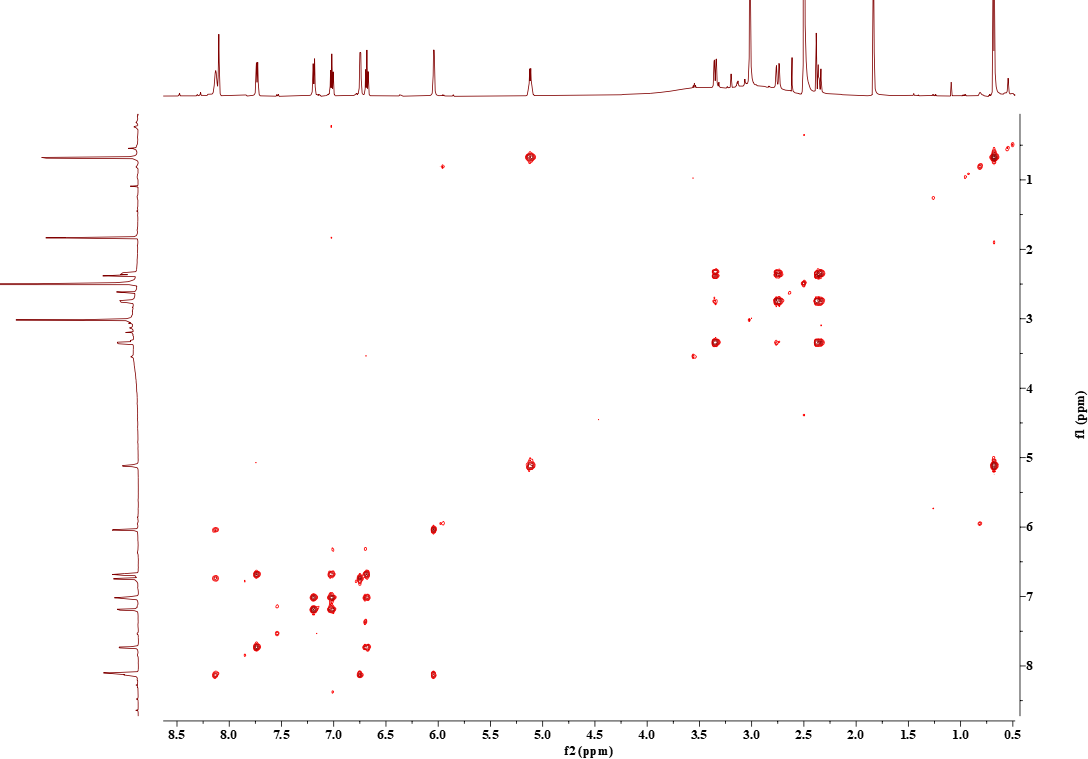


**Figure S2.5 COSY spectrum of 2.**


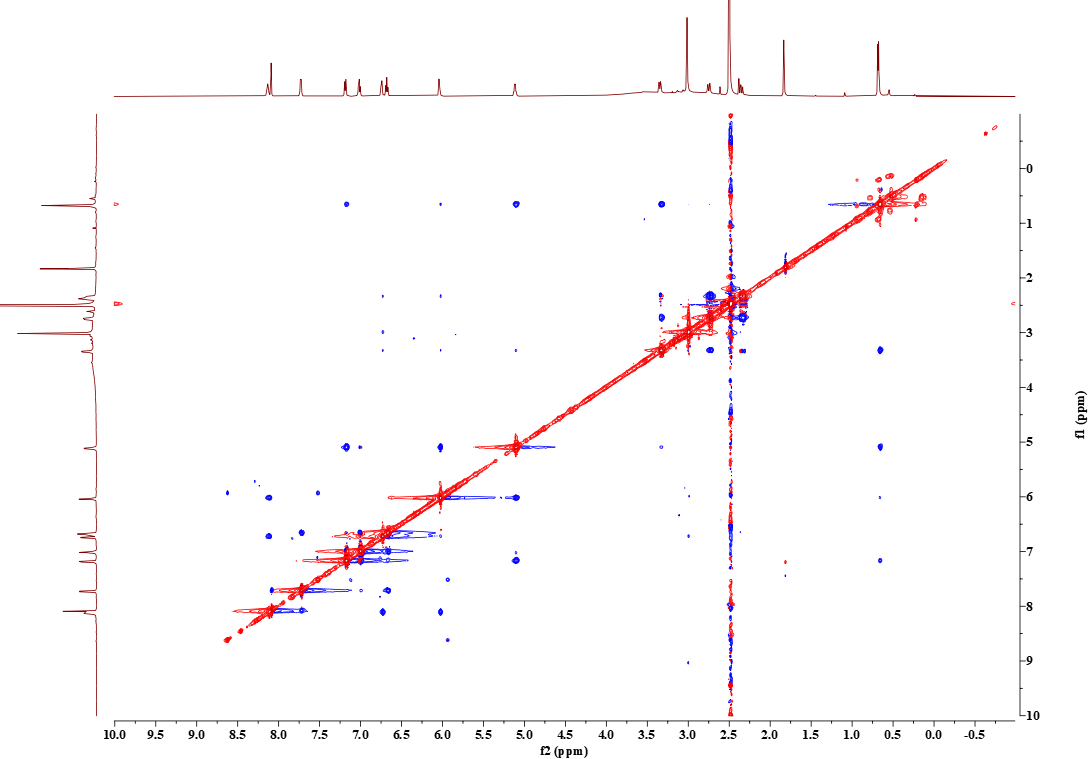


**Figure S2.6 ROESY spectrum of 2**

**Figure S2.7 HRMS spectrum of 2**

**
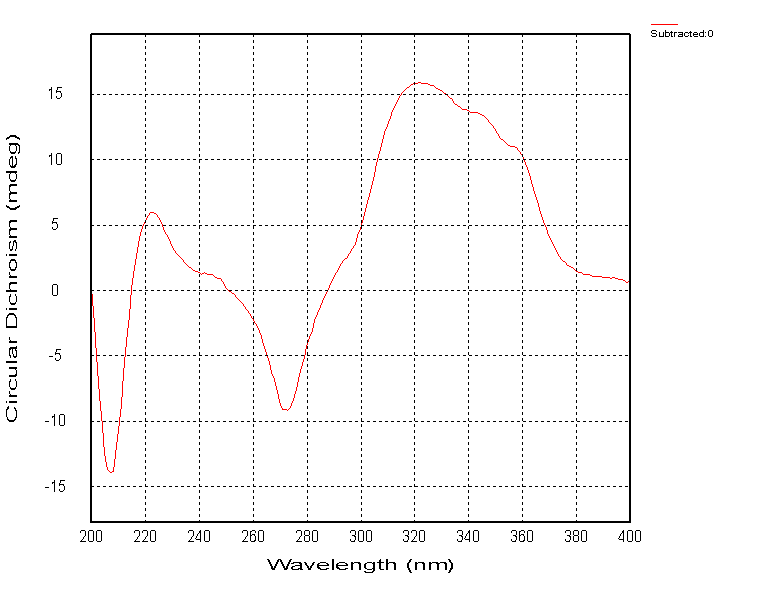
**

**Figure S2.8 CD spectrum of 2**


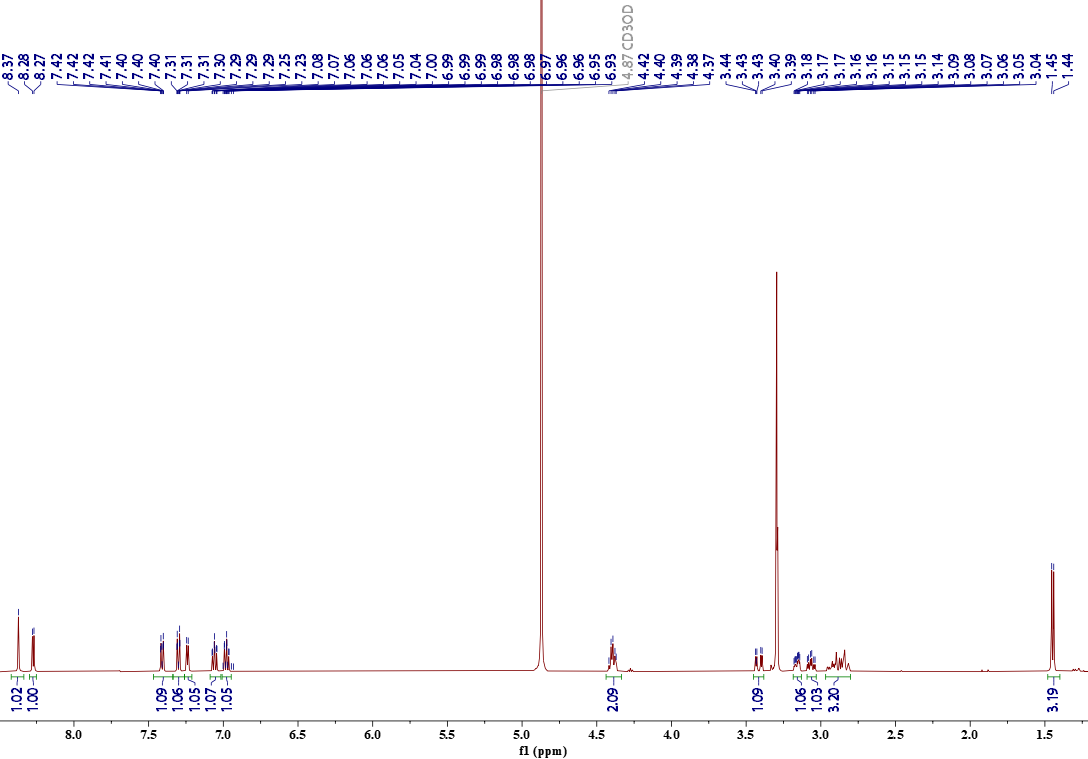


**Figure S3.1 ^1^H NMR spectrum of 3**


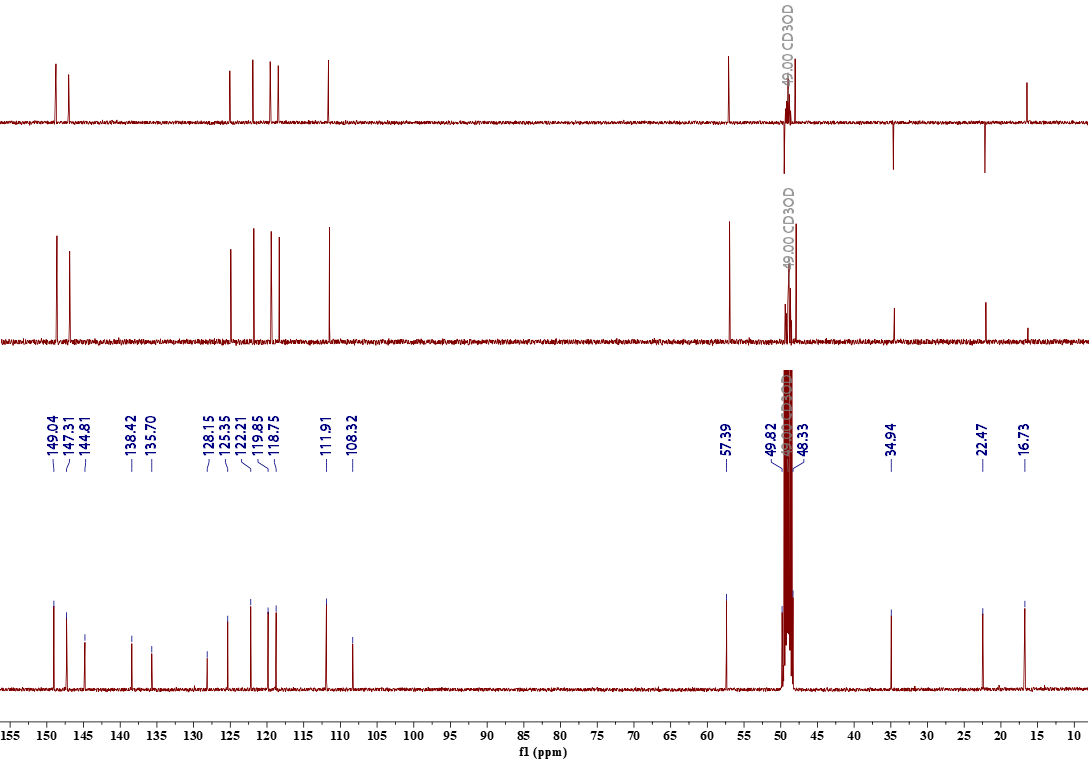


**Figure S3.2 ^13^C NMR spectrum of 3**


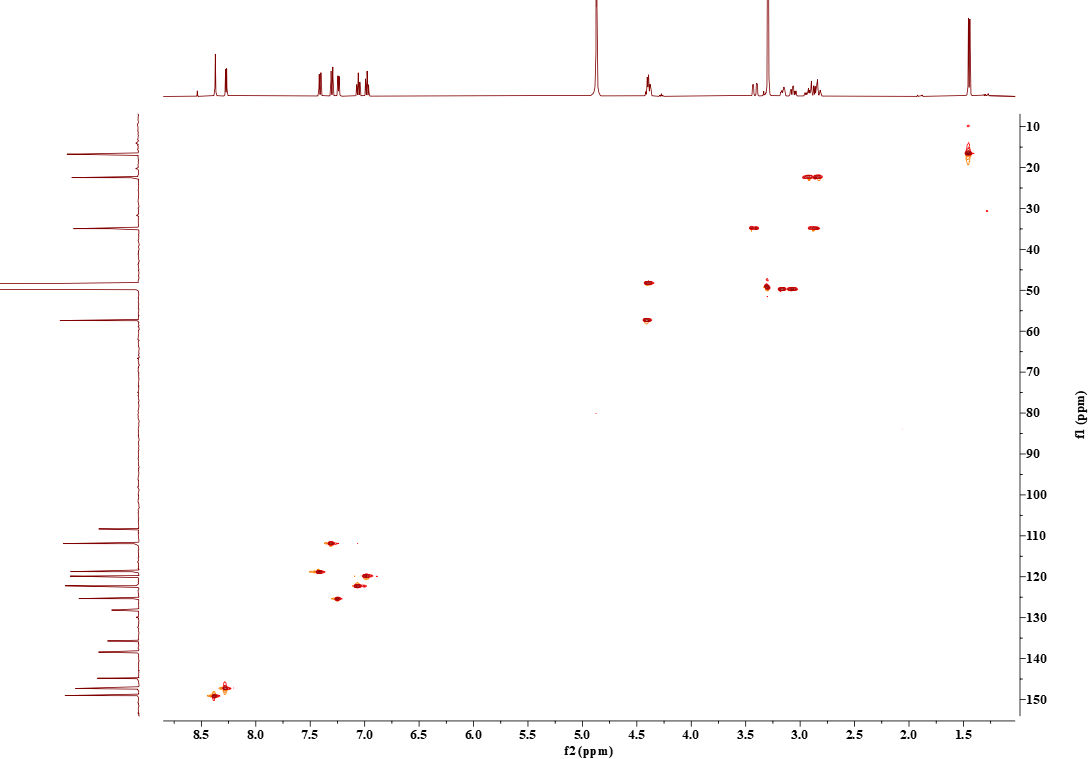


**Figure S3.3 HSQC spectrum of 3**


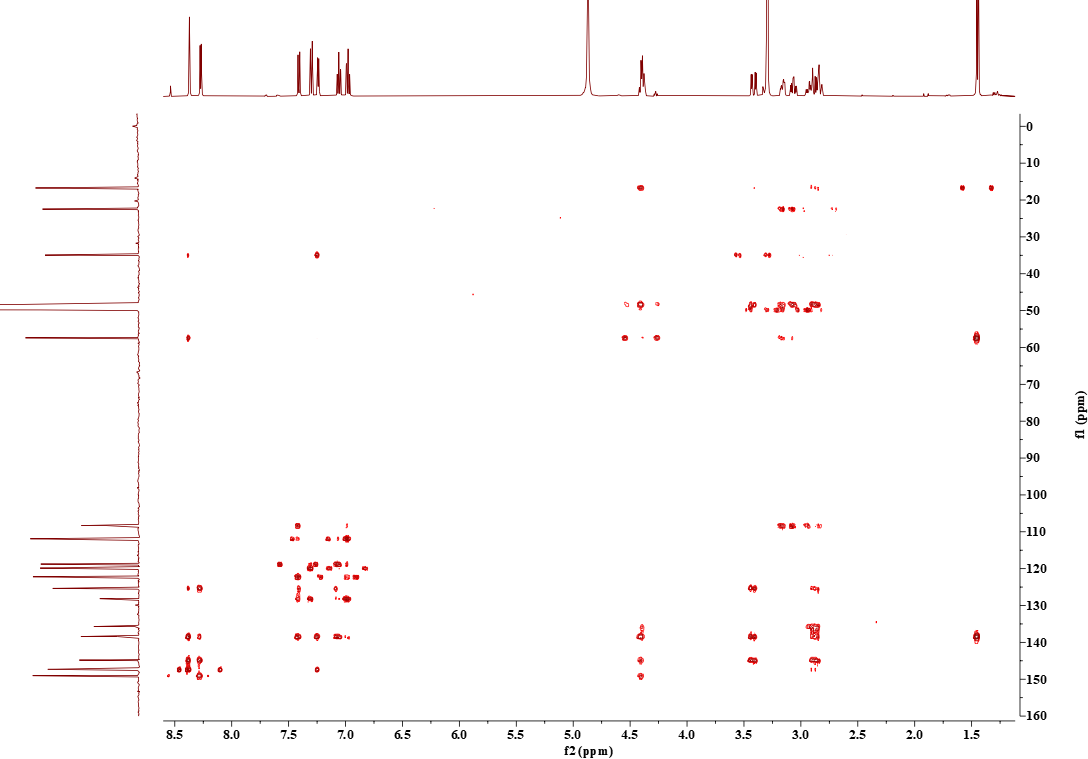


**Figure S3.4 HMBC spectrum of 3**


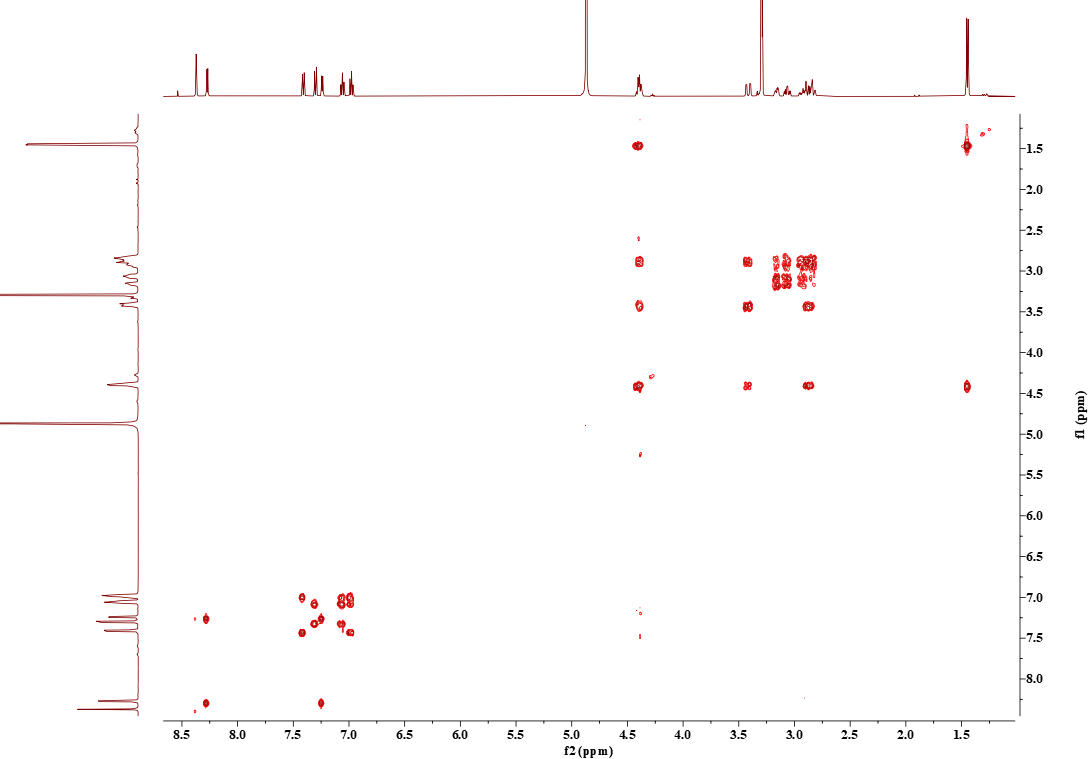


**Figure S3.5 COSY spectrum of 3**


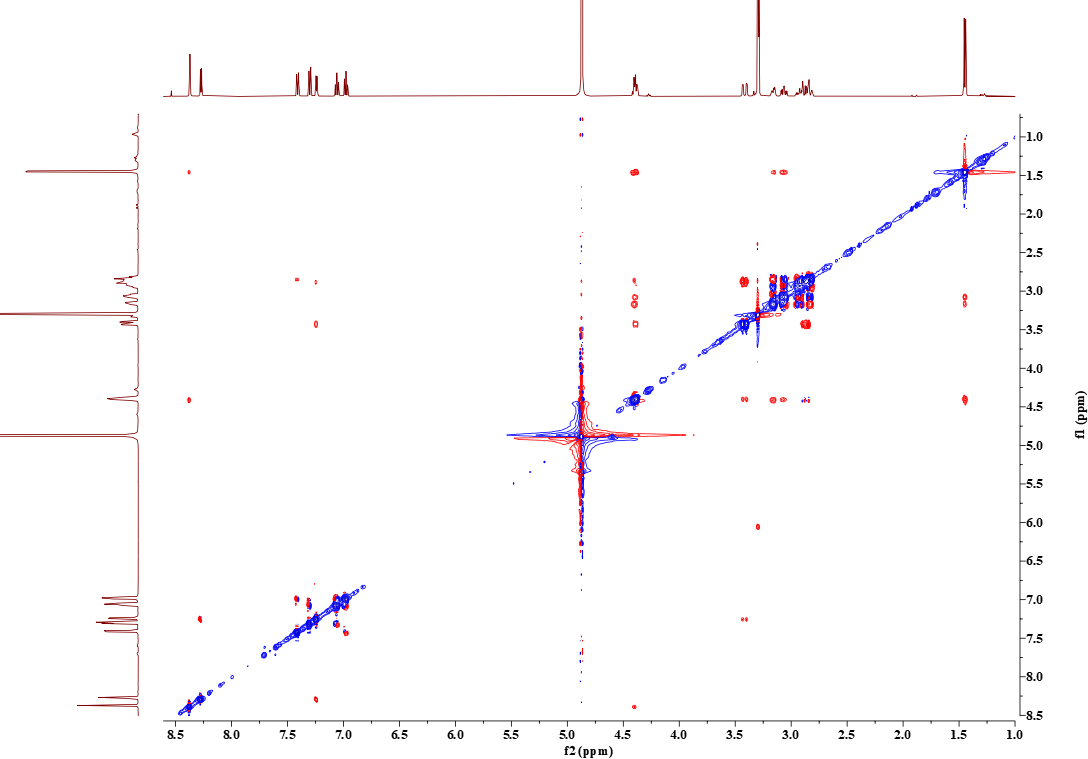


**Figure S3.6 ROESY spectrum of 3**

**Figure S3.7 HRMS spectrum of 3**


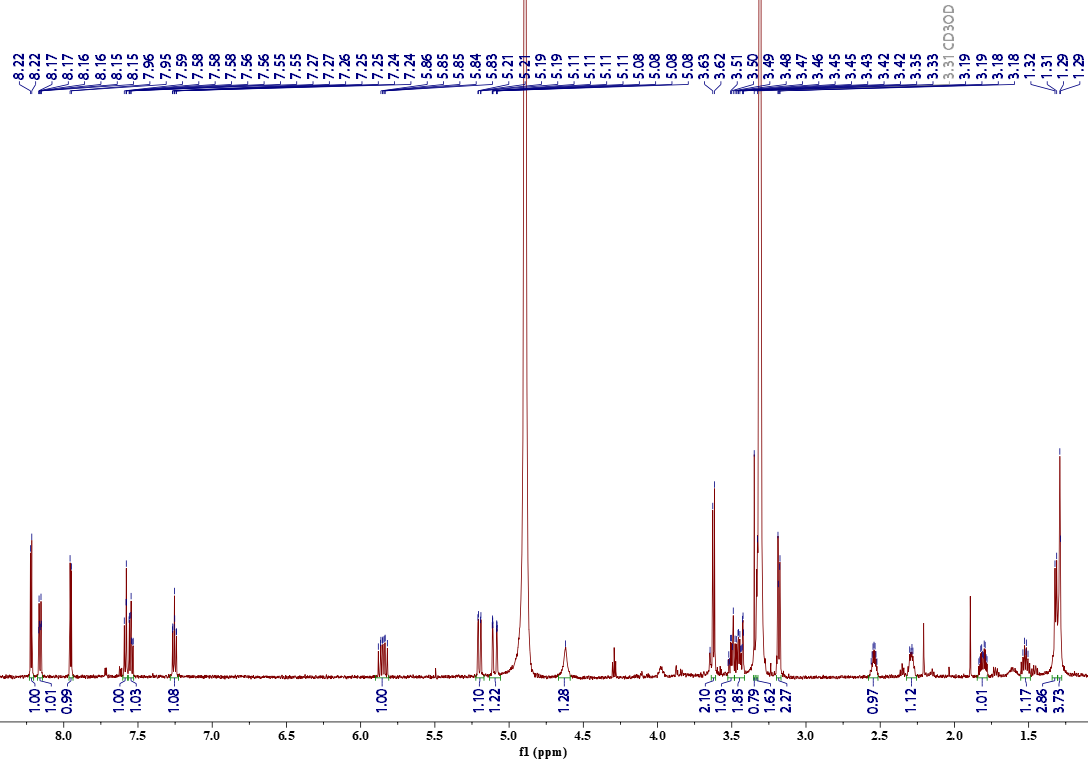


**Figure S4.1 ^1^H NMR spectrum of 4**


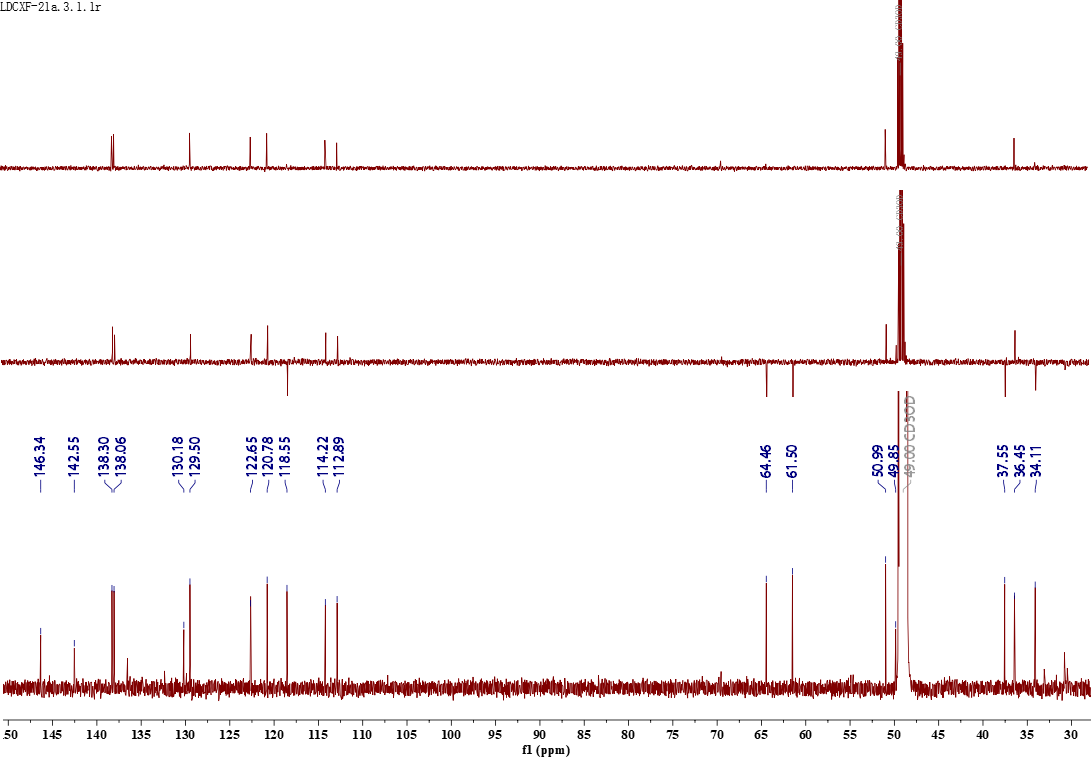


**Figure S4.2 ^13^C NMR spectrum of 4**


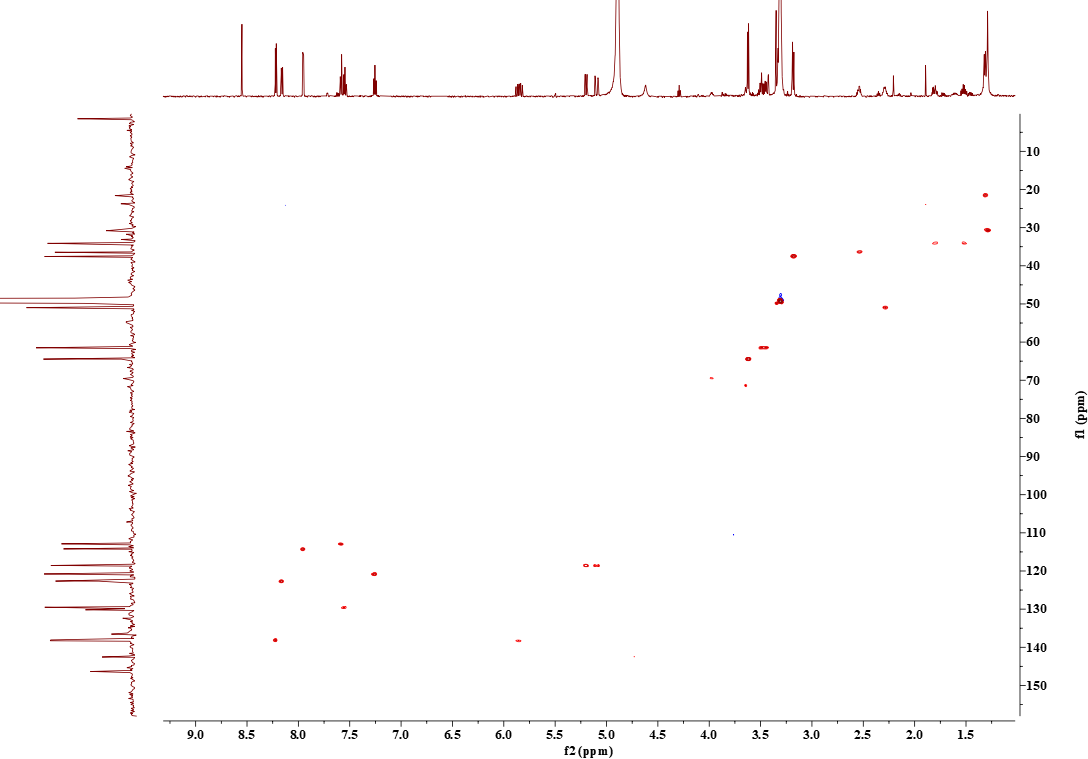


**Figure S4.3 HSQC spectrum of 4**


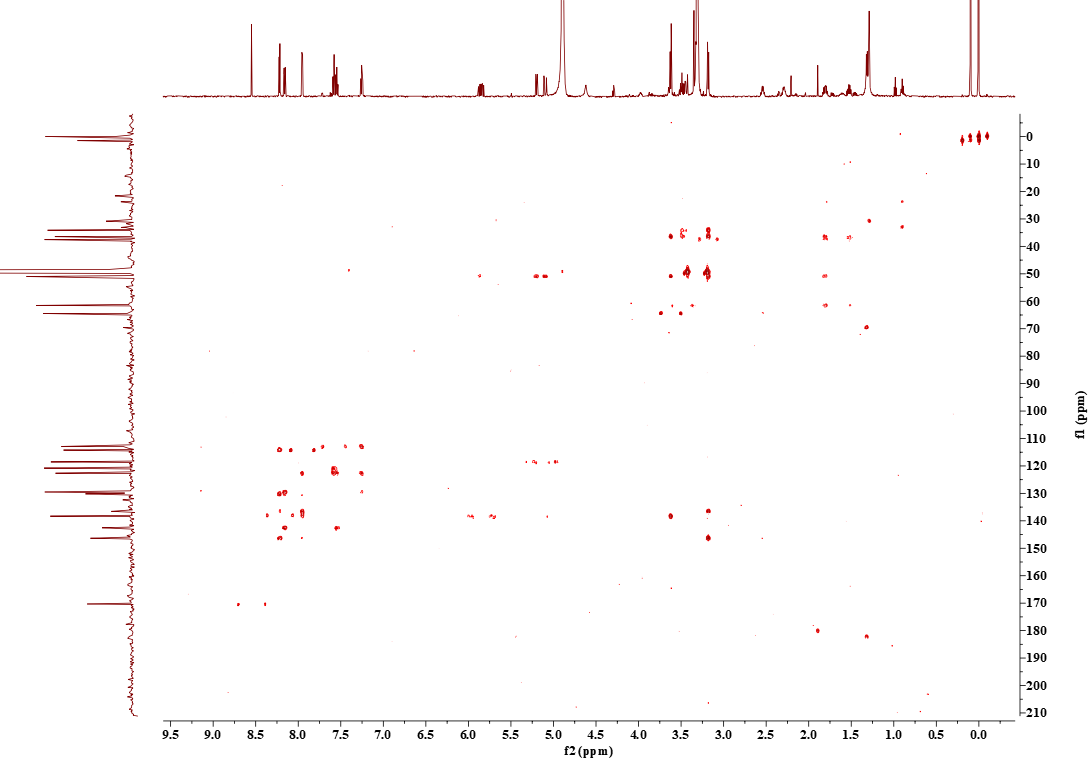


**Figure S4.4 HMBC spectrum of 4**


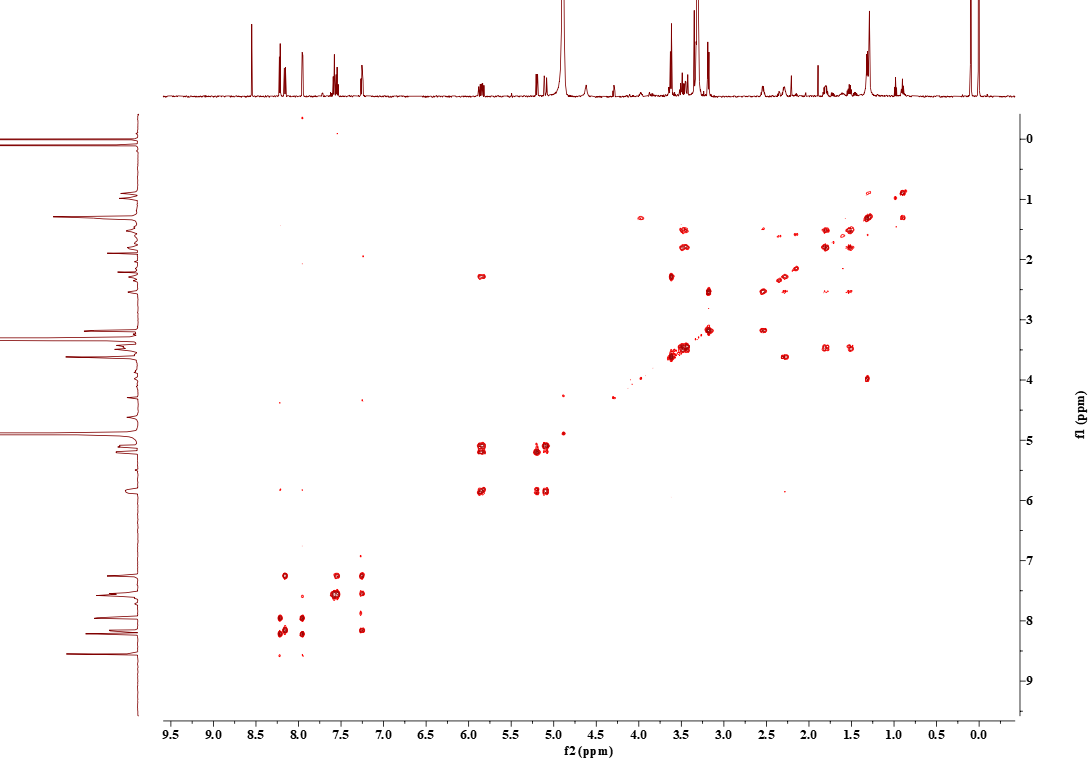


**Figure S4.5 COSY spectrum of 4**


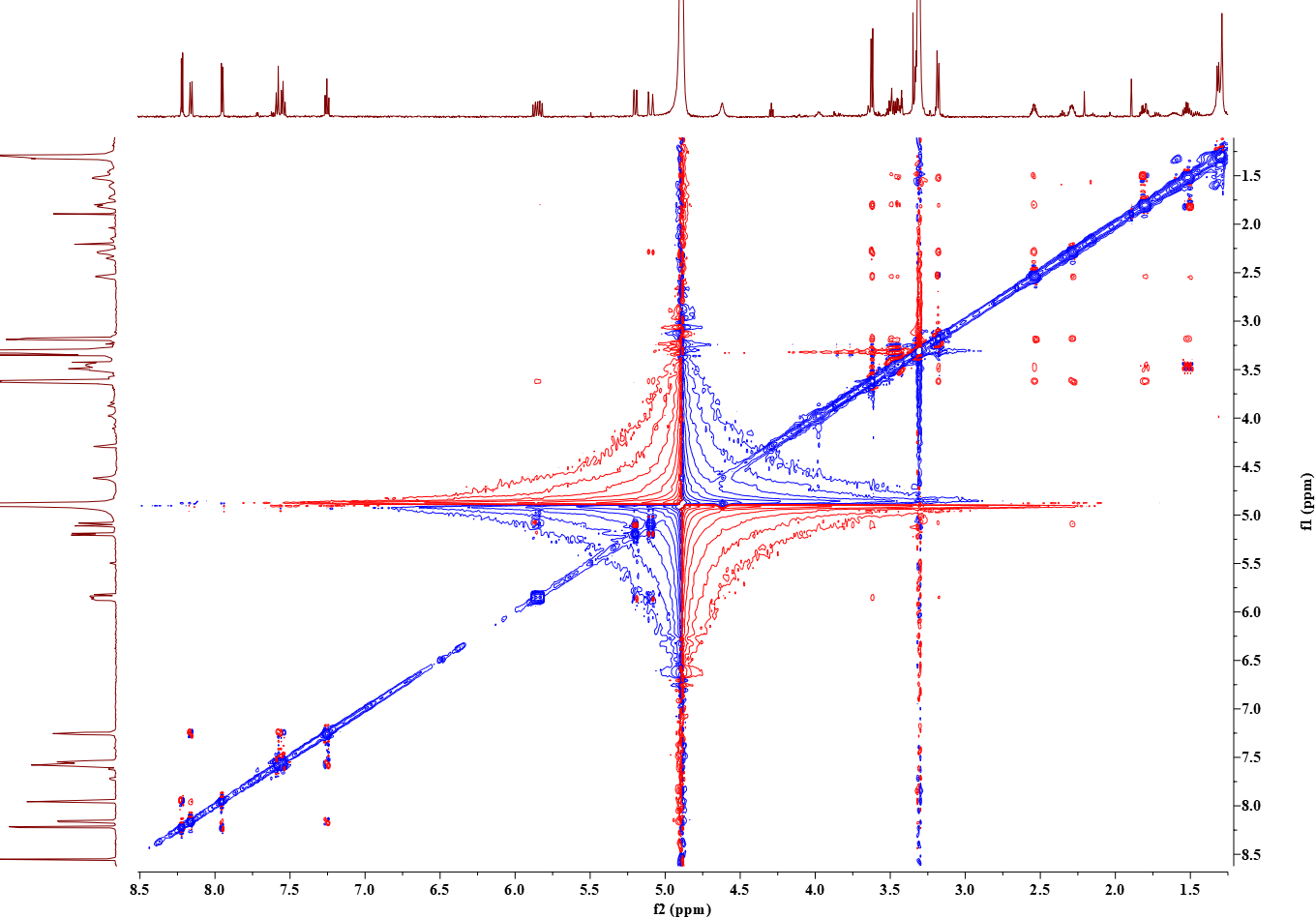


**Figure S4.6 ROESY spectrum of 4**

**Figure S4.7 HRMS spectrum of 4**


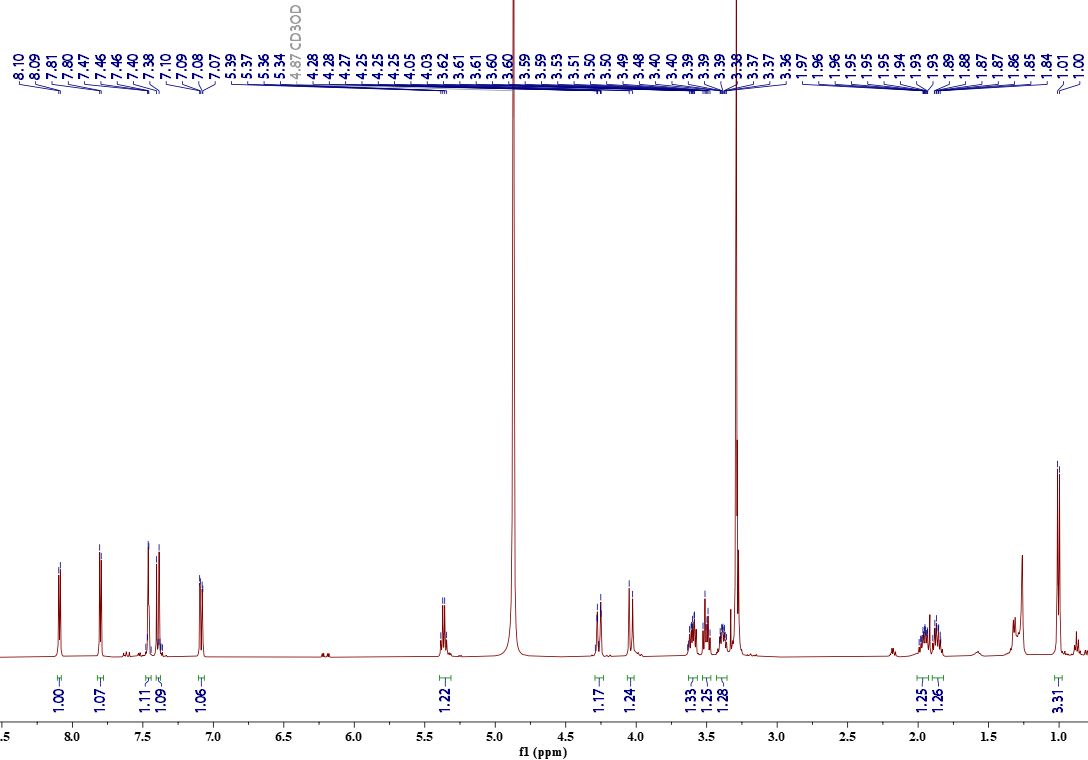


**Figure S5.1 ^1^H NMR spectrum of 5**


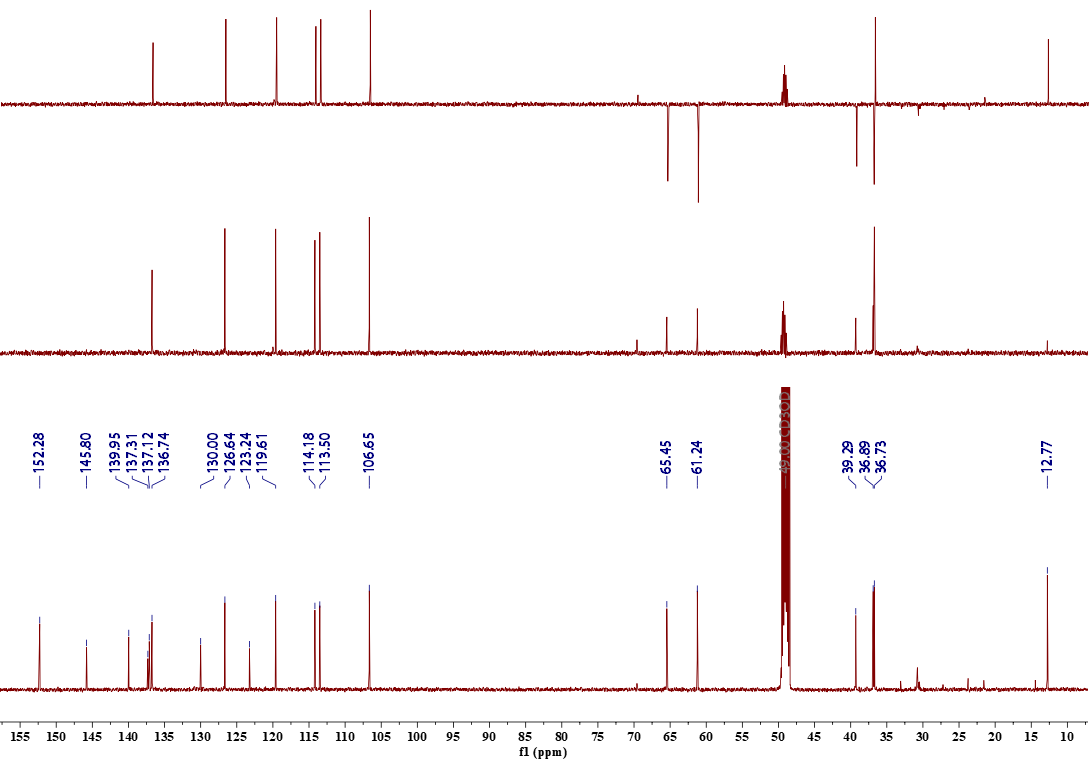


**Figure S5.2 ^13^C NMR spectrum of 5**


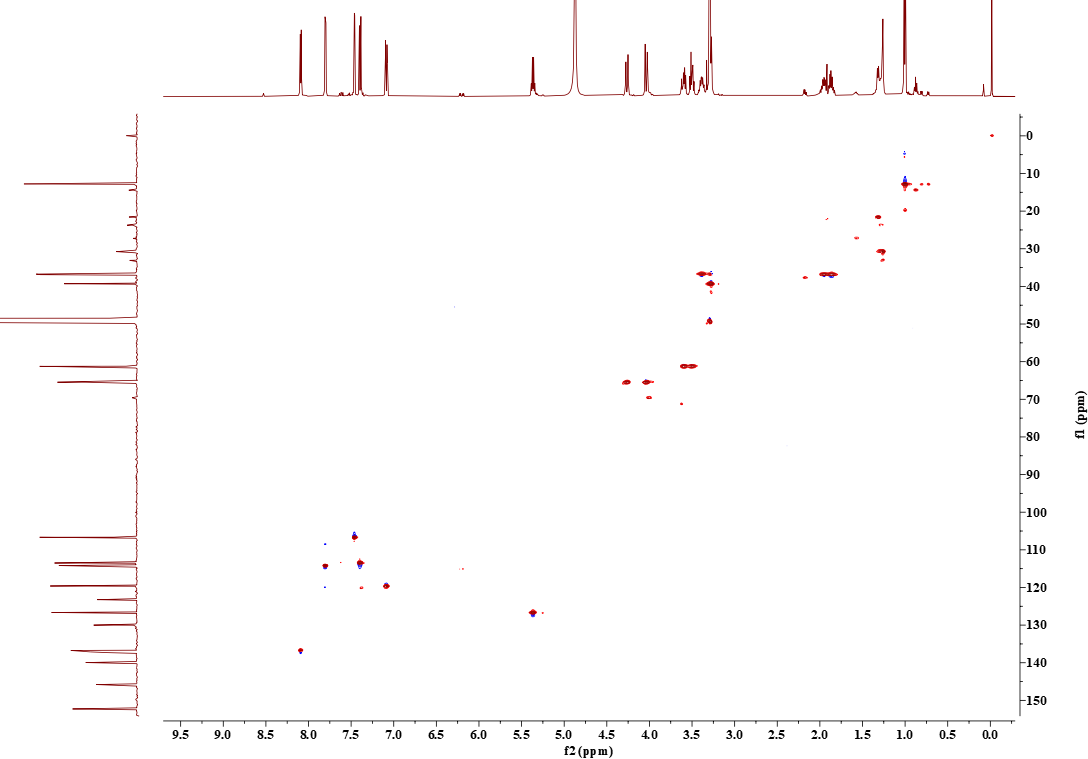


**Figure S5.3 HSQC spectrum of 5**


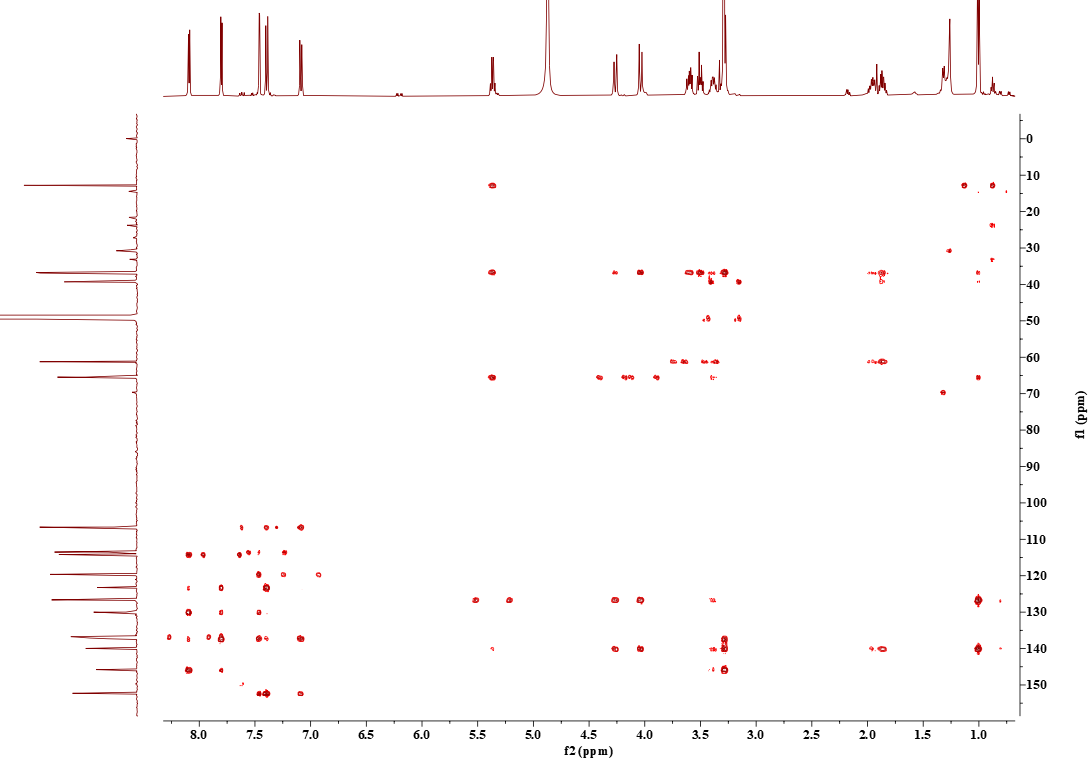


**Figure S5.4 HMBC spectrum of 5**


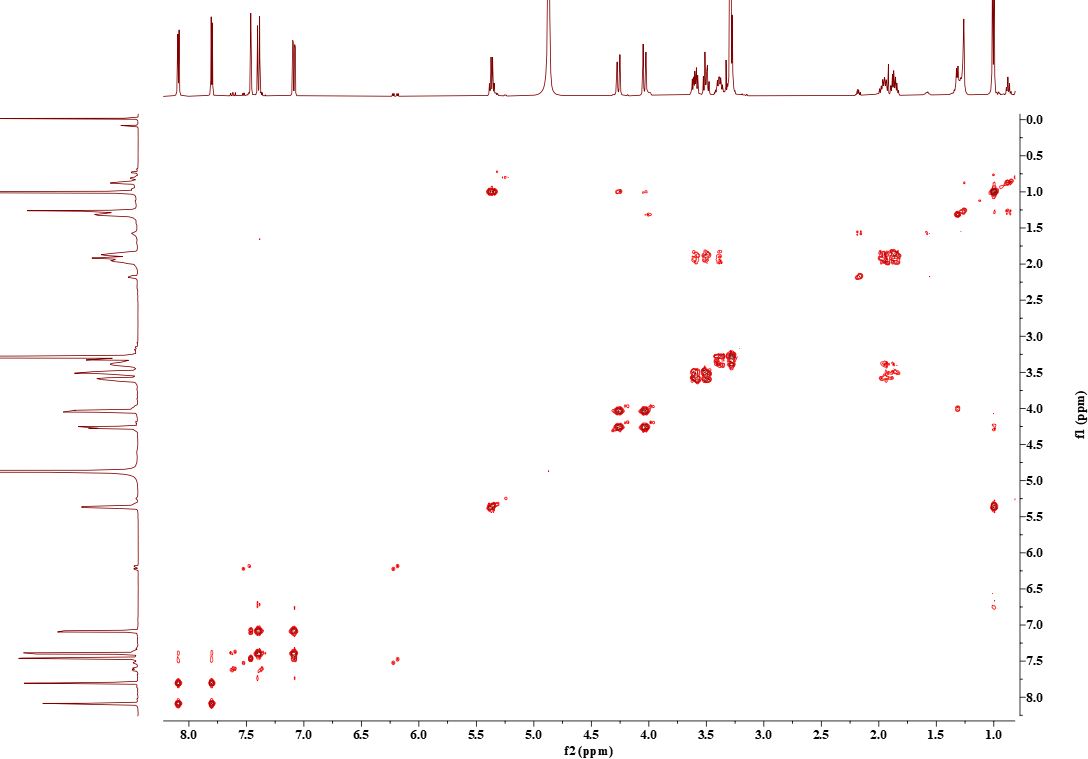


**Figure S5.5 COSY spectrum of 5**


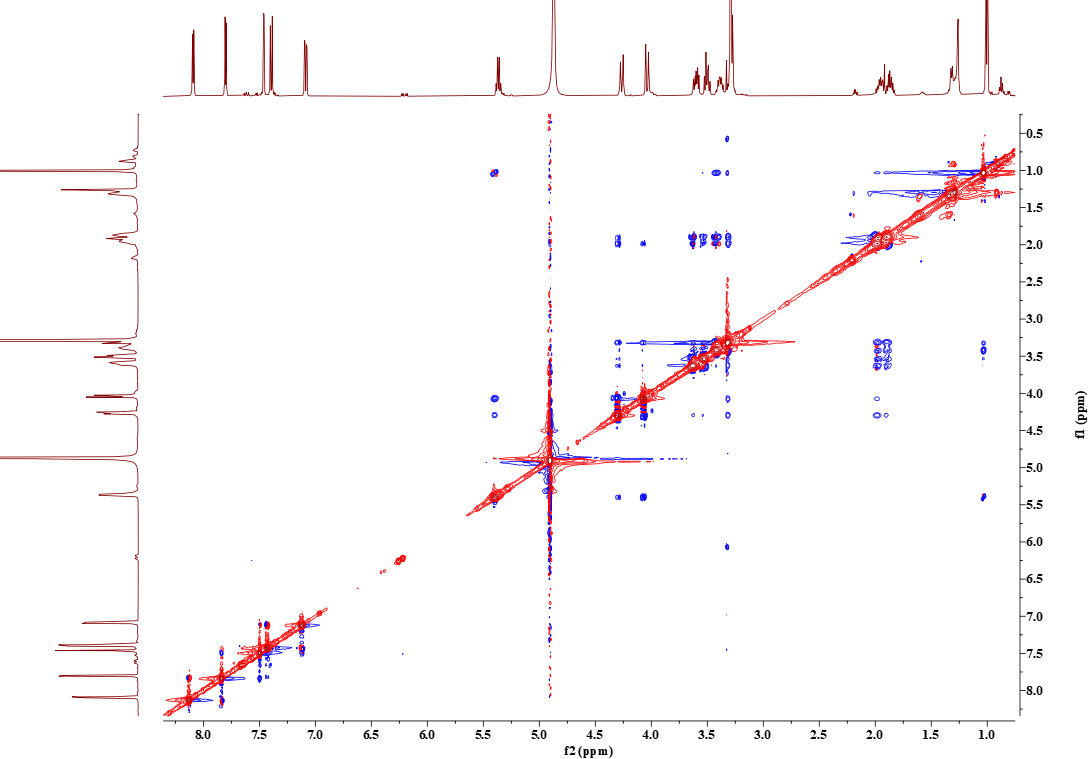


**Figure S5.6 ROESY spectrum of 5**

**Figure S5.7 HRMS spectrum of 5**


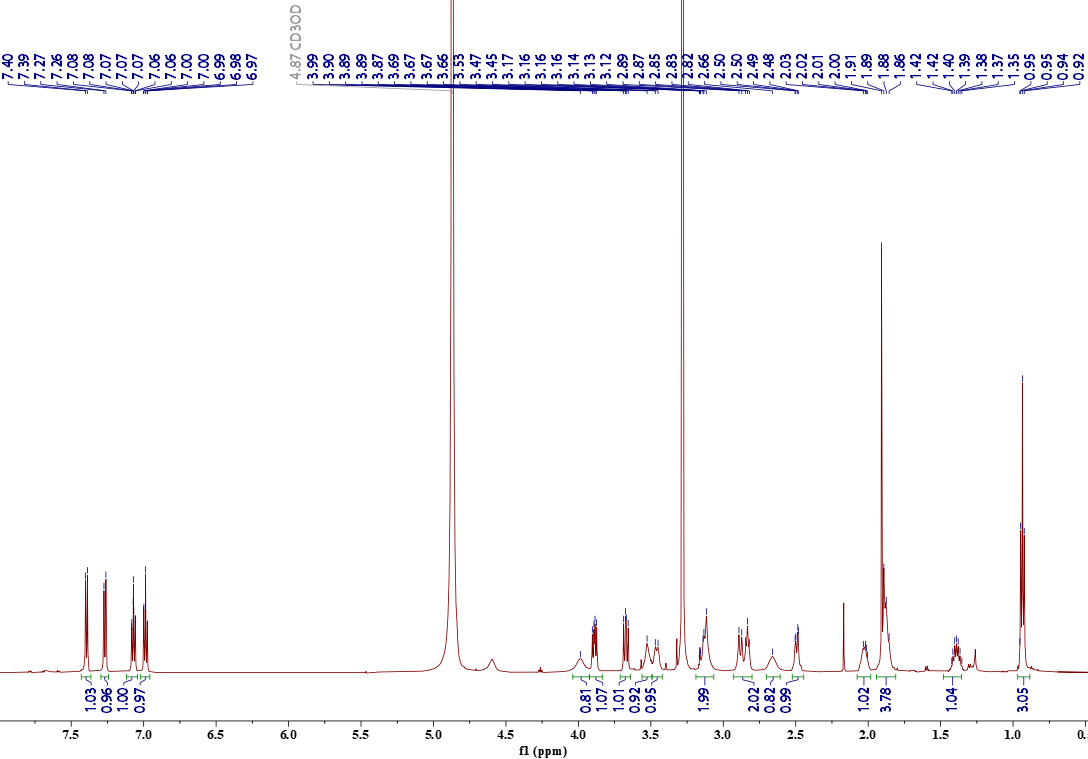


**Figure S6.1 ^1^H NMR spectrum of 6**


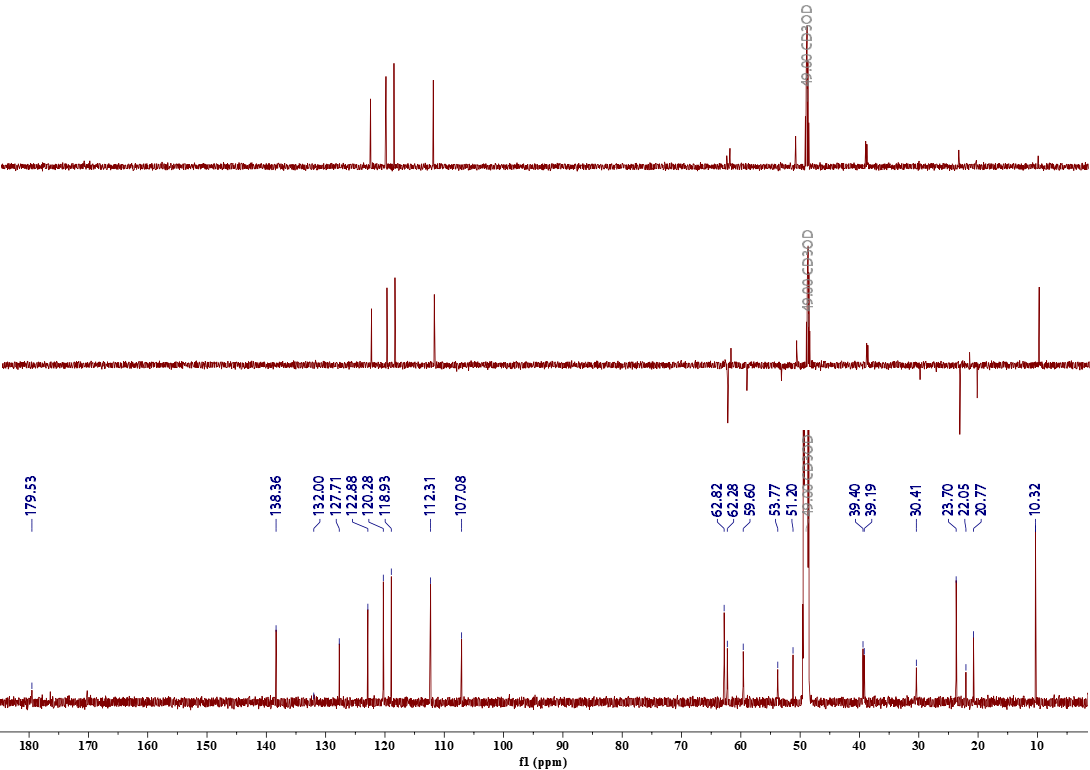


**Figure S6.2 ^13^C NMR spectrum of 6**


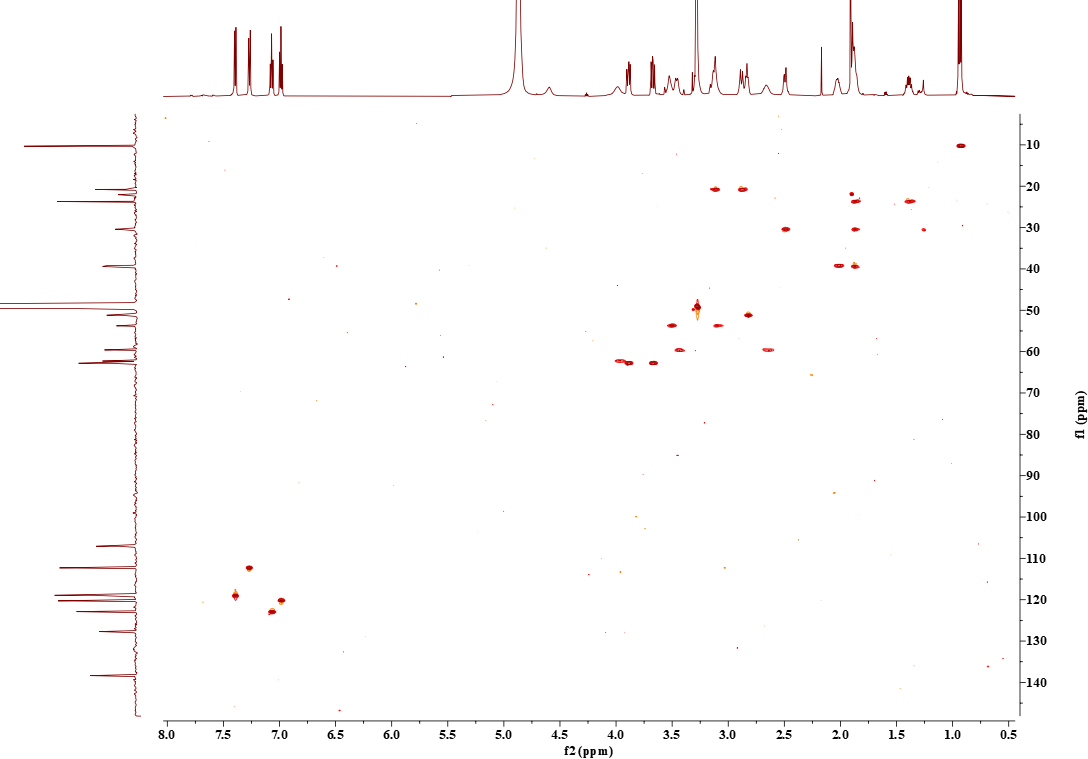


**Figure S6.3 HSQC spectrum of 6**


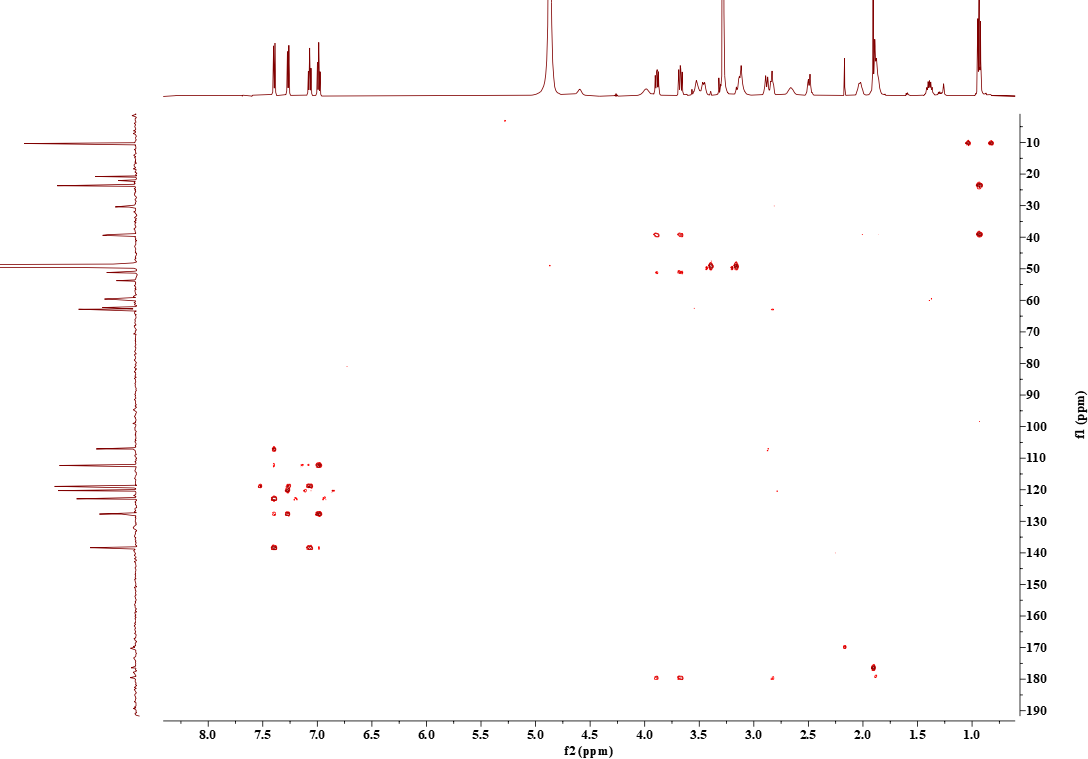


**Figure S6.4 HMBC spectrum of 6**


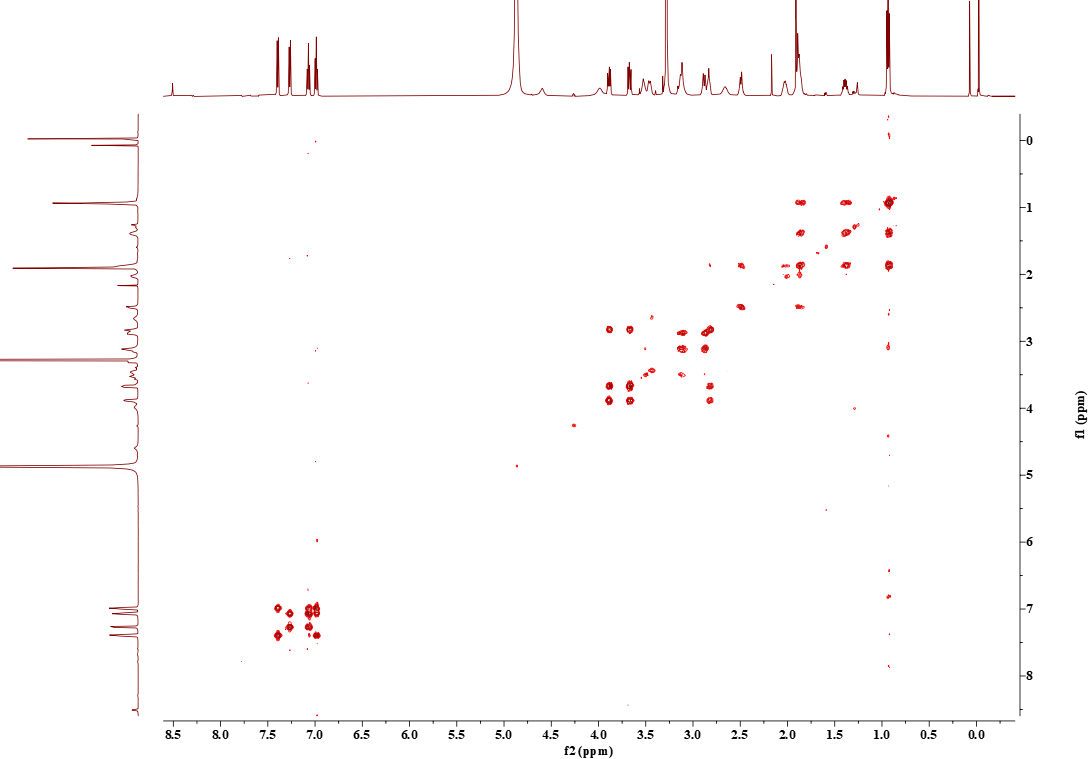


**Figure S6.5 COSY spectrum of 6**


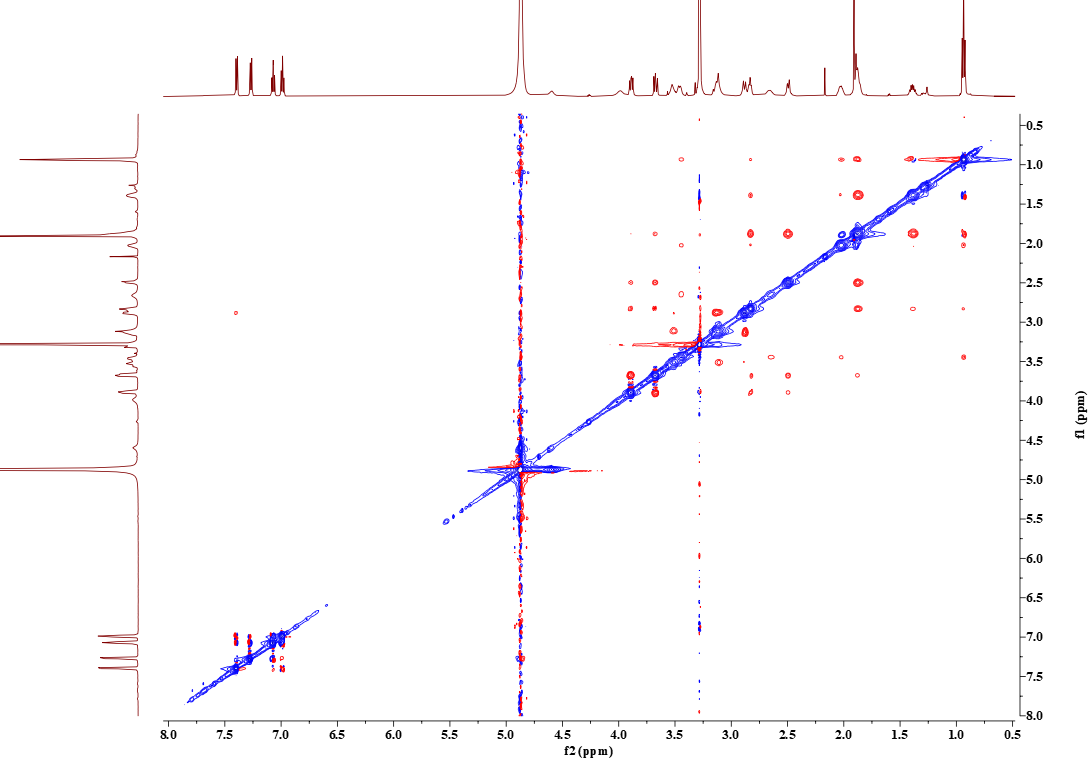


**Figure S6.6 ROESY spectrum of 6**

**Figure S6.7 HRMS spectrum of 6.**


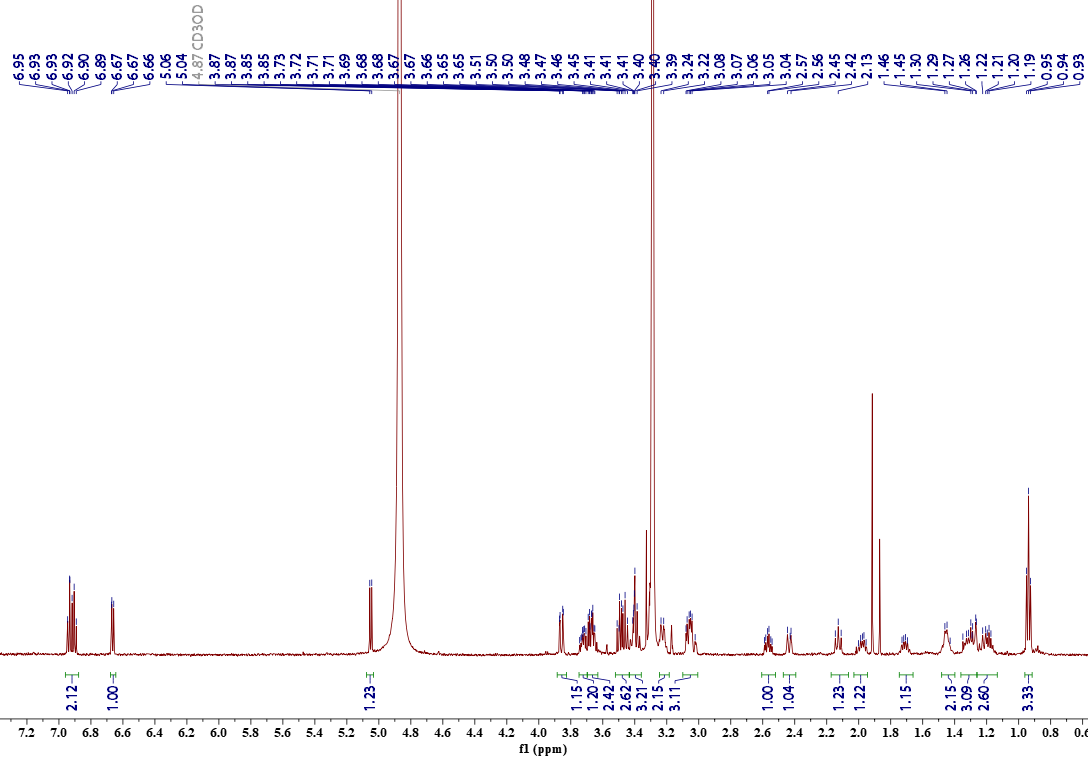


**Figure S7.1 ^1^H NMR spectrum of 7**


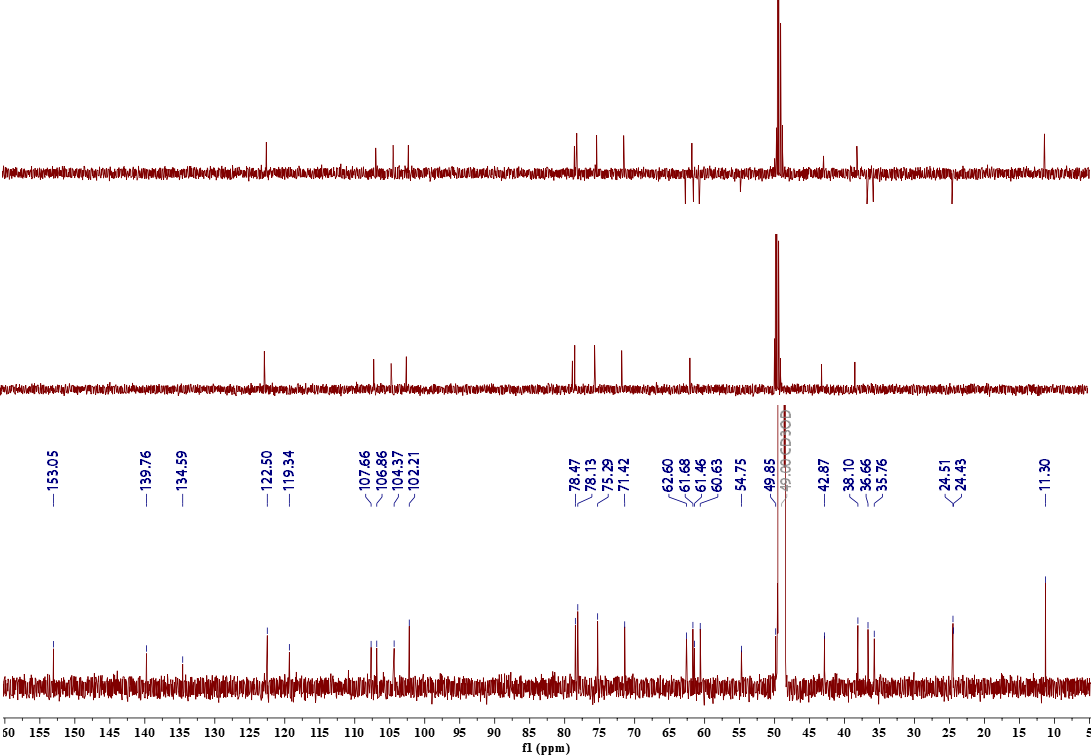


**Figure S7.2 ^13^C NMR spectrum of 7**


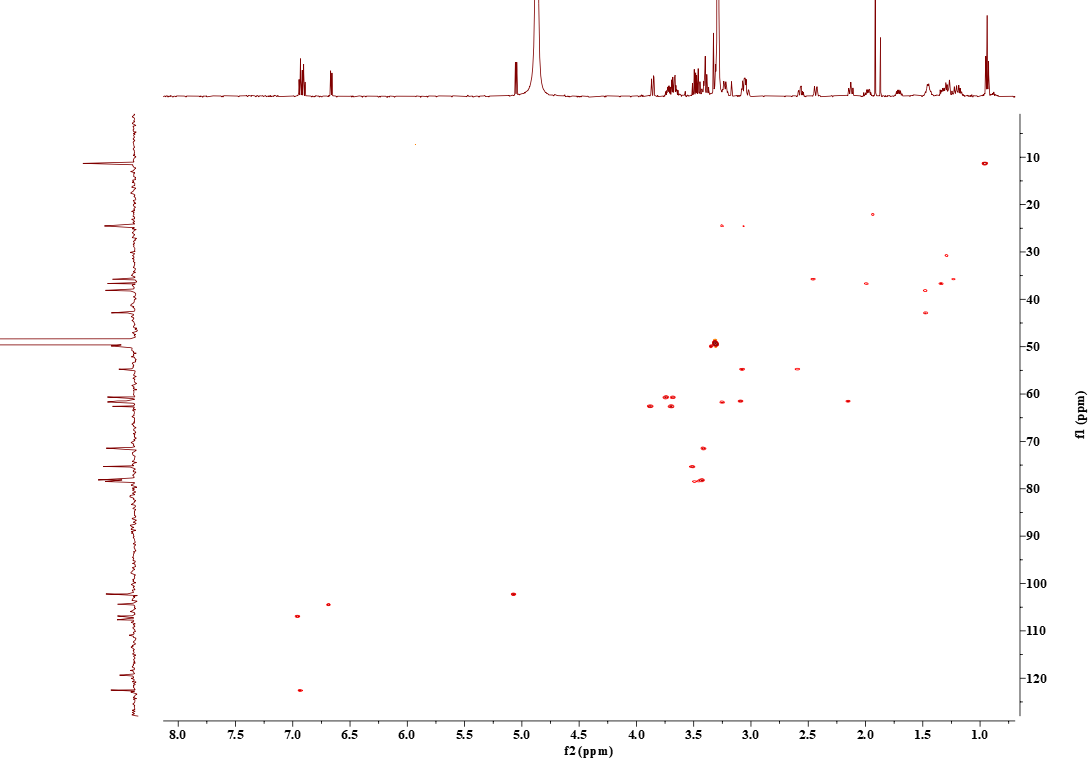


**Figure S7.3 HSQC spectrum of 7**


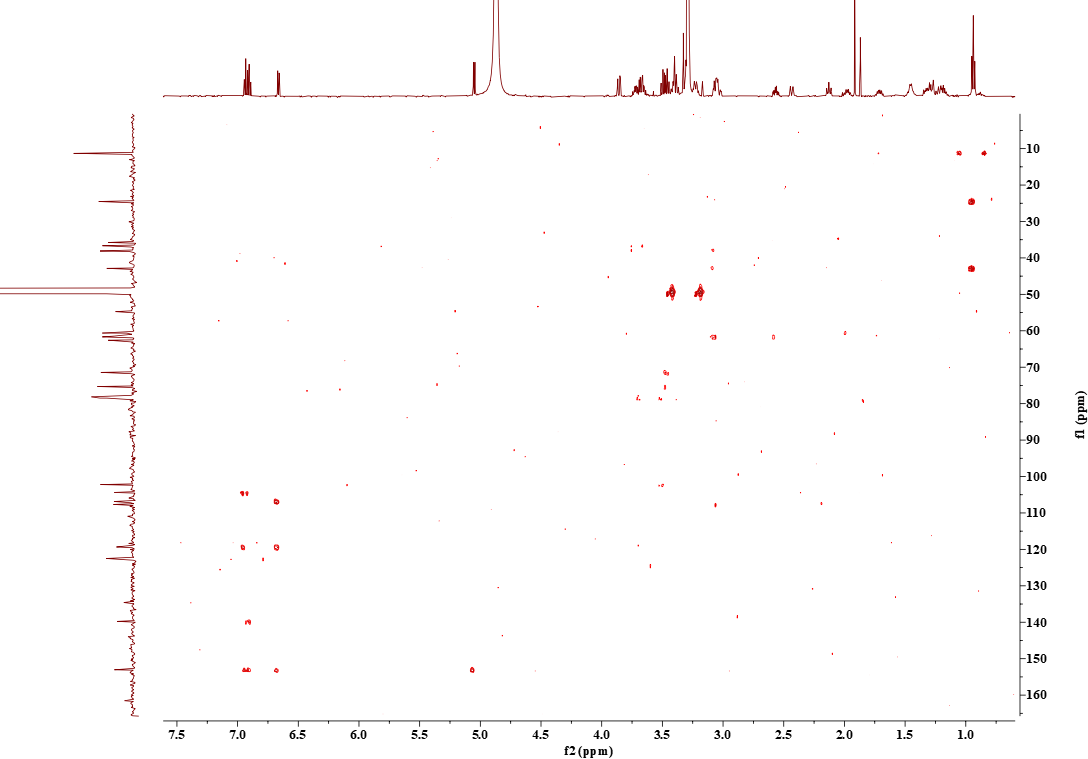


**Figure S7.4 HMBC spectrum of 7**


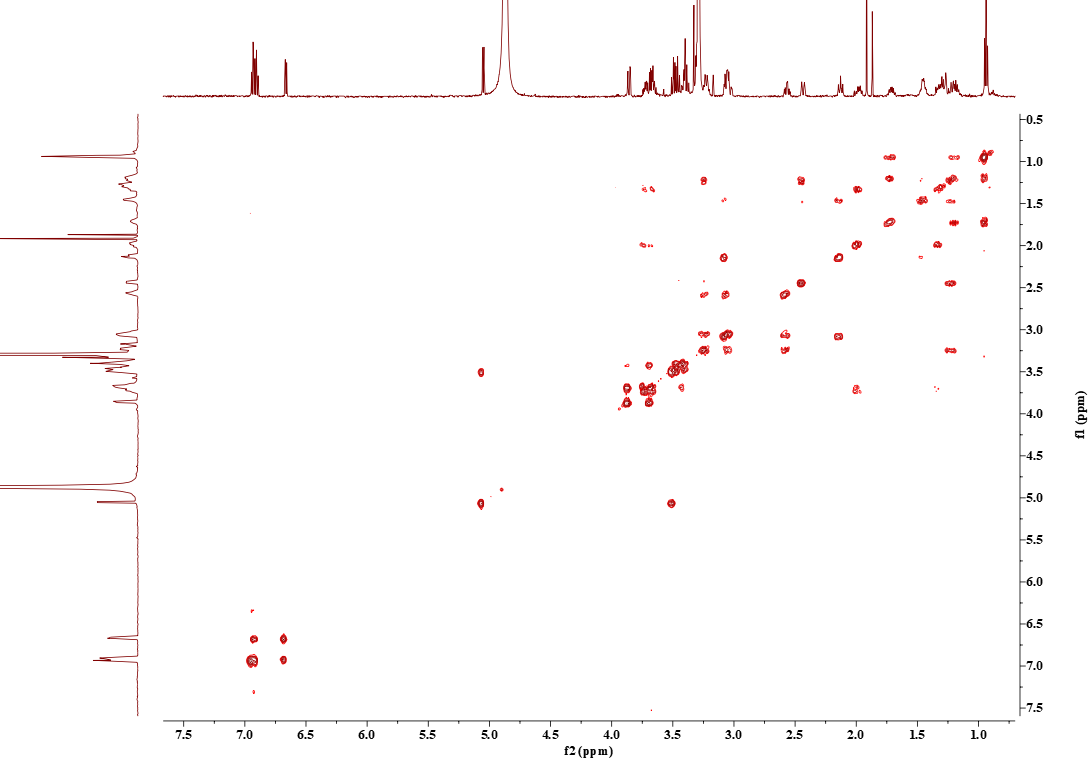


**Figure S7.5 COSY spectrum of 7**


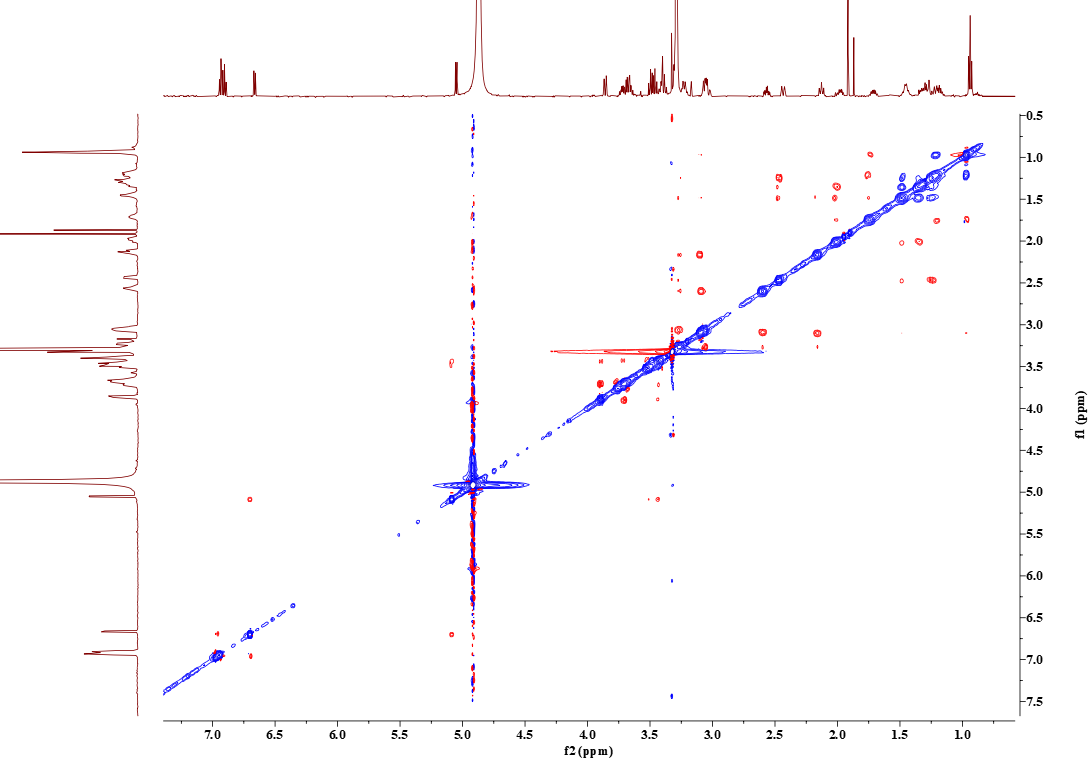


**Figure S7.6 ROESY spectrum of 7**

**Figure S7.7 HRMS spectrum of 7**


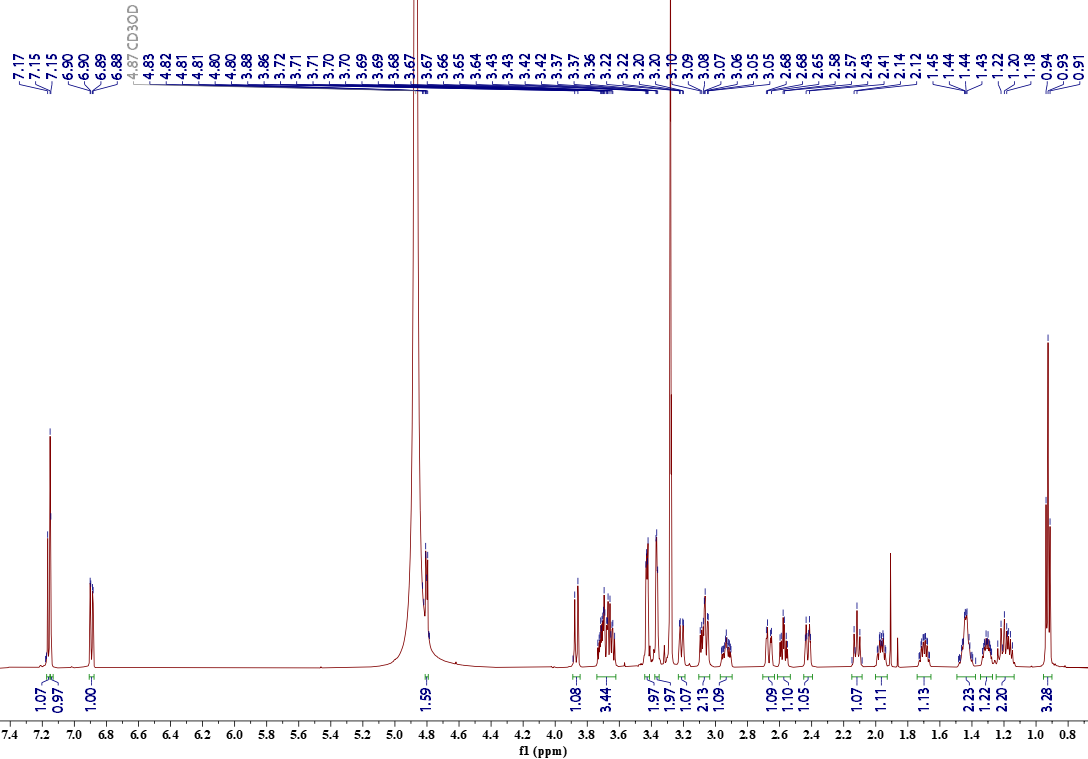


**Figure S8.1 ^1^H NMR spectrum of 8**


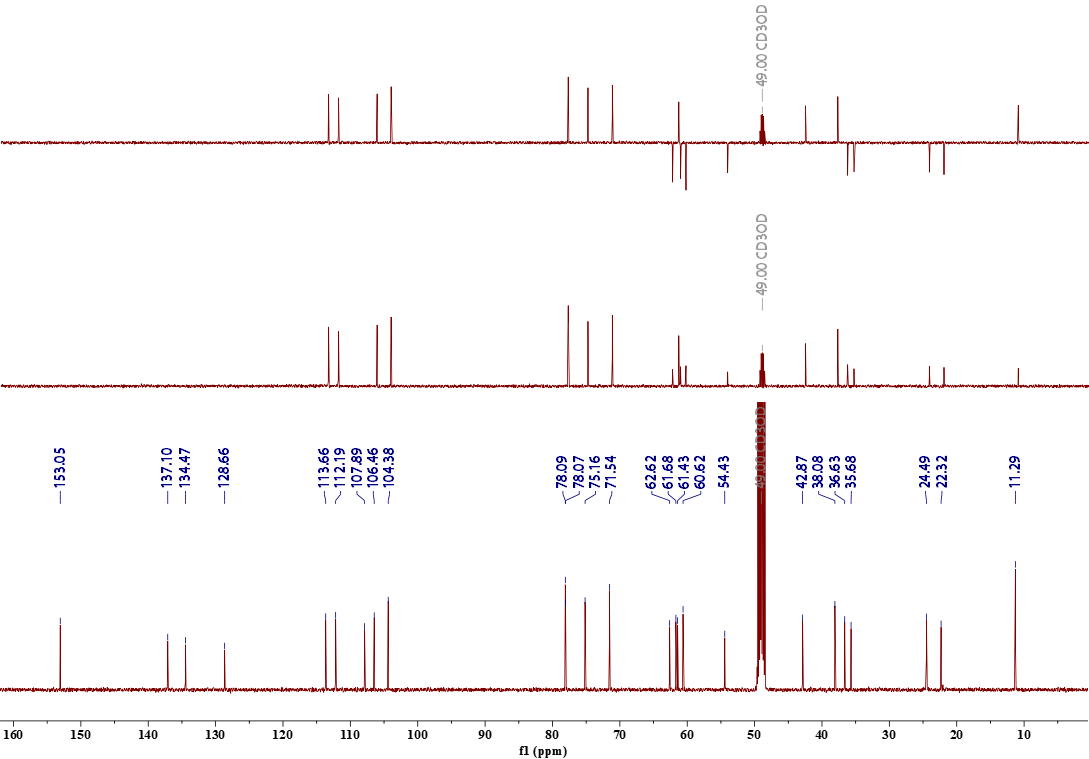


**Figure S8.2 ^13^C NMR spectrum of 8**


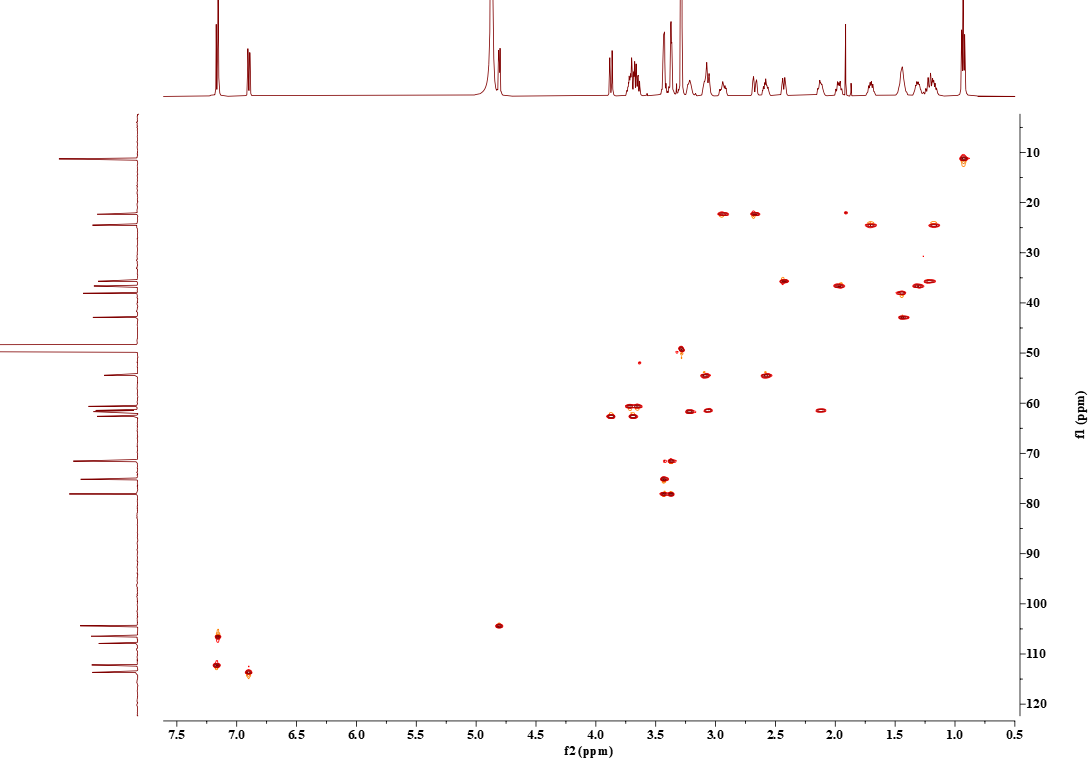


**Figure S8.3 HSQC spectrum of 8**


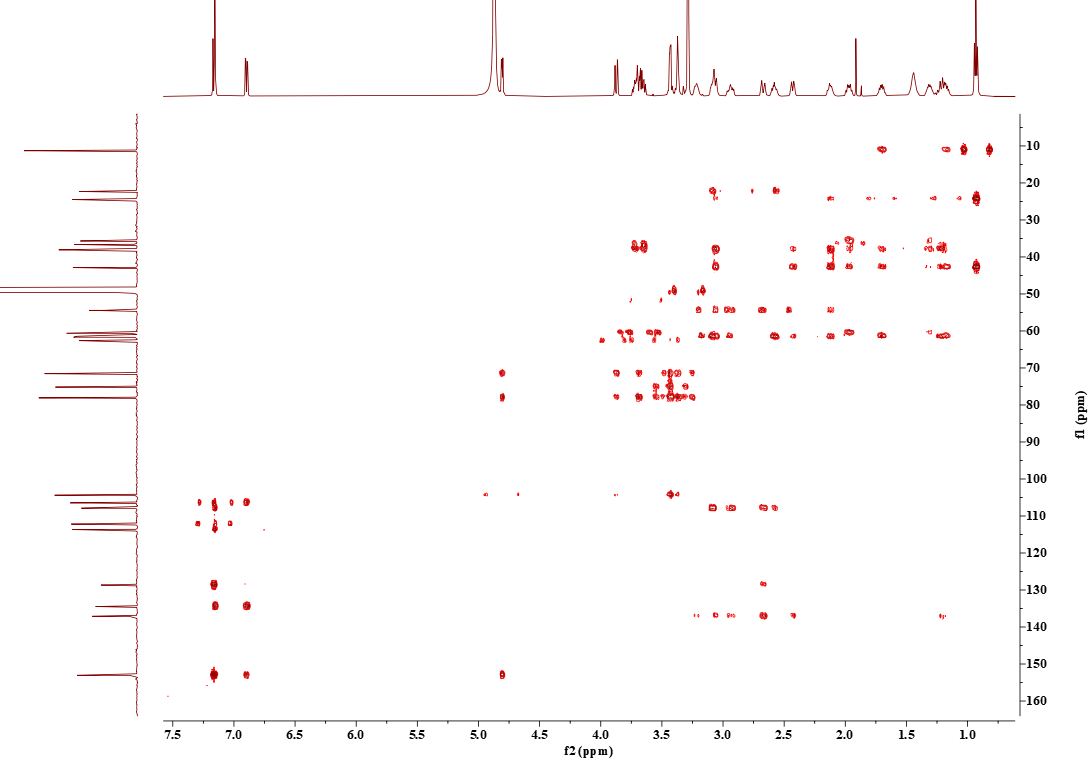


**Figure S8.4 HMBC spectrum of 8**


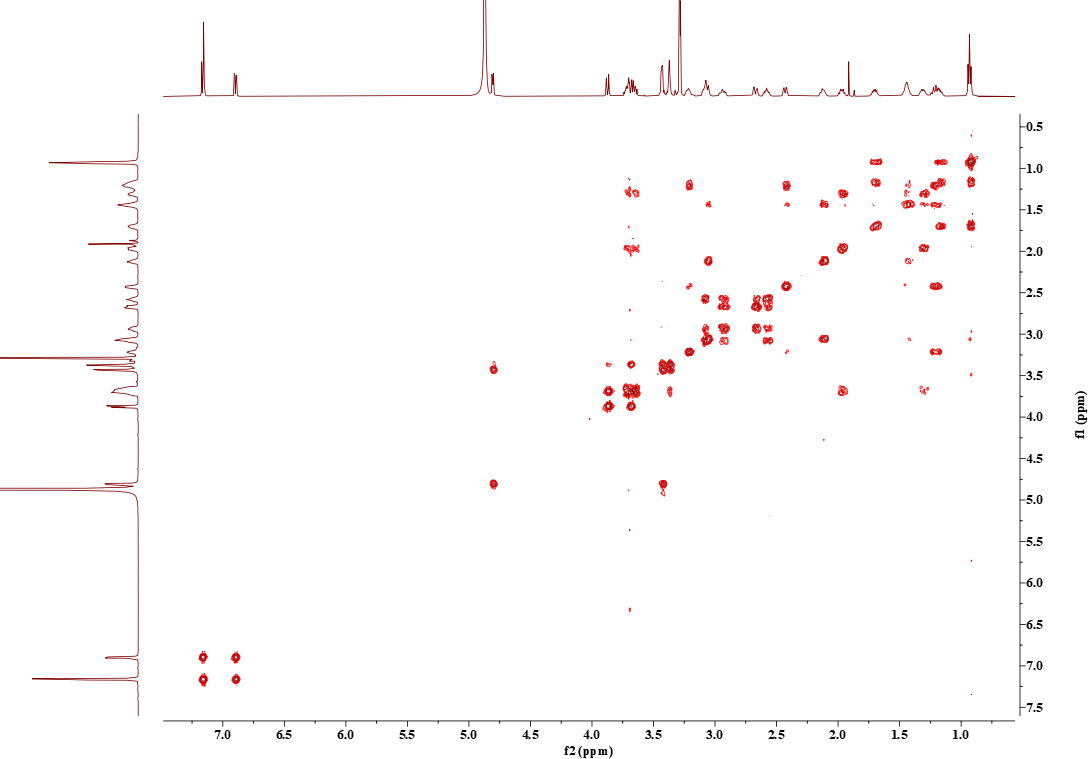


**Figure S8.5 COSY spectrum of 8**


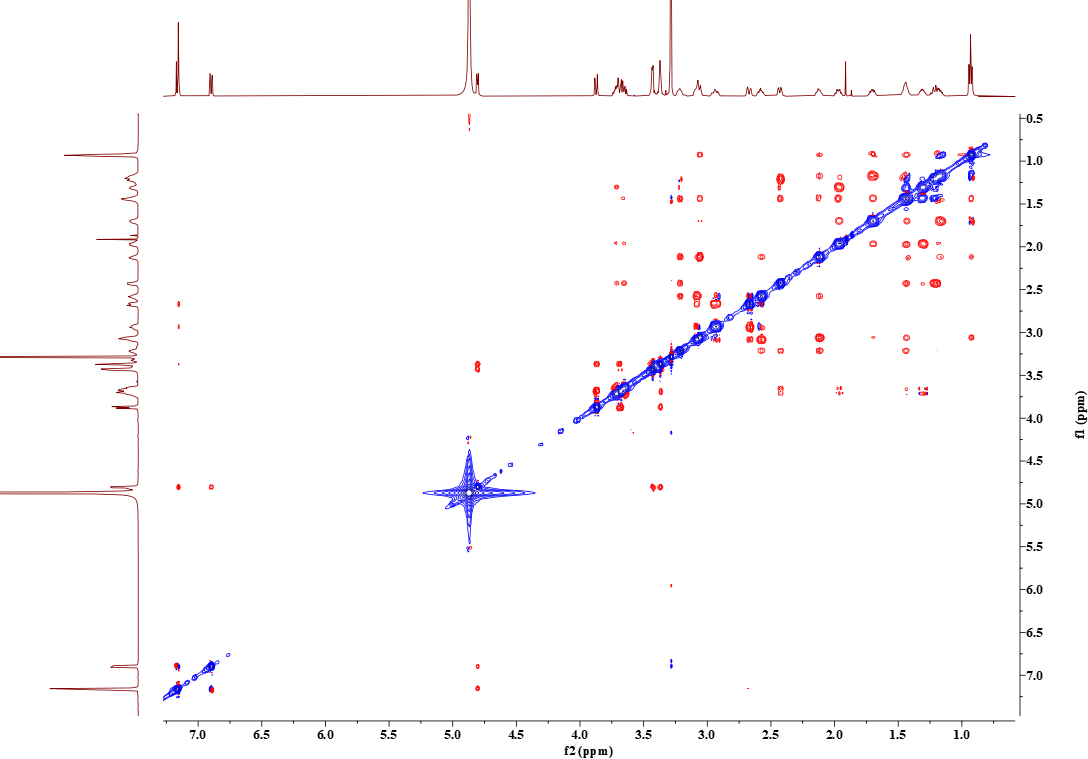


**Figure S8.6 ROESY spectrum of 8**

**Figure S8.7 HRMS spectrum of 8**


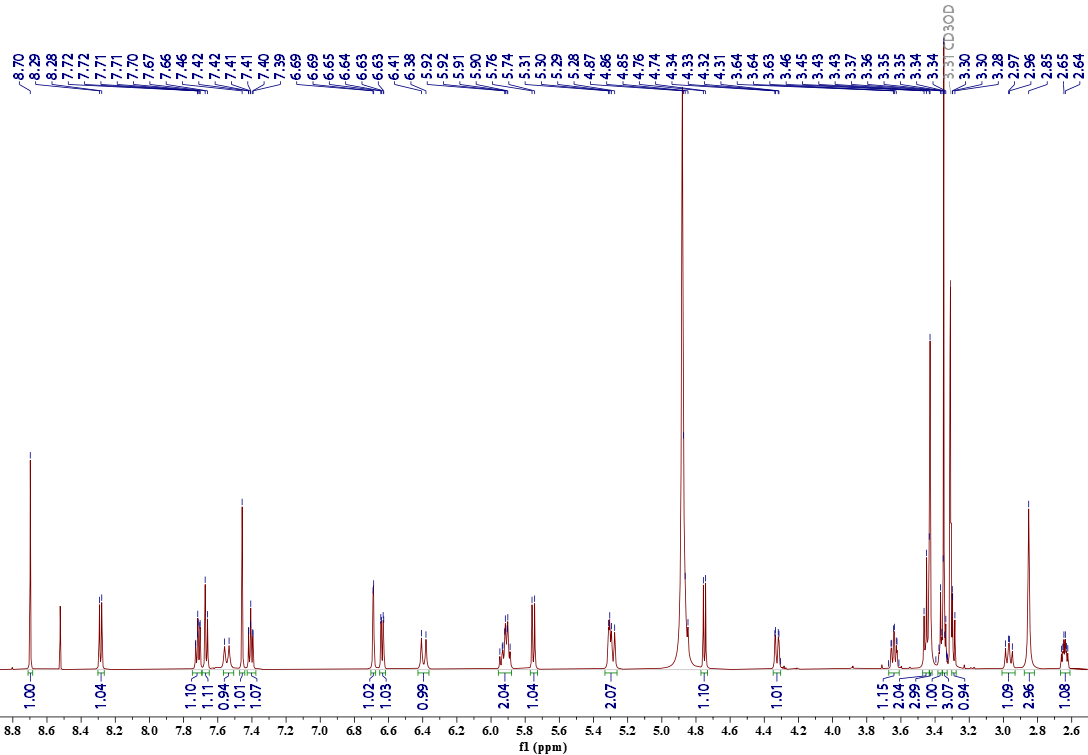


**Figure S9.1 ^1^H NMR spectrum of 9**


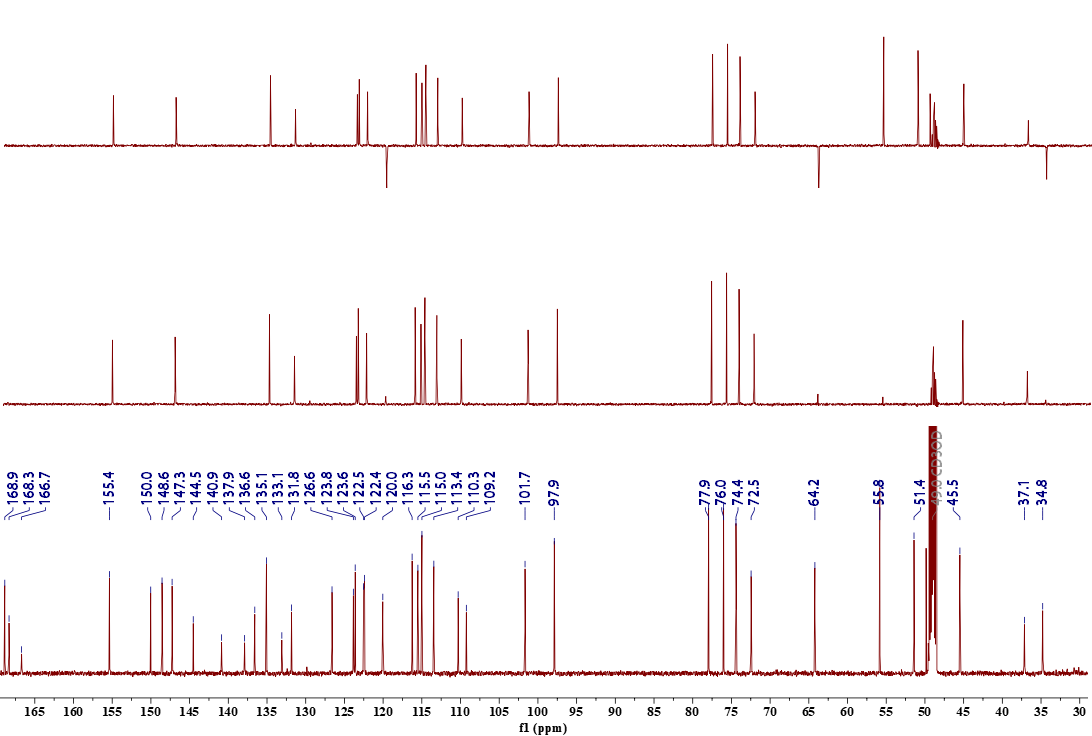


**Figure S9.2 ^13^C NMR spectrum of 9**


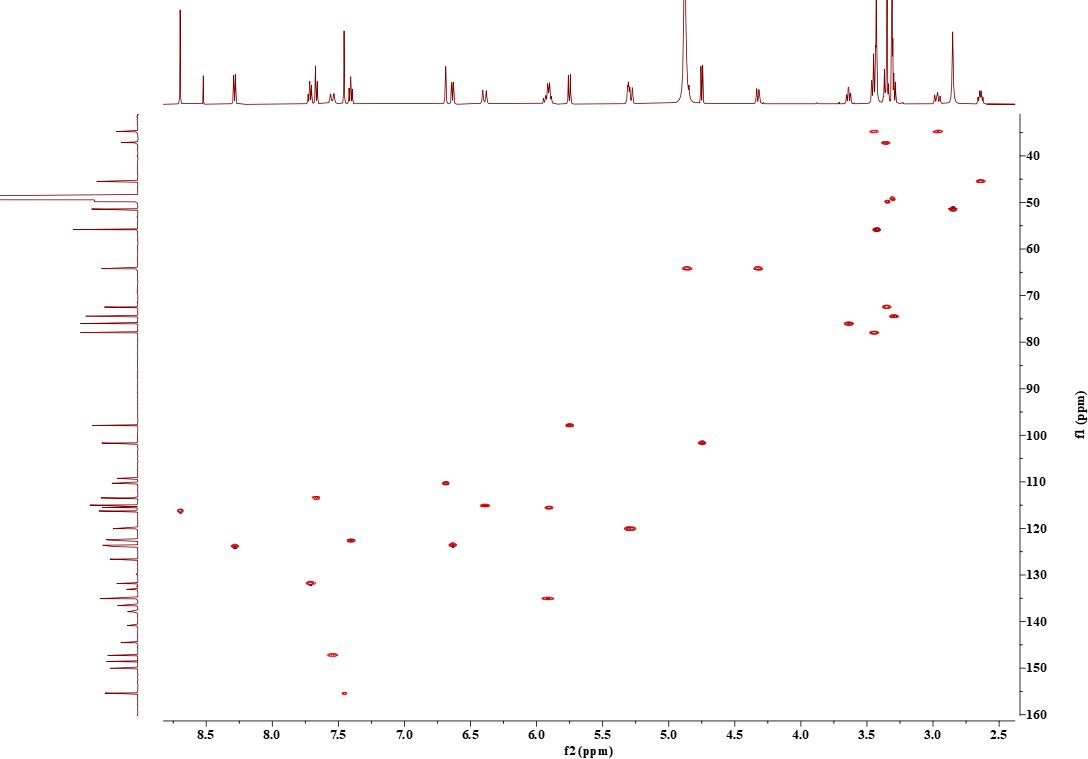


**Figure S9.3 HSQC spectrum of 9**


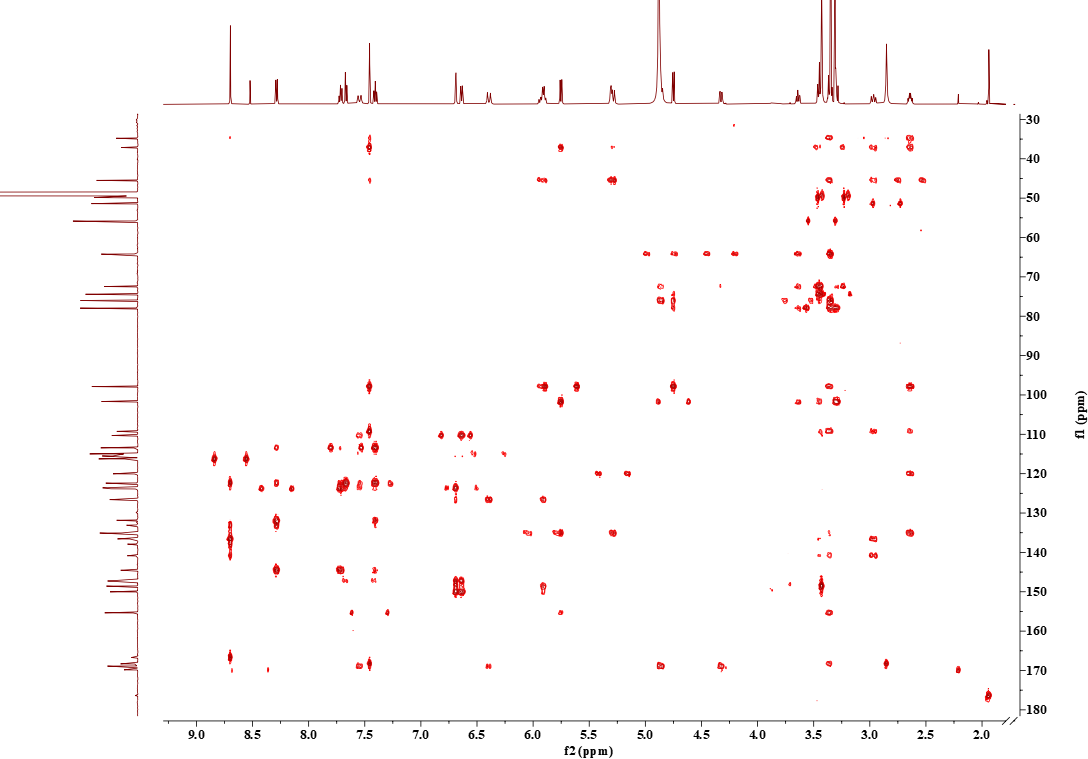


**Figure S9.4 HMBC spectrum of 9**


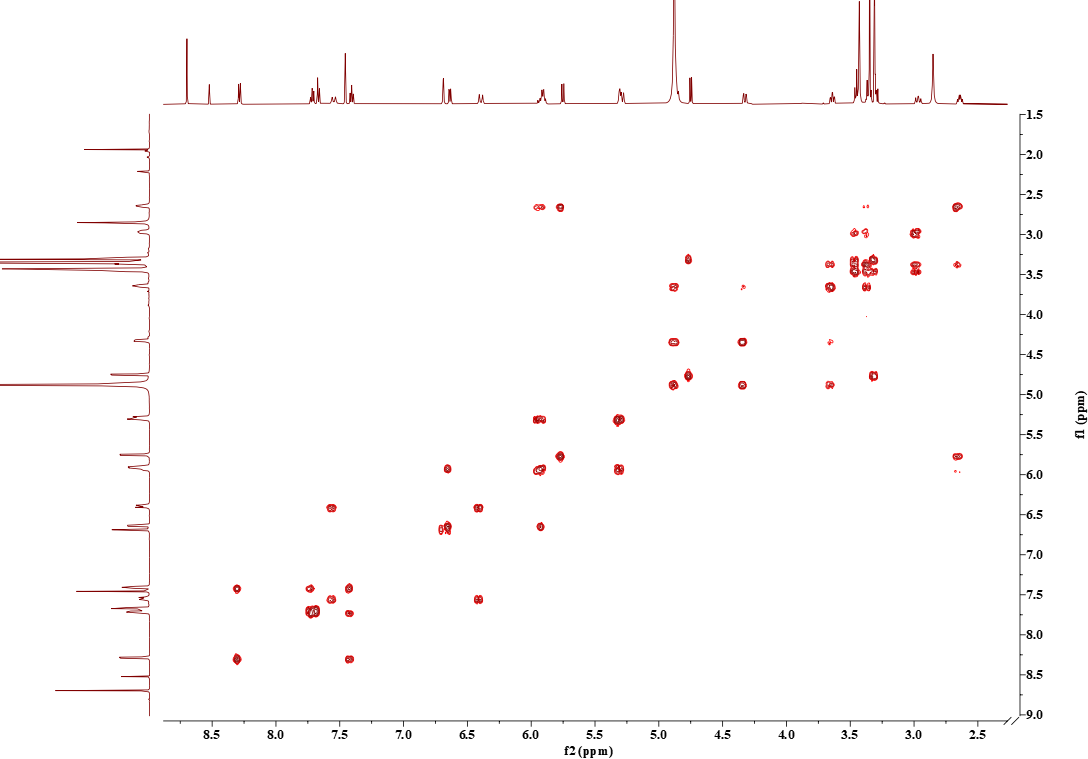


**Figure S9.5 COSY spectrum of 9**


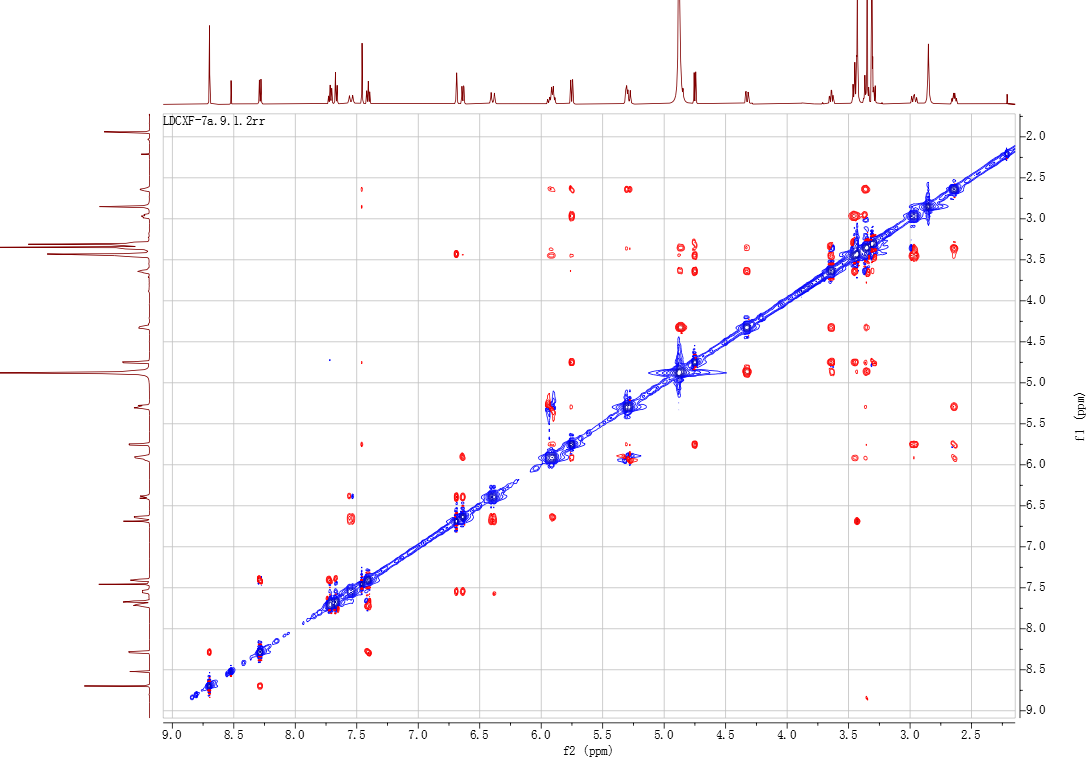


**Figure S9.6 ROESY spectrum of 9**

**Figure S9.7 HRMS spectrum of 9**


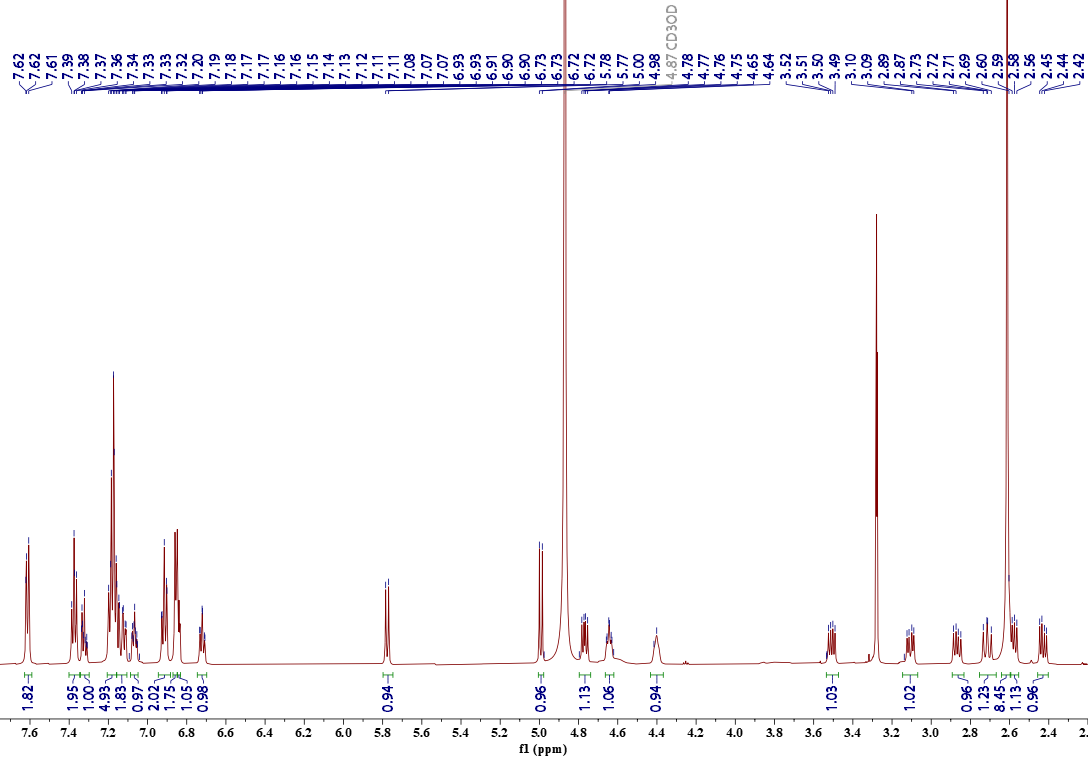


**Figure S10.1 ^1^H NMR spectrum of 10**


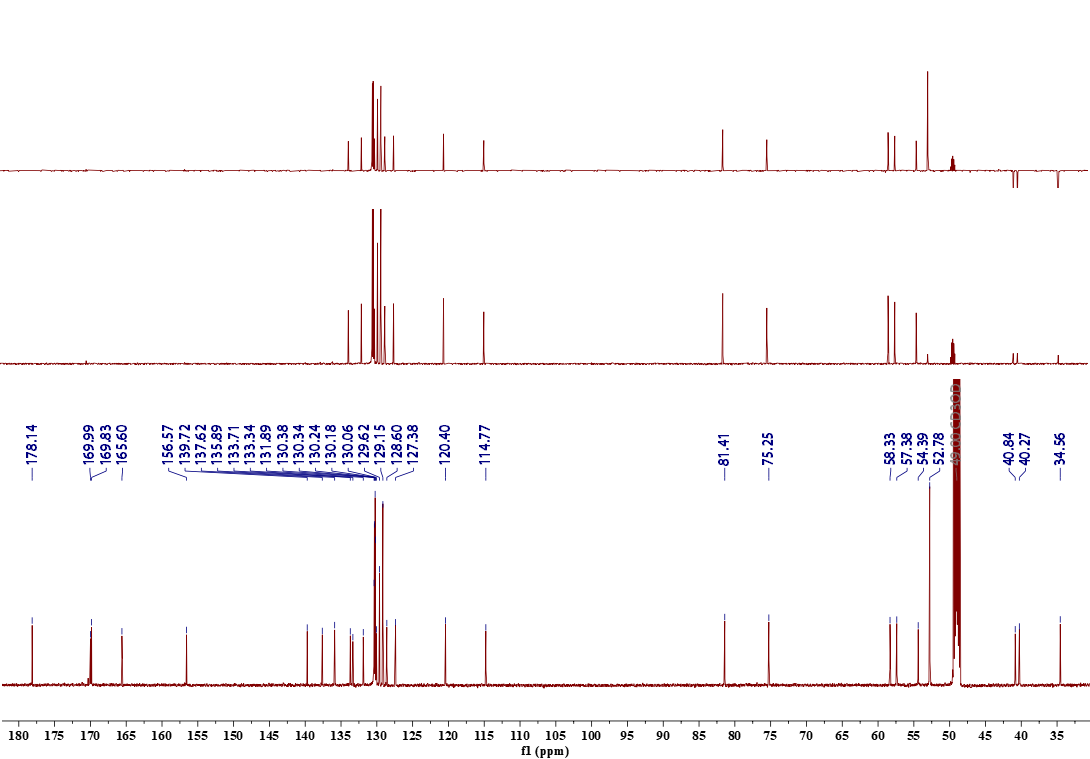


**Figure S10.2 ^13^C NMR spectrum of 10**


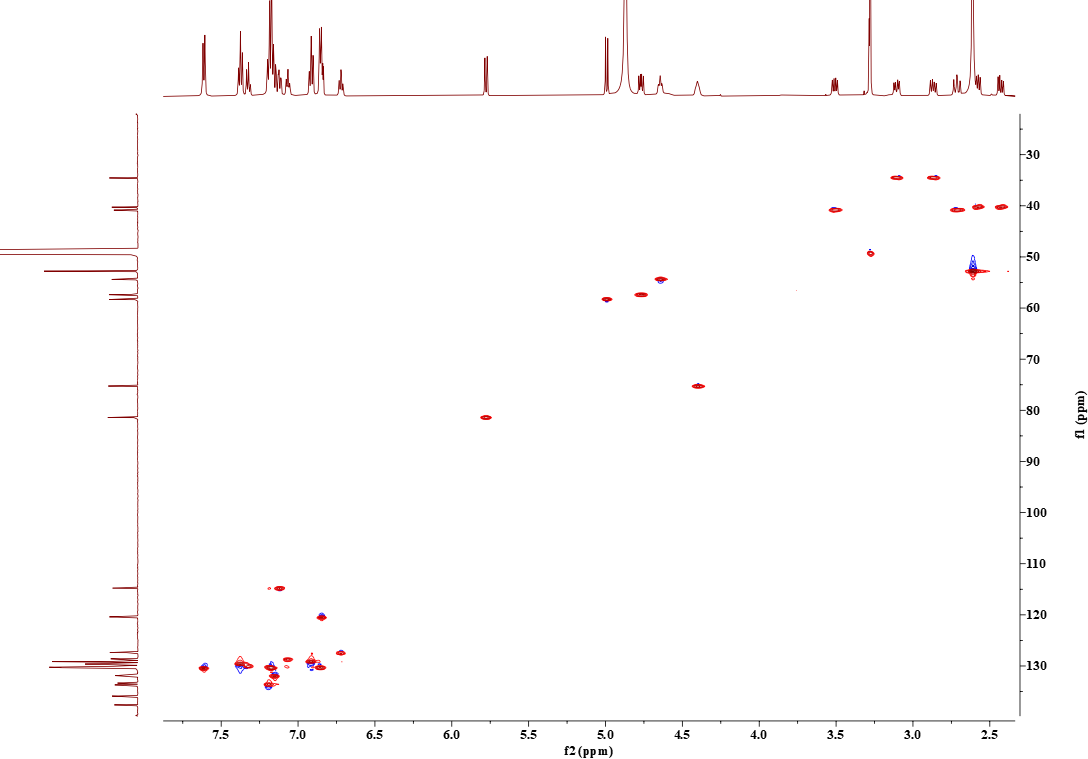


**Figure S10.3 HSQC spectrum of 10**


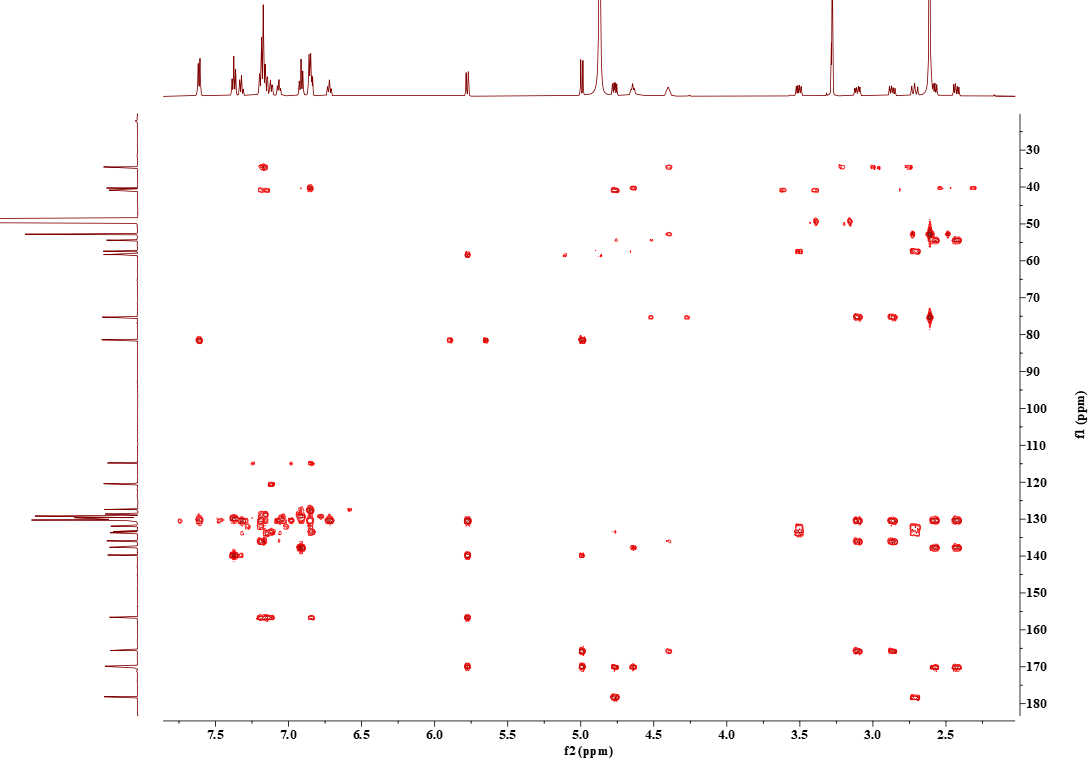


**Figure S10.4 HMBC spectrum of 10**


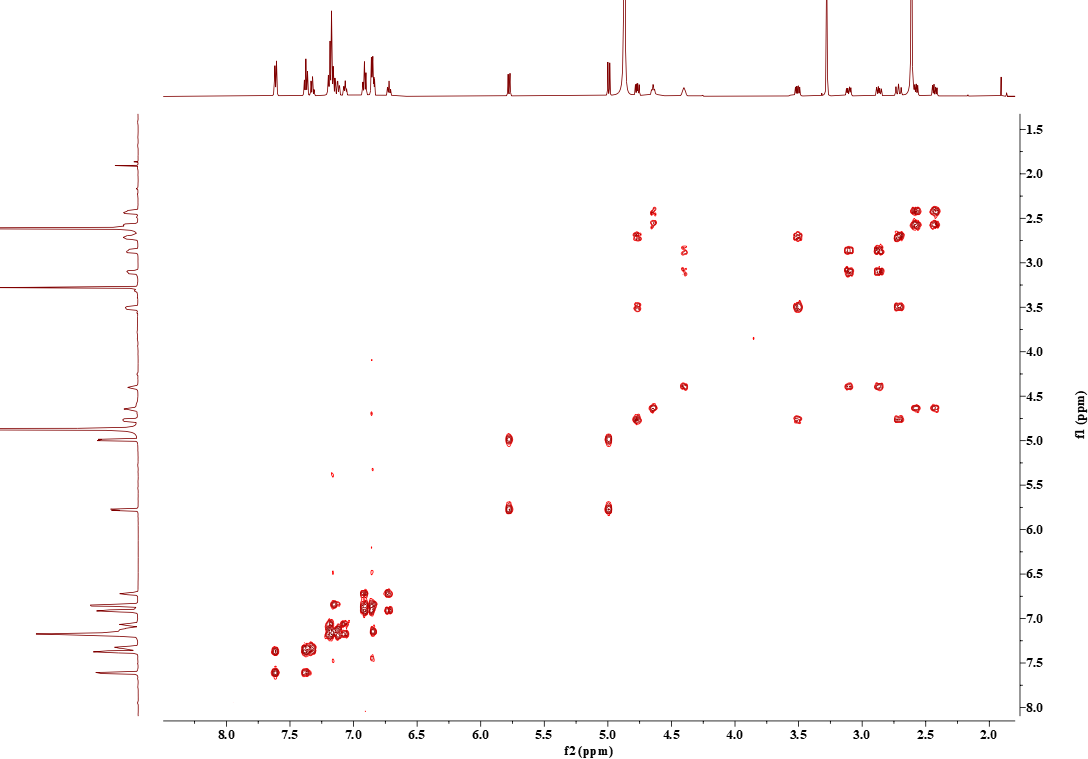


**Figure S10.5 COSY spectrum of 10**


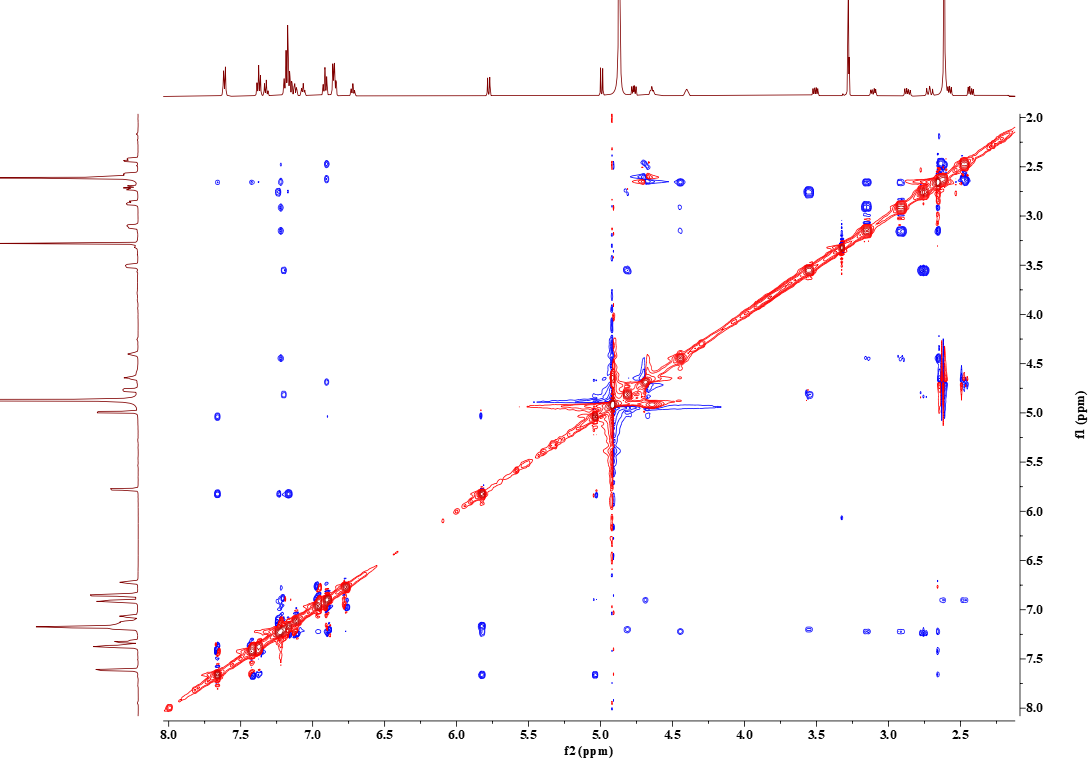


**Figure S10.6 ROESY spectrum of 10**

**Figure S10.7 HRMS spectrum of 10**


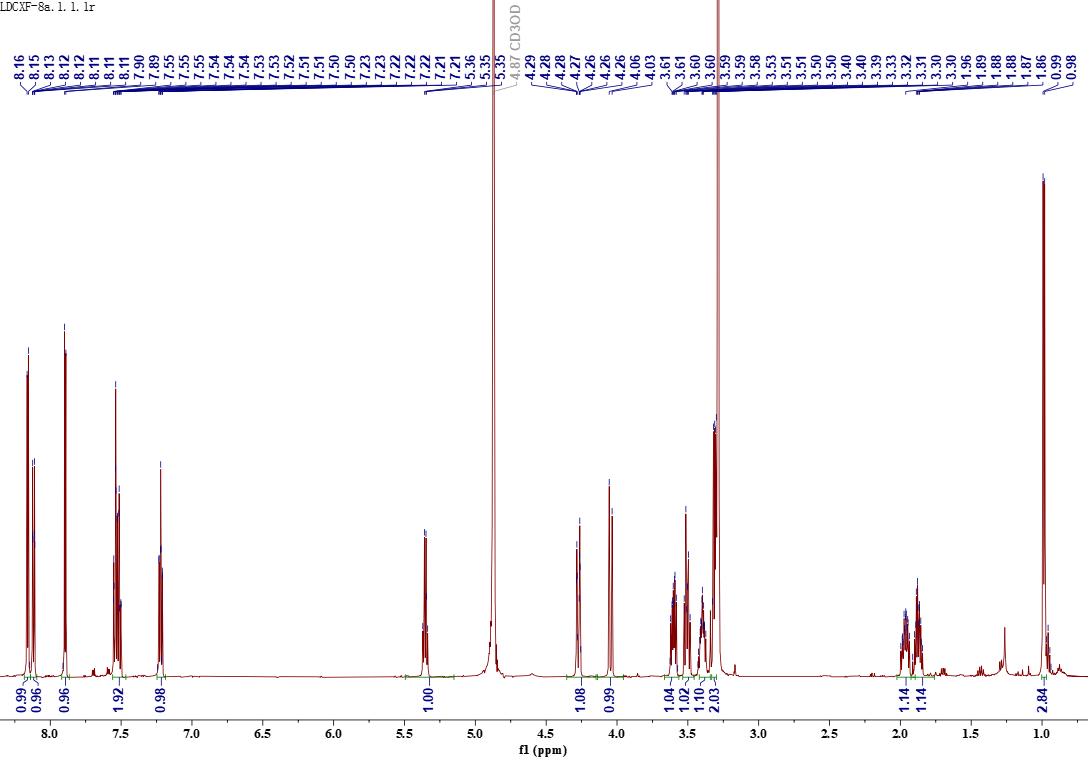


**Figure S11.1 ^1^H NMR spectrum of 11**


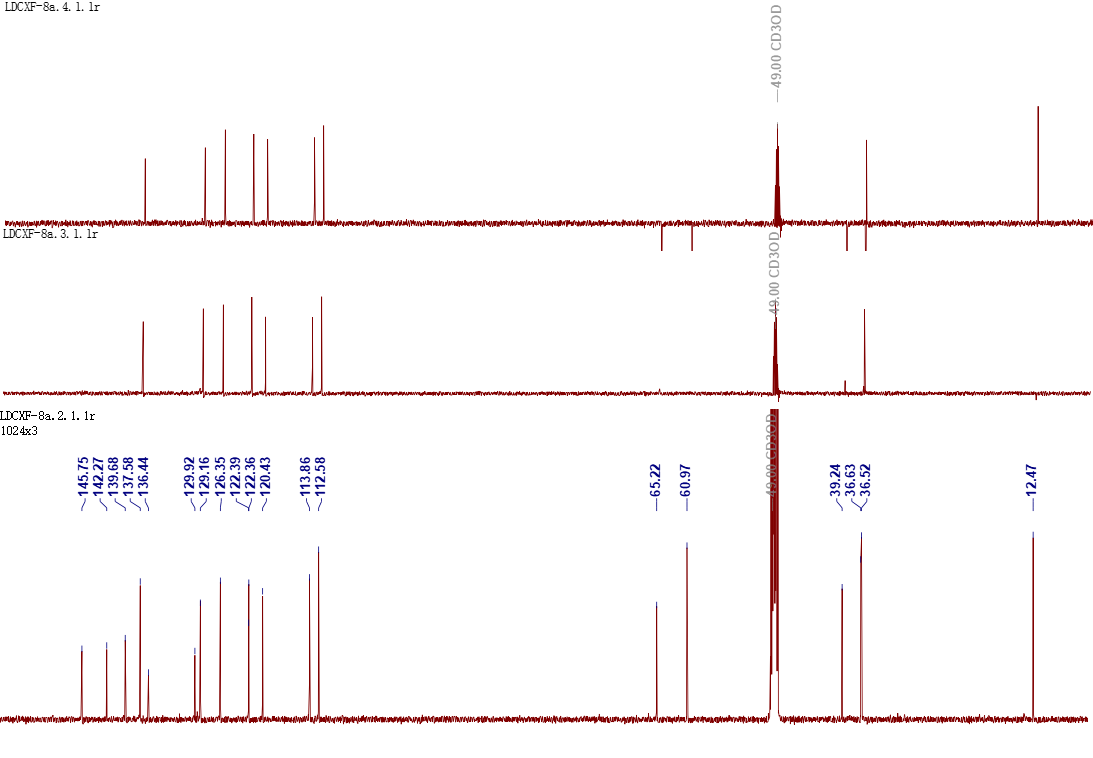


**Figure S11.2 ^13^C NMR spectrum of 11**


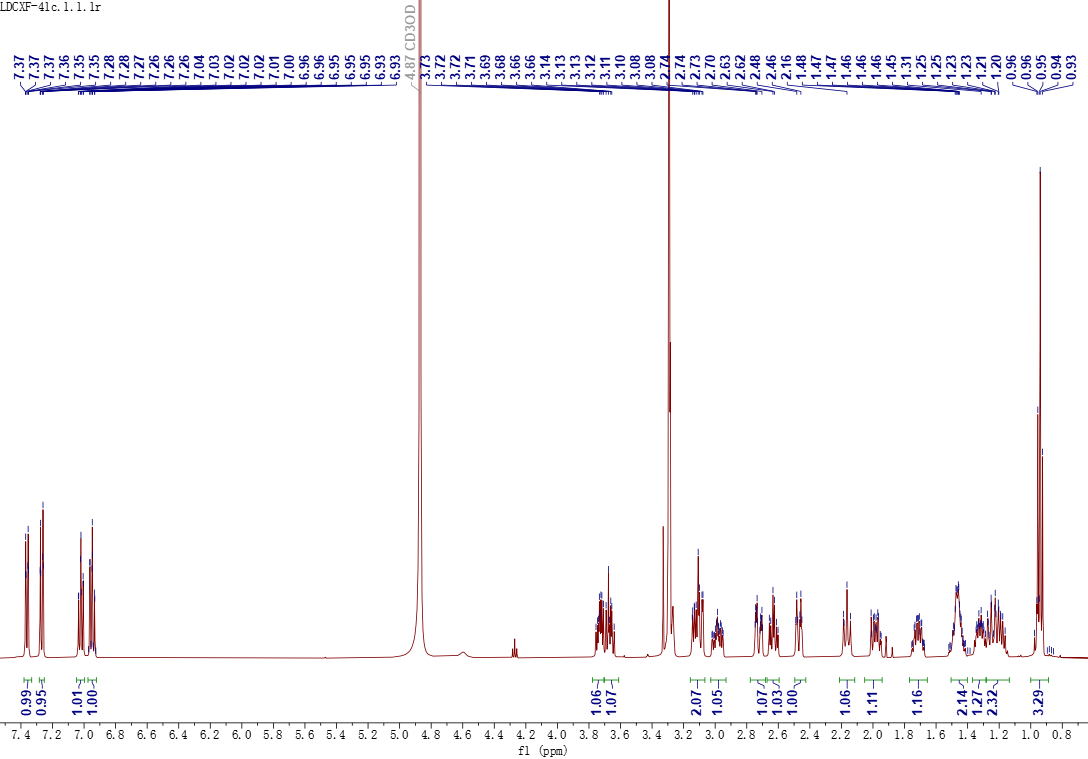


**Figure S12.1 ^1^H NMR spectrum of 12**


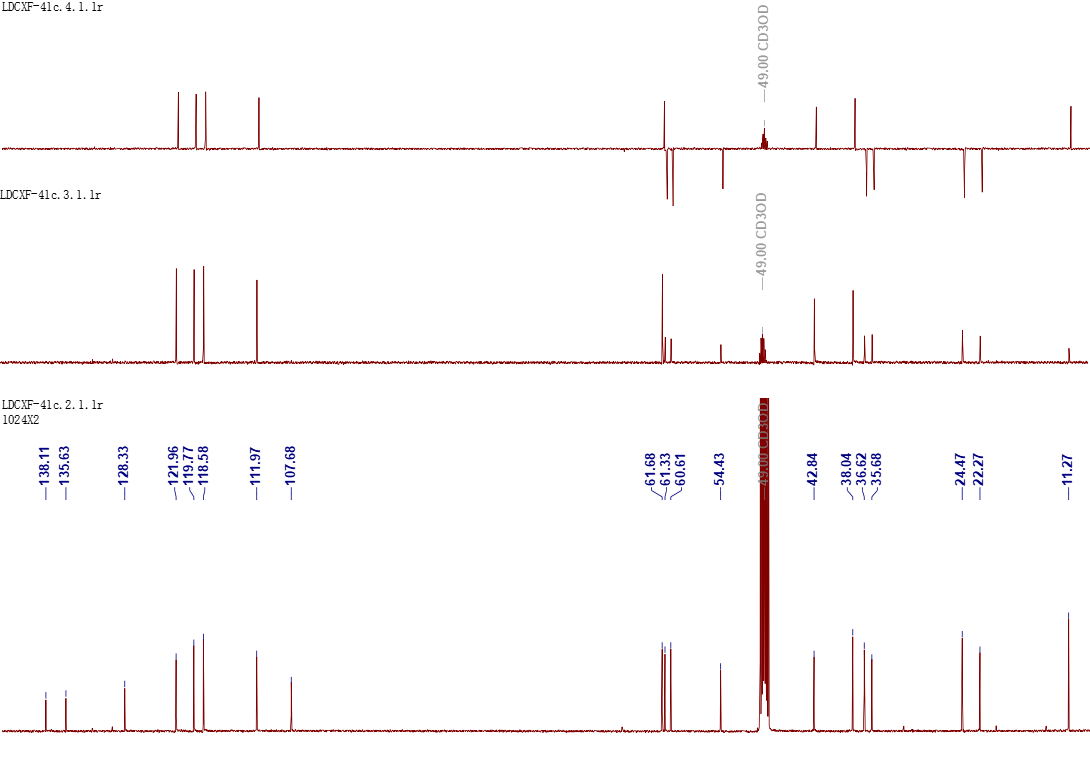


**Figure S12.2 ^13^C NMR spectrum of 12**


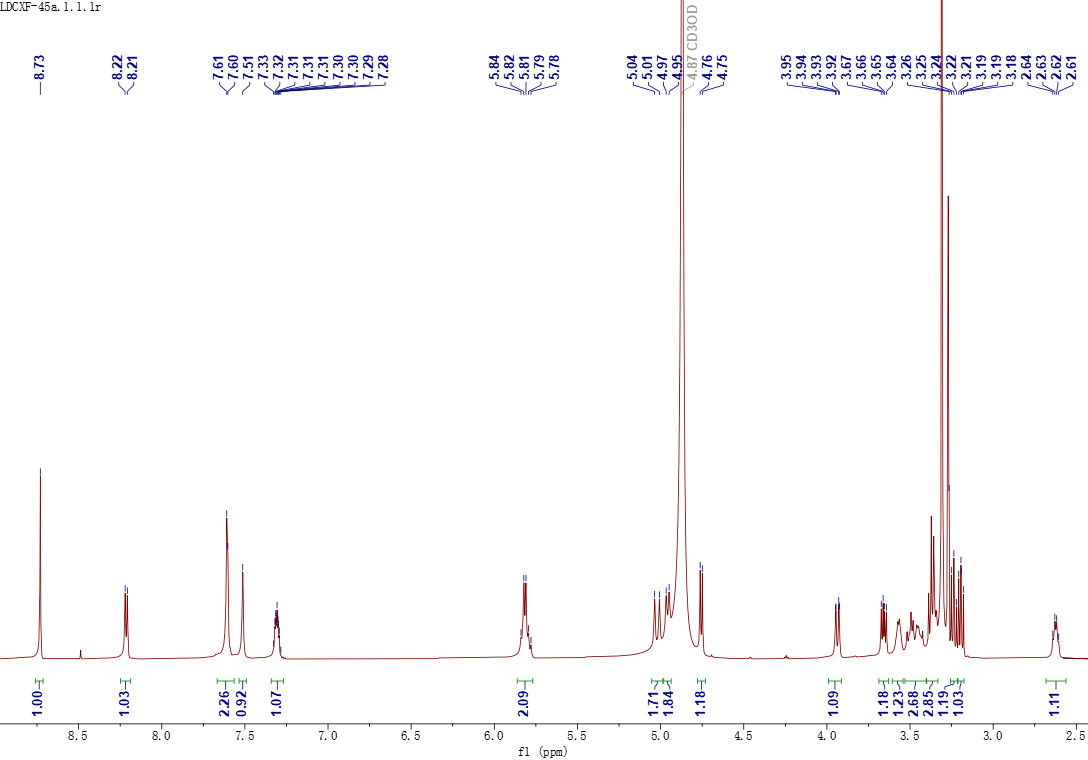


**Figure S13.1 ^1^H NMR spectrum of 13**


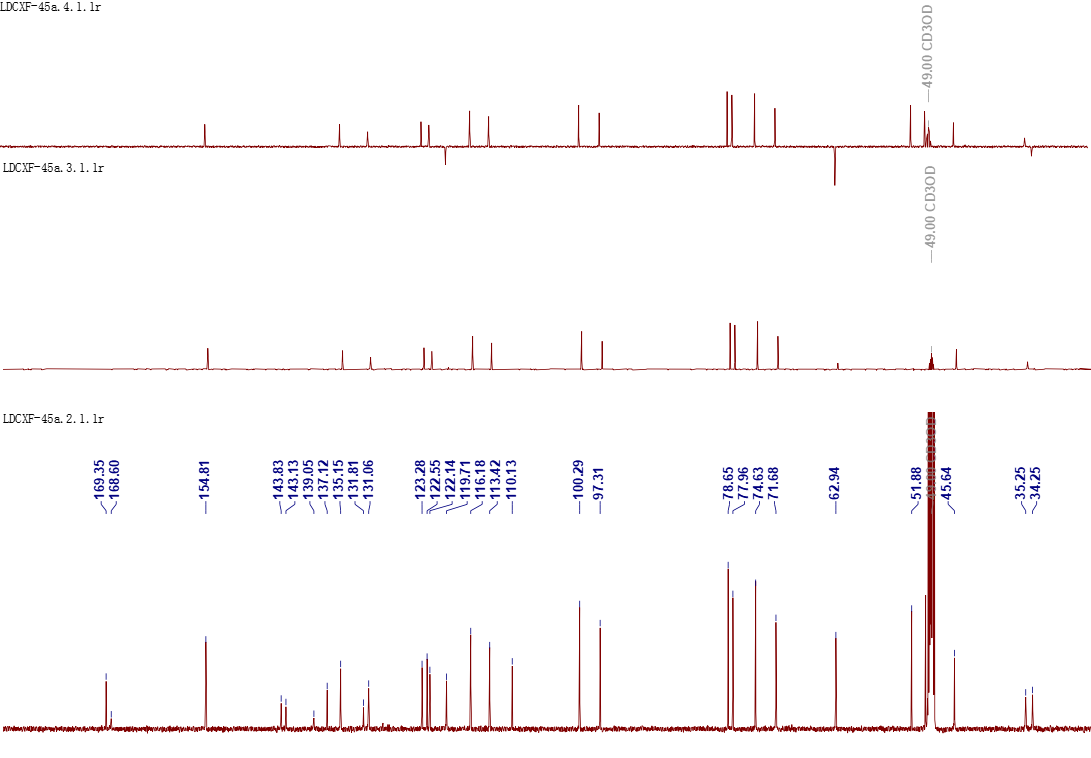


**Figure S13.2 ^13^C NMR spectrum of 13**


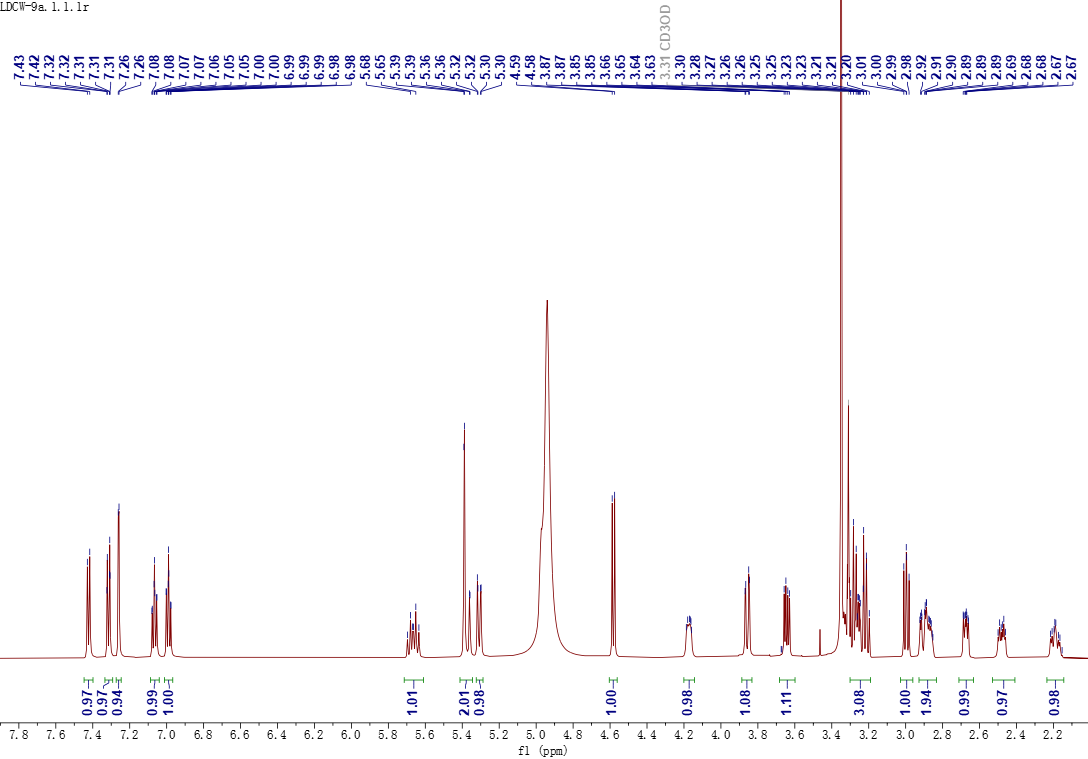


**Figure S14.1 ^1^H NMR spectrum of 14**


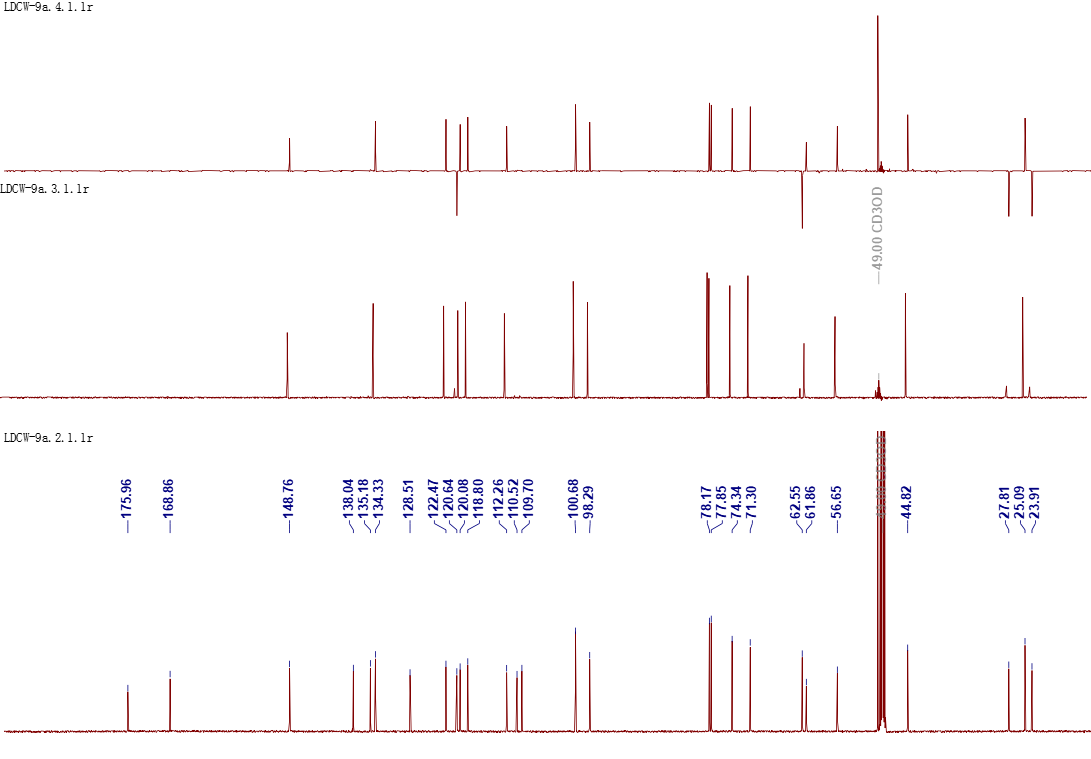


**Figure S14.2 ^13^C NMR spectrum of 14**


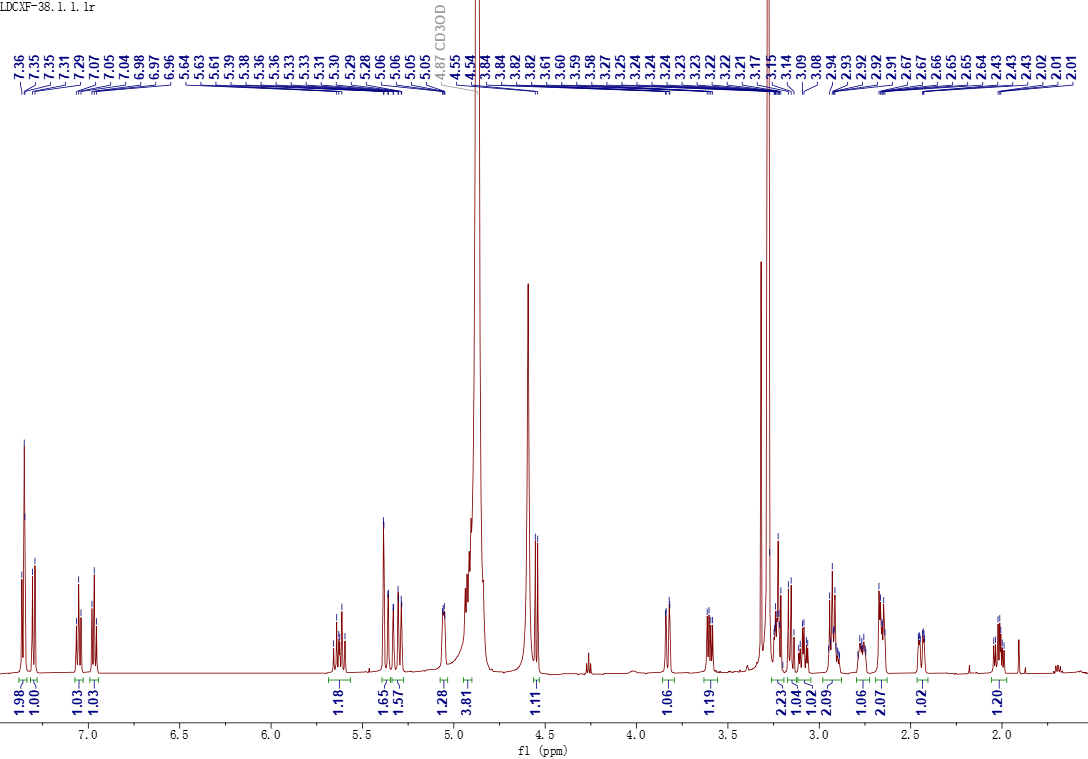


**Figure S15.1 ^1^H NMR spectrum of 15**


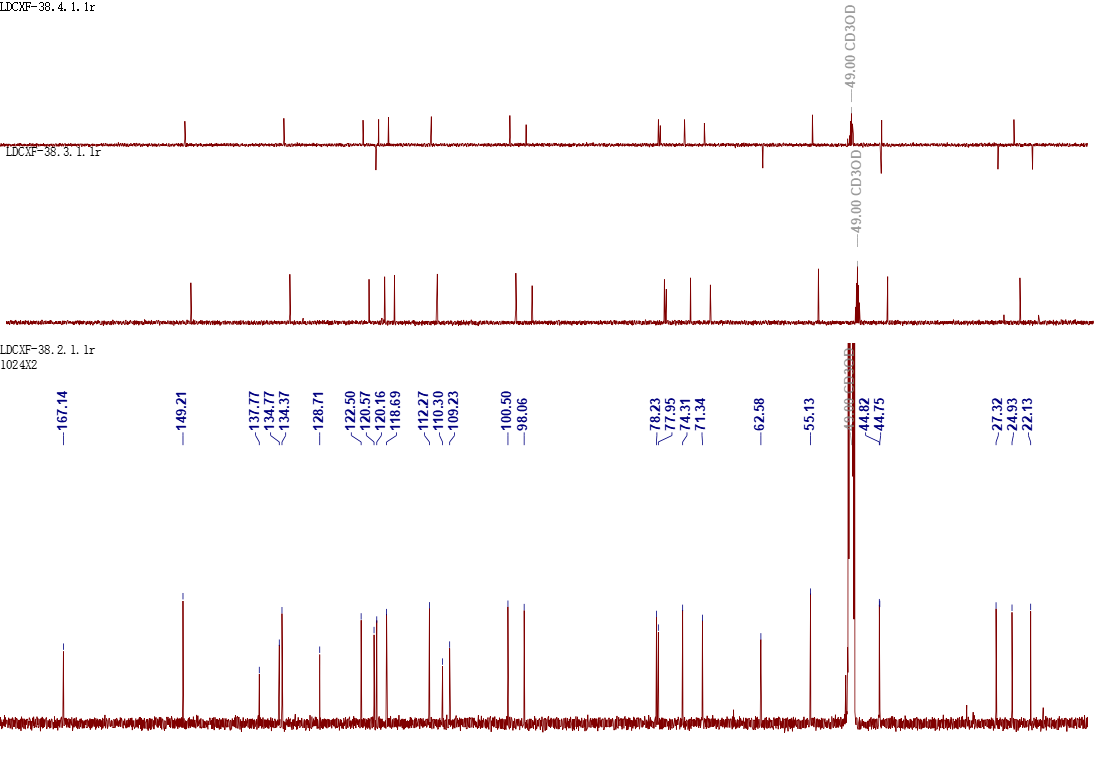


**Figure S15.2 ^13^C NMR spectrum of 15**


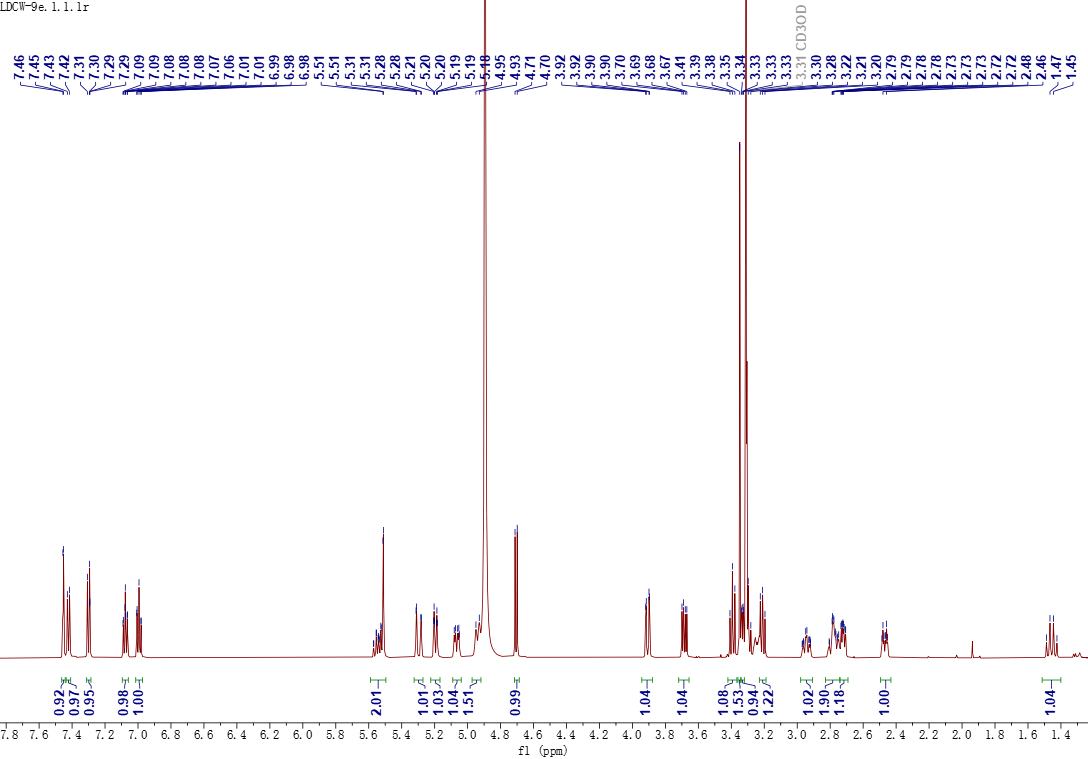


**Figure S16.1 ^1^H NMR spectrum of 16**


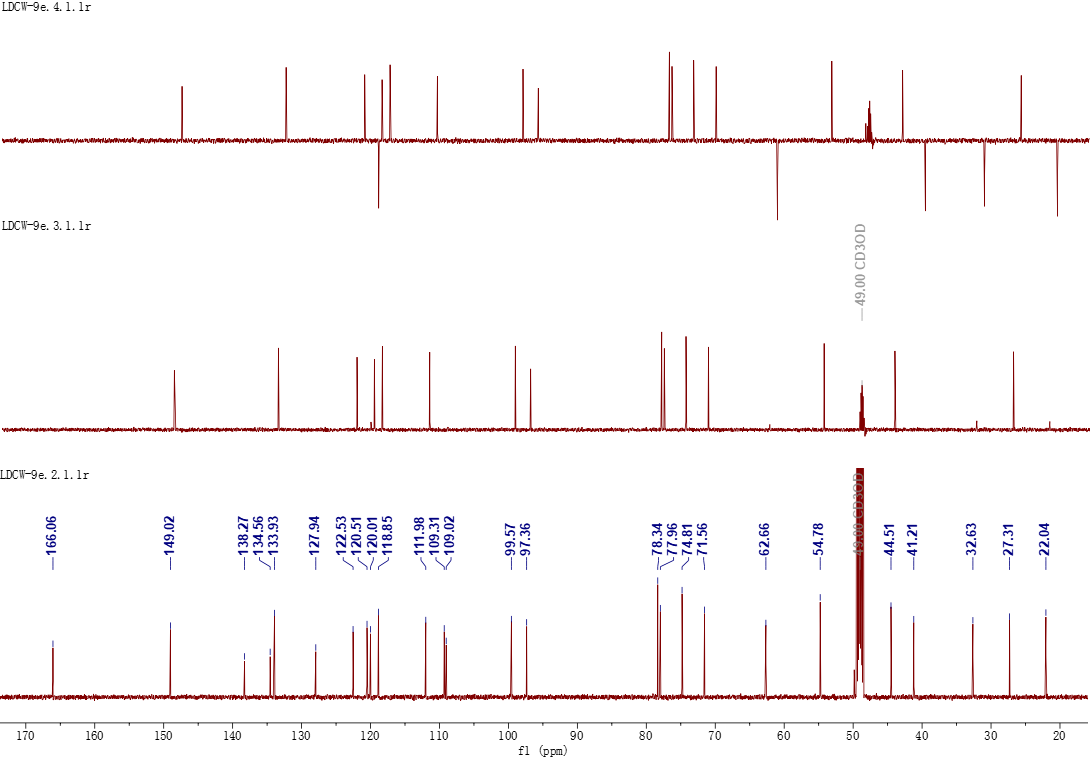


**Figure S16.2 ^13^C NMR spectrum of 16**


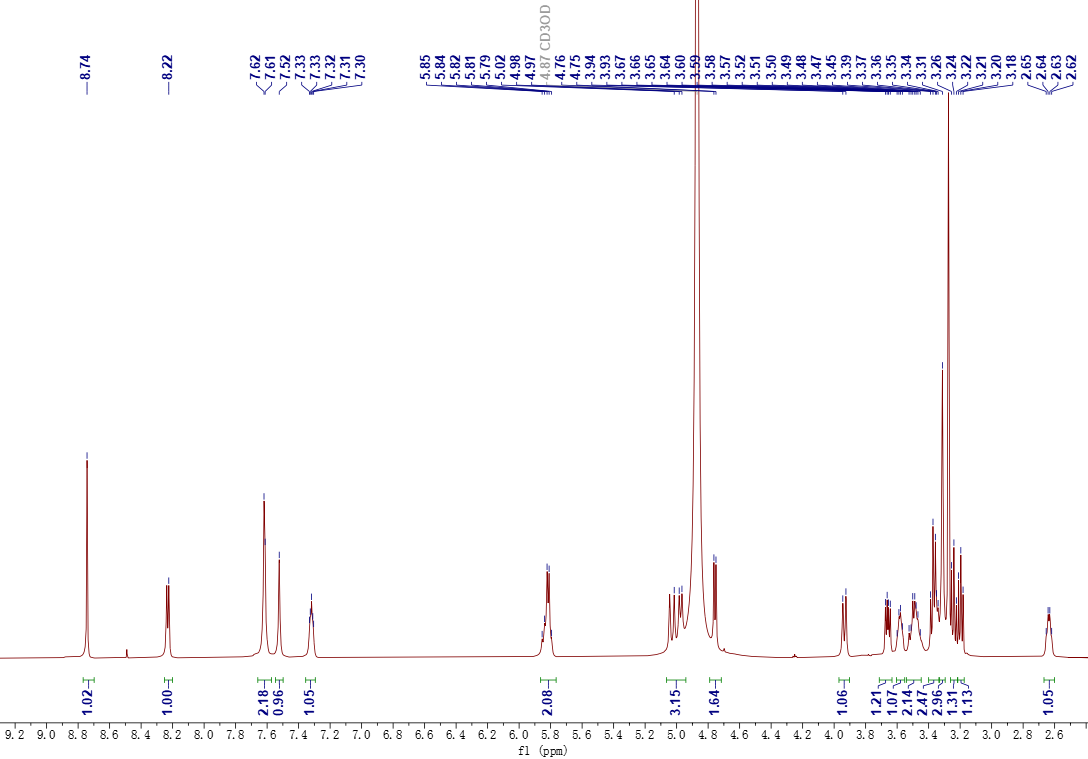


**Figure S17.1 ^1^H NMR spectrum of 17**


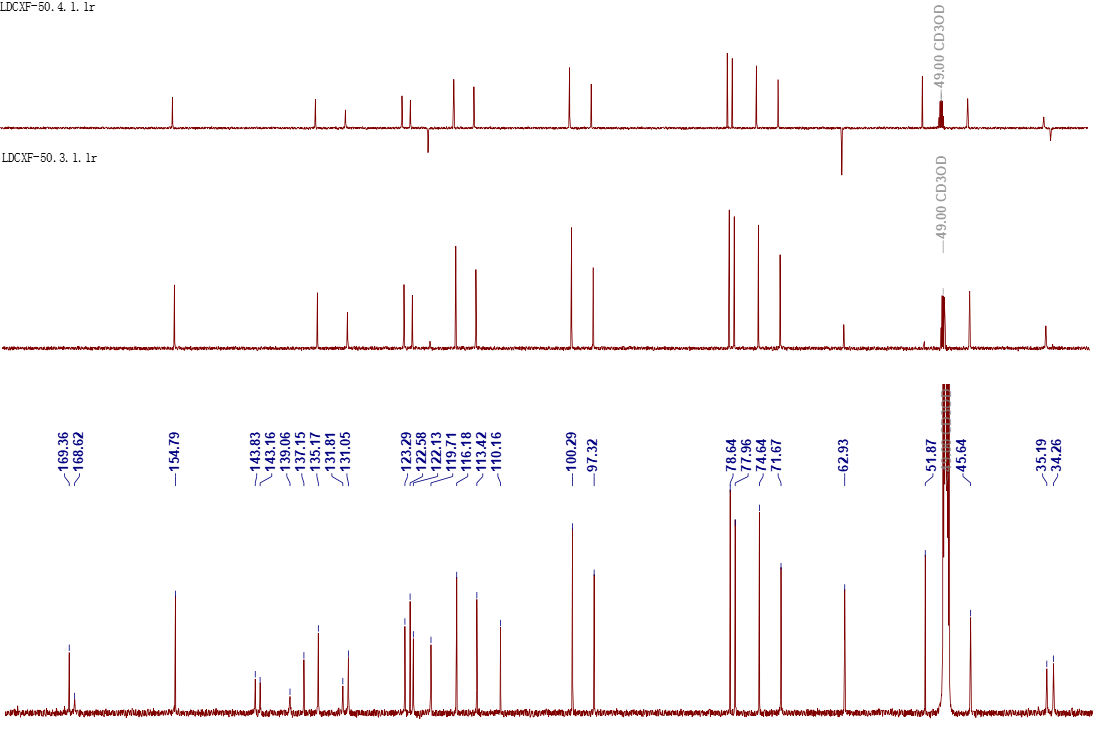


**Figure S17.2 ^13^C NMR spectrum of 17**


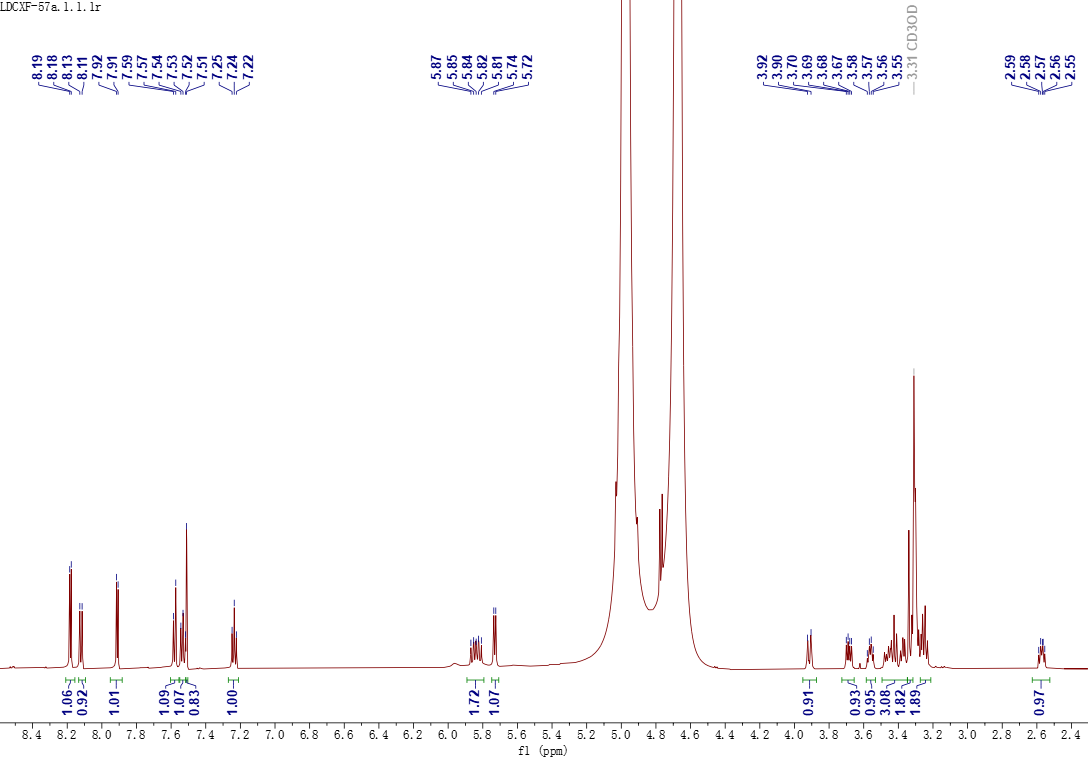


**Figure S18.1 ^1^H NMR spectrum of 18**


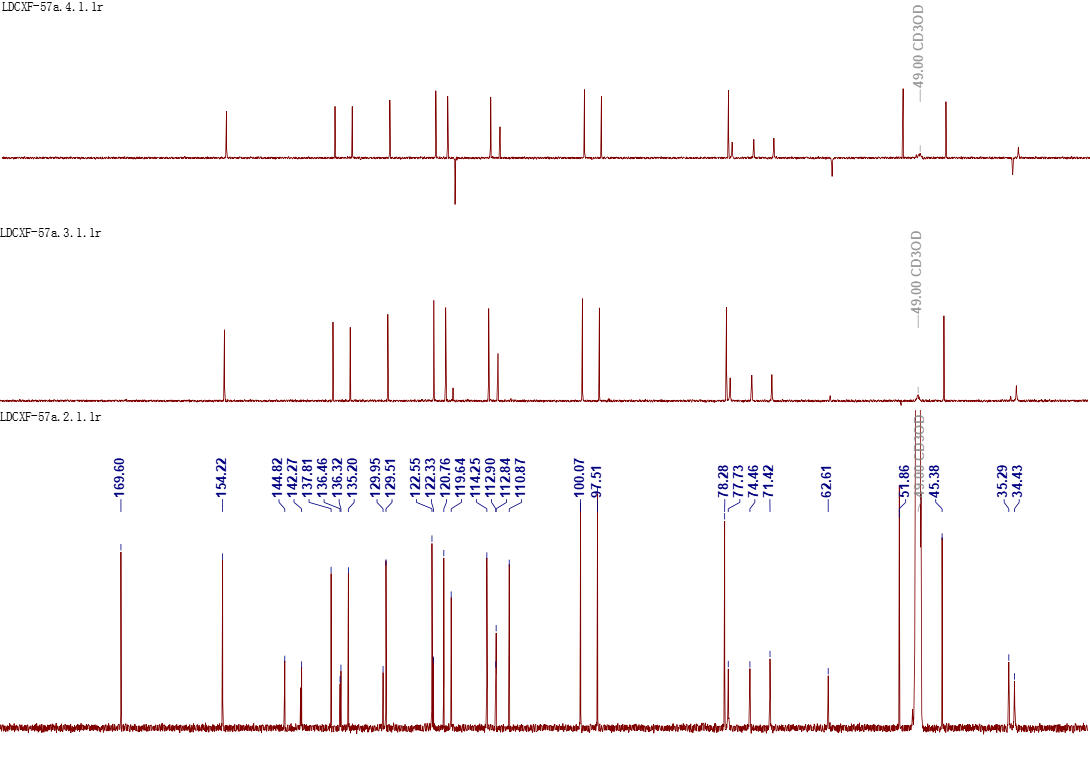


**Figure S18.2 ^13^C NMR spectrum of 18**


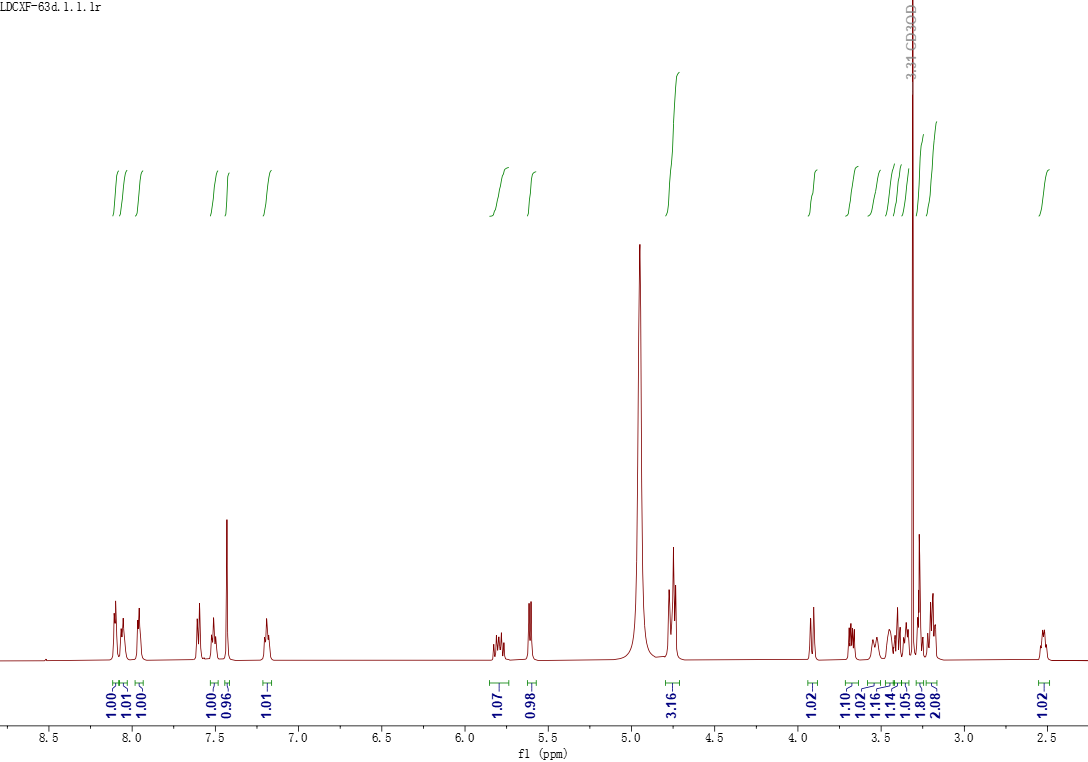


**Figure S19.1 ^1^H NMR spectrum of 19**

**
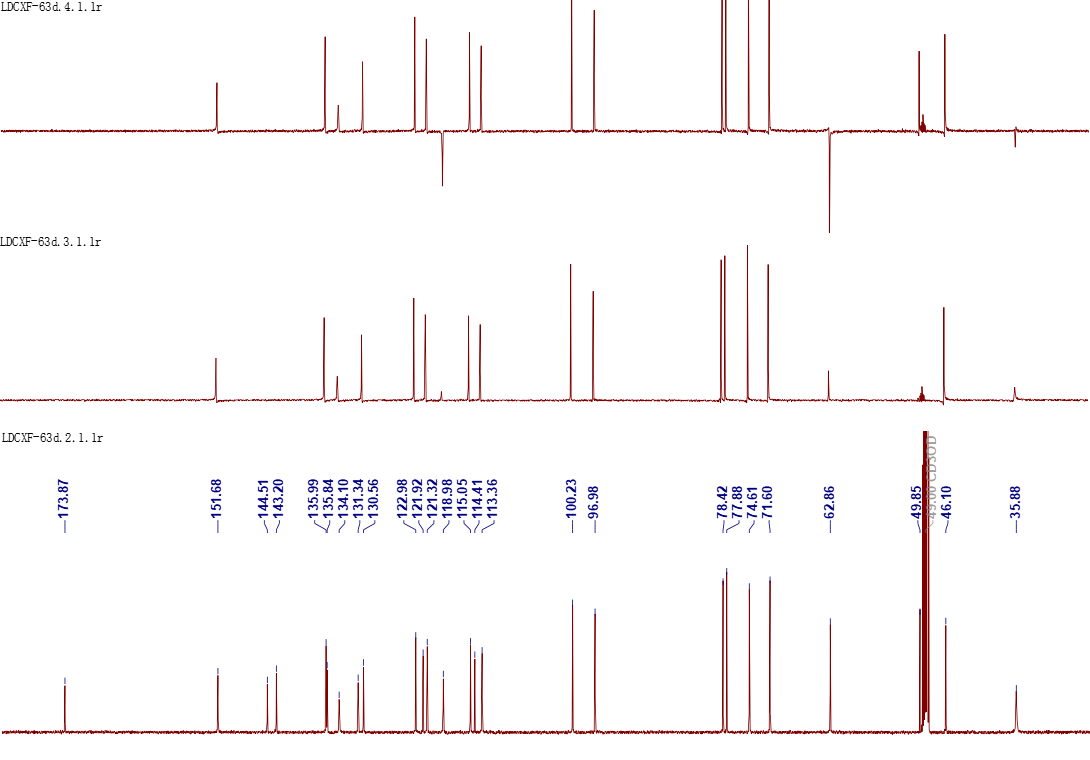
**

**Figure S19.2 ^13^C NMR spectrum of 19**


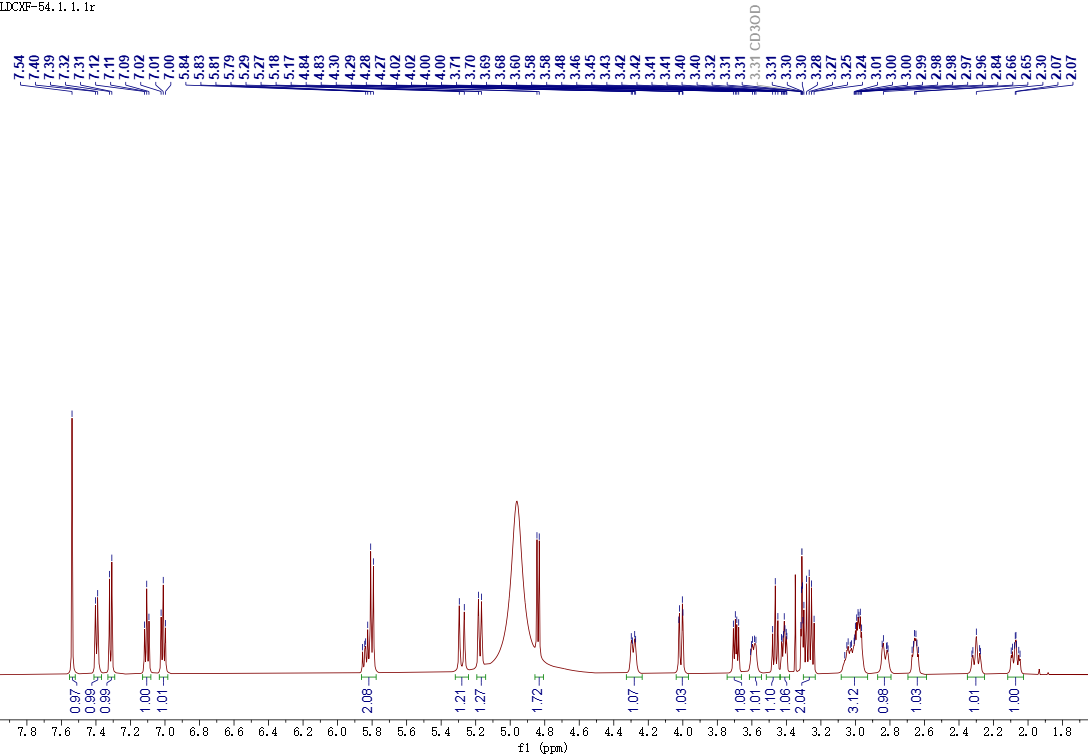


**Figure S20.1 ^1^H NMR spectrum of 20**


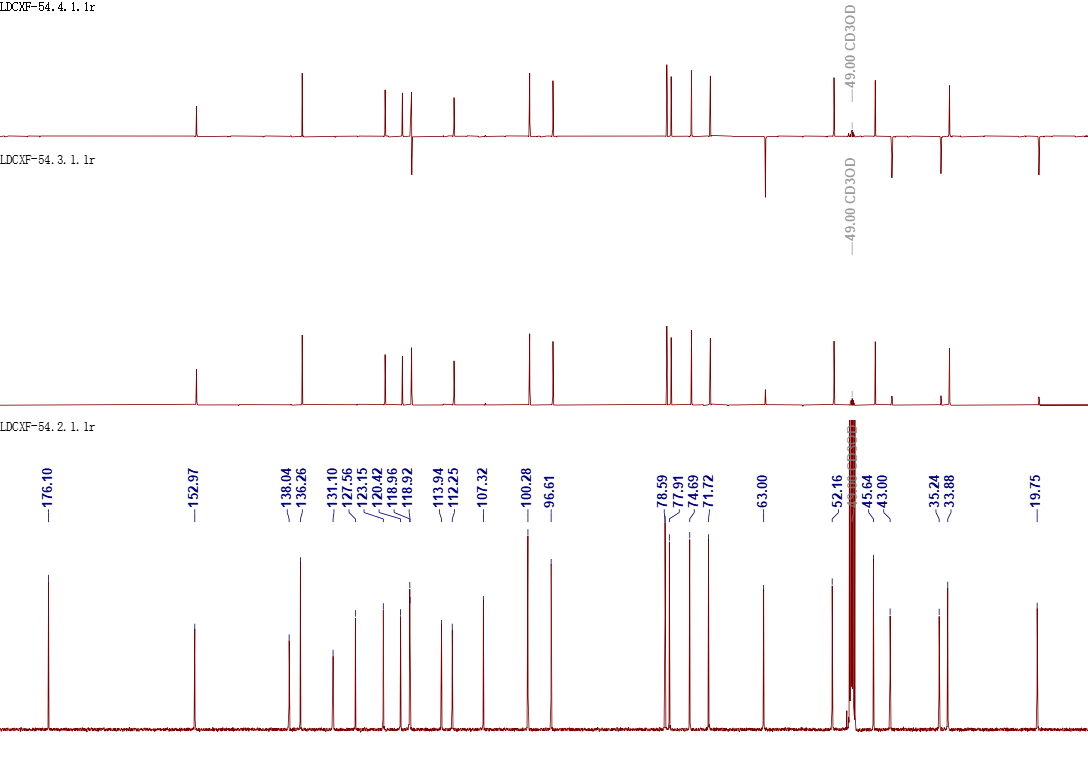


**Figure S20.2 ^13^C NMR spectrum of 20**

**
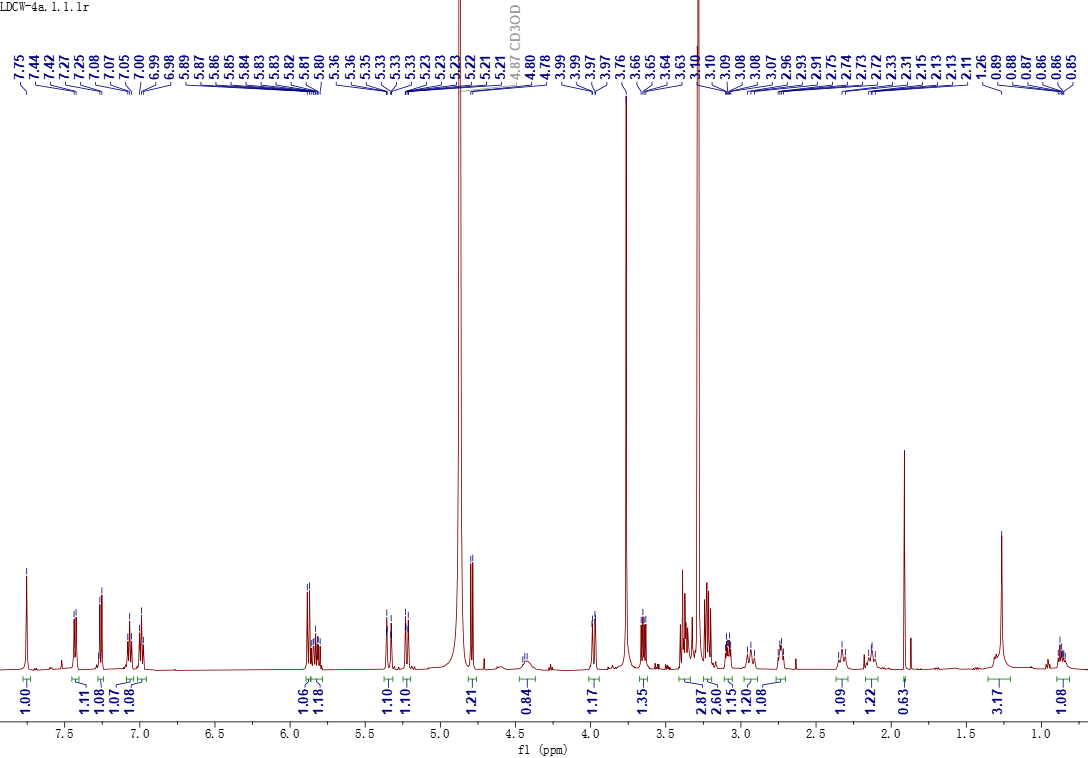
**

**Figure S21.1 ^1^H NMR spectrum of 21**


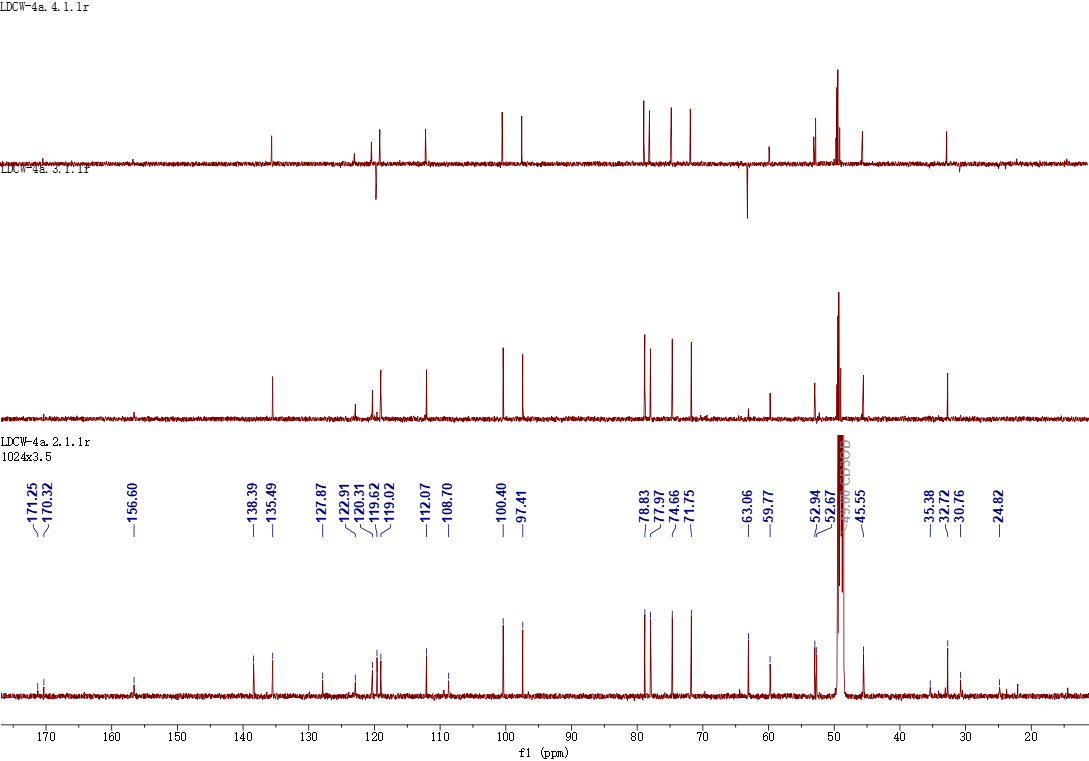


**Figure S21.2 ^13^C NMR spectrum of 21**


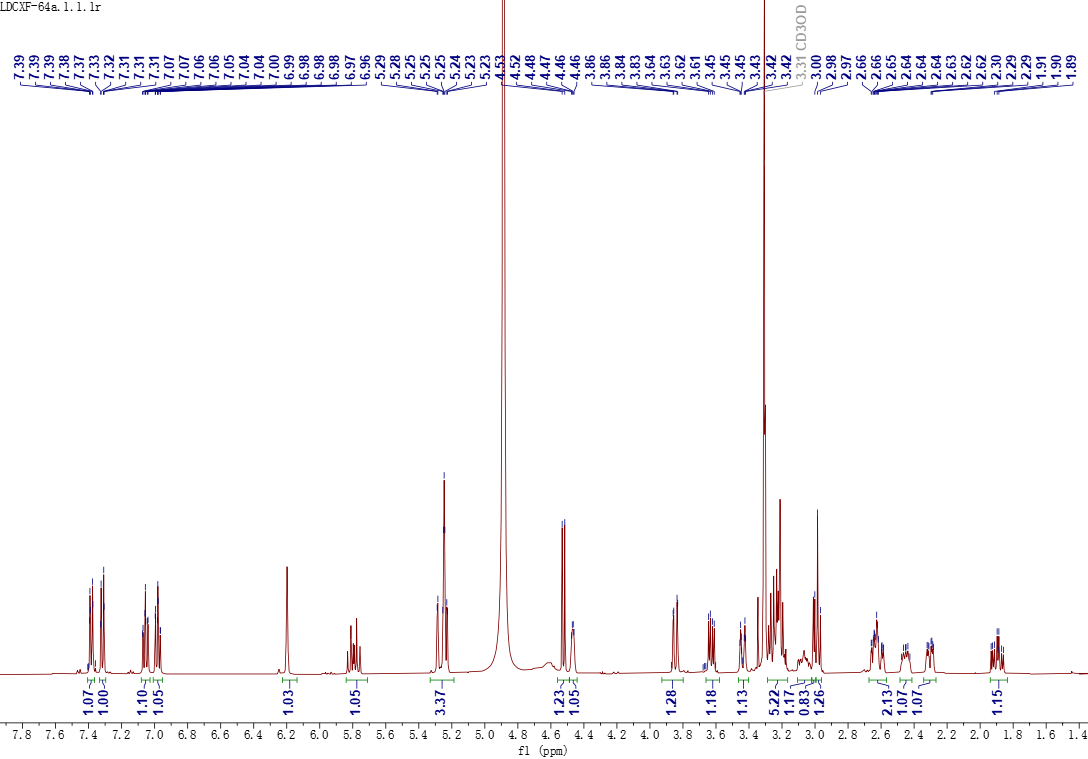


**Figure S21.1 ^1^H NMR spectrum of 21**


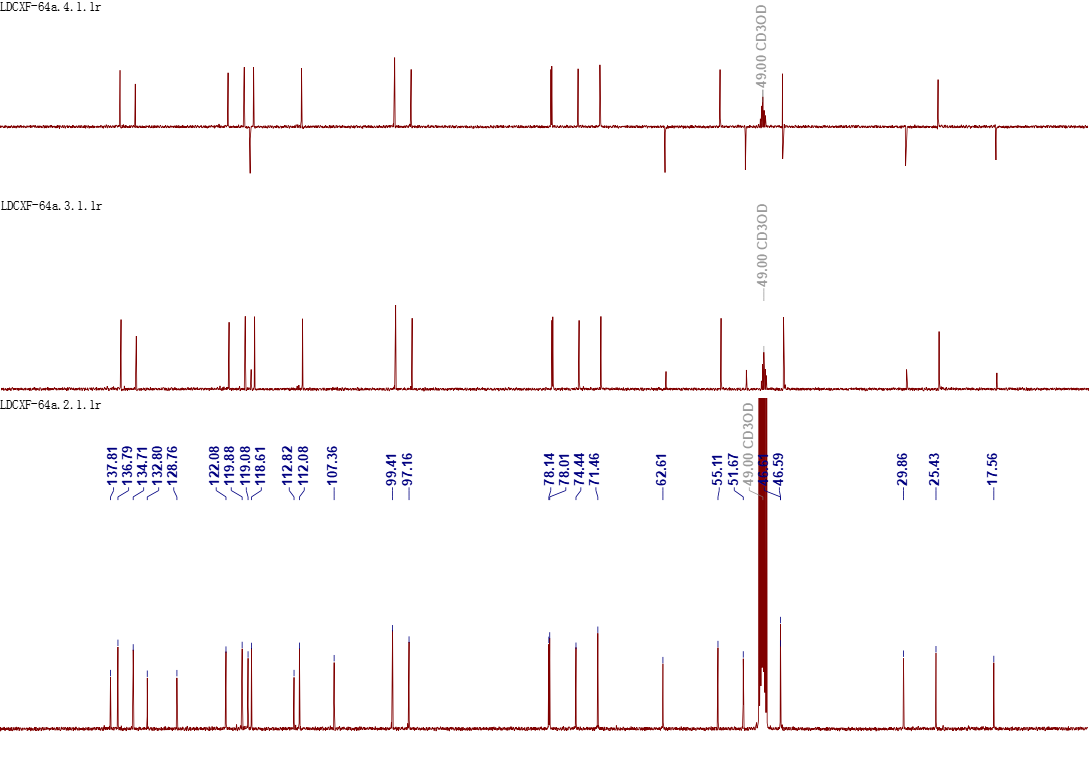


**Figure S22.2 ^13^C NMR spectrum of 22**


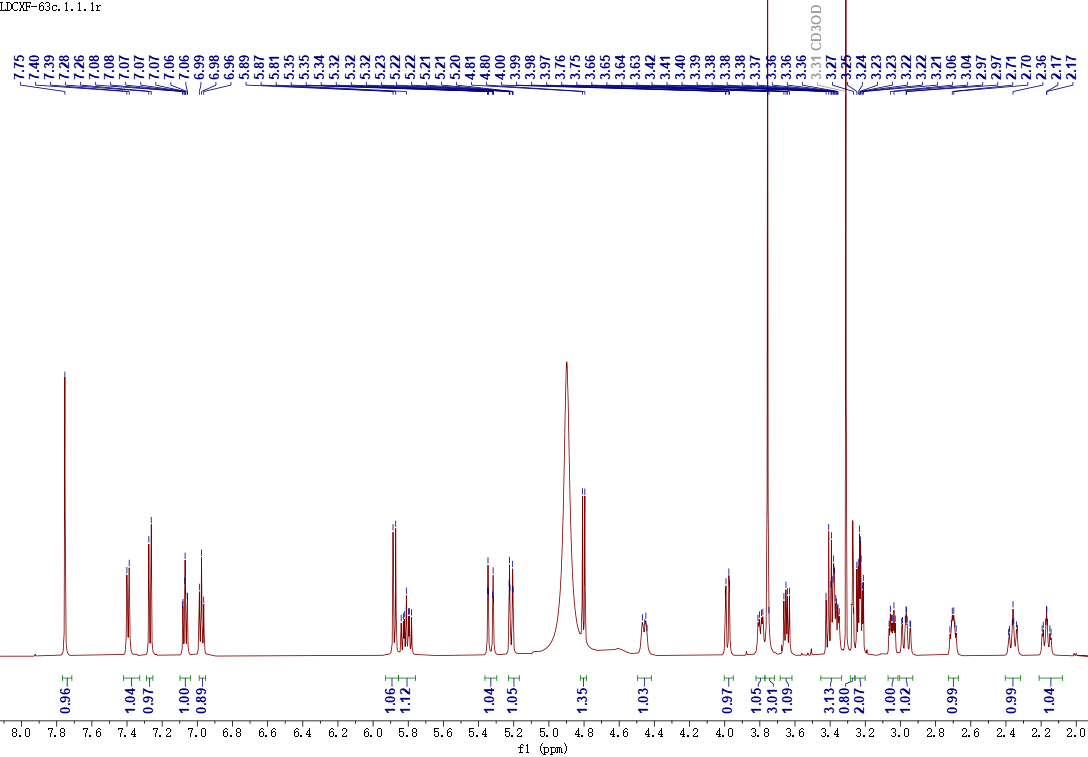


**Figure S23.1 ^1^H NMR spectrum of 23**


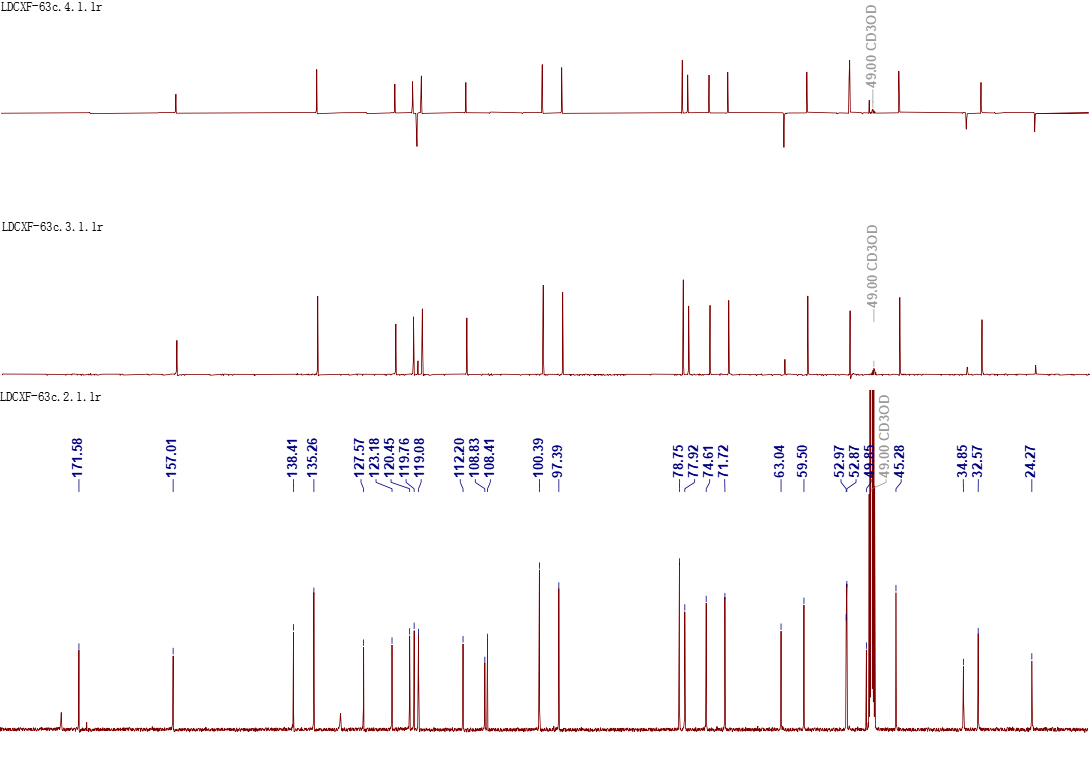


**Figure S23.2 ^13^C NMR spectrum of 23**

**Data attribution for known compounds**

**deppeaninol (11) *:*** C_19_H_22_N_2_O_2_，yellow crystals. ^1^H NMR (600 MHz, Methanol-*d*_4_) *δ*_H_ 8.16 (1H, d, *J* = 5.4 Hz, H-5), 8.12 (1H, dt, *J* = 7.9,1.0Hz, H-9), 7.89 (1H, d, *J* = 5.4 Hz, H-6), 7.52 (2H, m, *J* = 8.8 Hz, H-11 and H-12 ), 7.22 (1H, ddd, *J* = 8.0,6.7,1.2 Hz, H-10), 5.35 (1H, q, *J* = 6.9 Hz,H-19 ), 4.28 (1H, m, H-21),4.04 (1H, d, *J* = 12.3 Hz,H-21 ), 3.60 (1H, ddd, *J* = 10.7, 7.4, 5.6 Hz, H-17), 3.50 (1H, dt, *J* = 10.8, 7.1 Hz, H-15), 3.40 (1H, m, H-15), 3.33 (2H, m, H-14), 1.97 (1H, dddd, *J* = 12.9, 9.6, 7.0, 5.6 Hz, H-16), 1.92-1.83 (1H, m, H-16), 0.99 (3H, d, *J* = 6.8 Hz, H-CH_3_) . ^13^C NMR (150 MHz, Methanol-*d*_4_) δ_C_ 145.75 (C-13), 142.27 (C-3), 139.68 (C-2), 137.58 (C-5), 136.44 (C-20), 129.92 (C-7), 129.16 (C-11), 126.35 (C-19), 122.36 (C-9), 120.43 (C-8), 113.86 (C-6), 112.58 (C-12), 65.22 (C-21), 60.97 (C-17), 39.24 (C-14), 36.63 (C-16), 36.52 (C-15), 12.47 (C-18).

**(–)-dihydrocorynanthenol (12):** C_19_H_26_N_2_O, yellow powder. ^1^H NMR (600 MHz, Methanol-*d*_4_) *δ*_H_ 7.36 (1H, dt, J = 7.8, 1.0, Hz, H-9), 7.27 (1H, dt, *J* = 8.1, 1.0 Hz, H-12), 7.02 (1H, ddd, *J* = 8.1, 7.0, 1.2 Hz, H-11), 6.95 (1H, ddd, *J* = 8.0, 7.1, 1.0 Hz, H-10), 3.73 (1H, ddd, *J* = 10.7, 7.9, 4.9 Hz, H-3), 3.02 (4H, m, H-5, H-6), 0.94 (3H, t, *J* = 7.5 Hz, H-18). ^13^C NMR (150 MHz, Methanol-*d*_4_) *δ*_C_ 138.1 (C-13), 135.6 (C-2), 128.3 (C-8), 122.0 (C-11), 119.8 (C-10), 118.6 (C-9), 112.0 (C-12), 107.7 (C-7), 61.7 (C-3), 61.3 (C-21), 60.6 (C-17), 54.4 (C-5), 42.8 (C-20), 38.0 (C-15), 36.6 (C-14), 35.7 (C-16), 24.5 (C-19), 22.3 (C-6), 11.3 (C-18).

**desoxycordifoline (13):** C_28_H_30_N_2_O_11_, yellow powder. ^1^H NMR (600 MHz, Methanol-*d*_4_) *δ*_H_ 8.73 (1H, s, H-6), 8.21 (1H, d, *J* = 7.9 Hz, H-9), 7.61 (2H, d, H-12, H-17), 7.51 (1H, s, H-11),7.31 (1H, tt, *J* = 8.5, 5.4 Hz, H-10), 5.88 (1H, m, H-21), 5.84 (1H, m, H-19), 5.07 (1H,d, *J* =17.3 Hz, H-18b), 5.02 (1H,d, *J* =10.7 Hz, H-18a), 4.80 (1H, d, *J* = 7.9 Hz, H-1′), 3.97 (1H, d, *J* = 10.7 Hz, H-6′a), 3.69 (1H, m, H-6ʹb), 3.62 (1H, m, H-15), 3.55 (2H, ov, H-3', H-5′), 3.40 (1H, m, H-14a), 3.35 (3H, br-s, H-CH_3_), 3.27 (1H, m, H-14b), 3.24 (1H, m, H-4′), 3.22 (1H, m, H-2′),2.68 (1H, m, H-20). ^13^C NMR (151 MHz, Methanol-*d*_4_) *δ*_C_ 169.7 (C-22), 169.4 (C-COOH), 154.8 (C-17), 143.9 (C-3), 143.2 (C-13), 137.3 (C-2), 137.3 (C-5), 135.2 (C-19), 131.9 (C-7), 131.1 (C-11), 123.3 (C-10), 122.5 (C-8), 122.1 (C-9), 119.7 (C-18), 116.3 (C-6), 113.4 (C-12), 110.1 (C-16), 100.3 (C-1′), 97.3 (C-21), 78.6 (C-3′), 78.0 (C-5′), 74.7 (C-2′), 71.7 (C-4′), 62.9 (C-6′), 51.9 (C-24), 45.7 (C-20), 35.1 (C-15), 34.2 (C-14).

**3*α*-5*α*-tetrahydrodeoxycordifoline lactam (14):** C_27_H_31_N_2_O_11_, orange powder. ^1^H NMR (600 MHz, Methanol-*d*_4_) *δ*_H_ 7.42 (1H, d, *J* = 7.8 Hz, H-9), 7.31 (1H, d, *J* = 7.9 Hz, H-12), 7.26 (1H, d, *J* = 2.5 Hz, H-22), 7.07 (1H, ddd, *J* = 8.2, 7.0, 1.2 Hz, H-11)), 6.99 (1H, ddd, *J* = 8.0, 7.0, 1.0 Hz, H-10), 5.67 (1H, dt, *J* = 17.3, 10.0 Hz, C-19), 5.39 (1H, dd, *J* = 1.8 Hz, H-21), 5.36 (1H, dd, *J* = 1.9 Hz, H-18a), 5.31 (1H, dd, *J* = 10.2, 1.9 Hz, H-18b),4.96 (1H, m, H-3), 4.58 (1H, d, *J* = 7.9 Hz, H-1′), 4.17 (1H, dt, *J* = 7.1, 3.3 Hz, H-5), 3.86 (1H, dd, *J* = 11.8, 2.2 Hz, H-6′a), 3.64 (1H, dd, *J* = 11.9, 5.7 Hz, H-6′b), 3.28 (1H, t, *J* = 9.0 Hz, C-6a), 3.25 (1H, dd, *J* = 5.7, 2.2 Hz, H-3′), 3.21 (1H, m, C-4′), 3.00 (1H, dd, *J* = 9.1, 7.9 Hz, H-2′), 2.91 (1H, m, C-6′b), 2.86 (1H, td, *J* = 5.4, 2.3 Hz, H-15), 2.67 (1H, ddd, *J* = 9.9, 5.6, 1.8 Hz, C-20), 2.48 (1H, dt, *J* = 13.9, 5.2 Hz, C-14a), 2.19 (1H, ddd, *J* = 16.6, 12.3, 4.9 Hz, C-14b) . ^13^C NMR (150 MHz, Methanol-*d*_4_) *δ*_C_ 176.0 (C-23), 168.9 (C-17), 148.8 (C-22), 138.0 (C-13), 135.2 (C-2), 134.3 (C-19), 128.5 (C-8), 122.5 (C-11), 120.6 (C-18), 120.1 (C-10), 118.8 (C-9), 112.3 (C-12), 110.5 (C-7), 109.7 (C-16), 100.7 (C-1′), 98.3 (C-21), 78.2 (C-5′), 77.9 (C-3′), 74.3 (C-2′), 71.3 (C-4′), 62.5 (C-6′), 61.9 (C-5), 56.6 (C-3), 44.8 (C-20), 27.8 (C-14), 25.1 (C-15), 23.9 (C-6).

**strictosamide (15):** C_26_H_30_N_2_O_8_, yellow amorphous powder. ^1^H NMR (600 MHz, Methanol-*d*_4_) *δ*_H_ 7.36 (2H, m, H-12, H-17), 7.30 (1H, d, *J* = 8.1 Hz, H-9), 7.05 (1H, t, *J* = 7.6 Hz, H-11), 6.97 (1H, t, *J*= 7.4 Hz, H-10), 5.63 (1H, dt, J = 17.1, 10.1 Hz, H-19), 5.38 (1H, dd, *J* = 1.7 Hz, H-21), 5.34 (1H, dd, *J* = 17.1, 1.8 Hz, H-18a), 5.29 (1H, dd, *J* = 10.3, 1.9 Hz, H-18b), 5.06 (1H, m, H-3), 4.93 (1H, dd, *J* = 12.6, 5.4 Hz, H-5b), 4.55 (1H, d, *J* = 7.9 Hz, H-1′),3.83 (1H, dd, *J* = 11.8, 2.2 Hz, H-3ʹ),3.60 (1H, dd, *J* = 11.9, 6.0 Hz, H-4ʹ), 3.23 (2H, td, *J* = 9.3, 4.6 Hz, H-6ʹ), 3.15 ( 1H, t, *J* = 9.3 Hz, H-5ʹ) 3.09 (1H, dt, *J* = 12.5, 4.7 Hz, H-5a), 2.94 (2H, m, H-6, H-2′), 2.76 (1H, m, H-15), 2.68 (2H, m, H-6, H-20), 2.44 (1H, ddd, *J* = 14.1, 4.6, 2.0 Hz, H-14a), 2.04 (1H, dt, *J* = 13.7, 6.0 Hz, H-14b). ^13^C NMR (150 MHz, Methanol-*d*_4_) *δ*_C_ 167.1 (C-22), 149.2 (C-17), 137.8 (C-13), 134.8 (C-2), 134.4 (C-19), 128.7 (C-8), 122.5 (C-11), 120.6 (C-18), 120.2 (C-10), 118.7 (C-9), 112.2 (C-12), 110.3 (C-16), 109.2 (C-7), 100.5 (C-1′), 98.0 (C-21), 78.2 (C-3′), 77.9 (C-5′), 74.3 (C-2′), 71.3 (C-4′), 62.6 (C-6′), 55.1 (C-3), 44.8 (C-5), 44.7 (C-20), 27.3 (C-14), 24.9 (C-15), 22.1 (C-6).

**vincosamide (16):** C_26_H_30_N_2_O_8_, orange powder. ^1^H NMR (600 MHz, Methanol-*d*_4_) *δ*_H_ 5.37 (1H, dd, *J* = 17.2, 1.9 Hz, ), 5.32 (1H, dd, *J* = 10.0, 1.9 Hz,), 5.07 (1H, m, ), 4.94 (1H, dd, *J* = 12.9, 5.5 Hz, ), 4.57 (1H, d, *J* = 7.9 Hz, ), 3.85 (1H, dd, *J* = 11.8, 1.6 Hz, ), 3.62 (1H, dd, *J* = 11.9, 5.5 Hz, ), 3.28 – 3.16 (3H, m, , , ), 3.05 (1H, m, ), 2.96 (1H, m, ), 2.94 (1H, m, H-6b), 2.79 (1H, m, H-6a), 2.70 – 2.64 (2H, m, , H-15), 2.46 (1H, ddd, *J* = 14.2, 4.7, 2.1 Hz, H-14b), 2.04 (1H, dt, *J* = 13.9, 6.0 Hz, ). ^13^C NMR (150 MHz, Methanol-*d*_4_) *δ*_C_ 166.1 (C-22), 149.0 (C-17), 138.3 (C-13), 134.6 (C-2), 133.9 (C-19), 127.9 (C-8), 122.5 (C-11), 120.5 (C-18), 120.0 (C-10), 118.9 (C-9), 112.0 (C-12), 109.3 (C-7), 109.0 (C-16), 99.6 (C-1′), 97.4 (C-21), 78.3 (C-3′), 78.0 (C-5′), 74.8 (C-2′), 71.6 (C-4′), 62.7 (C-6′), 54.8 (C-3), 44.5 (C-5), 41.2 (C-20), 32.6 (C-14), 27.3 (C-15), 22.0 (C-6).

**desoxycordifoline (17):** C_28_H_30_N_2_O_11_, light yellow powder. ^1^H NMR (600 MHz, Methanol-*d*_4_) *δ*H 8.74 (1H, s, H-6), 8.22 (1H, s, H-9), 7.62 (2H, d, *J* = 4.3 Hz, H-11, H-12), 7.52 (1H, s, H-17) 7.31 (1H, m, H-10), 5.82 (2H, p, *J* = 9.5 Hz, H-19, H-21), 4.97 (1H, d, *J* = 10.5 Hz, H-18), 4.76 (1H, d, *J* = 7.9 Hz, H-1ʹ), 3.93 (1H, d, *J* = 11.9 Hz, H-6ʹ), 3.66 (1H, dd, *J* = 11.9, 6.6 Hz, H-6ʹ), 3.58 (1H, q, *J* = 6.8 Hz, H-15), 3.50 (2H, td, *J* = 13.1, 12.0, 7.2 Hz, H-14), 3.36 (2H, q, *J* = 10.4, 9.7 Hz, H-3ʹ, H-5ʹ), 3.31 (3H, s, H-CH_3_), 3.24 (1H, t, *J* = 9.4 Hz, H-4ʹ), 3.19 (1H, d, *J* = 8.5 Hz, H-2ʹ), 2.64 (1H, q, *J* = 7.1 Hz, H-20). ^13^C NMR (150 MHz, Methanol-*d*_4_) *δ*C 169.4 (C-22), 168.6 (C-23), 154.8 (C-17), 143.8 (C-13), 143.2 (C-3), 139.1 (C-5), 137.2 (C-2), 135.2, 131.8 (C-7), 131.0 (C-11), 123.3 (C-9), 122.6 (C-8), 122.1 (C-10), 119.7 (C-18), 116.2 (C-6), 113.4 (C-12), 110.2 (C-16), 100.3 (C-1ʹ), 97.3 (C-21ʹ), 78.6 (C-5ʹ), 78.0 (C-3ʹ), 74.6 (C-2ʹ), 71.7 (C-4), 62.9 (C-6), 51.9 (C-COOH), 45.6 (C-20), 35.2 (C-15), 34.3 (C-14).

**lyaloside (18):** C_27_H_30_N_2_O_9_, light yellow powder. ^1^H NMR (600 MHz, Methanol-*d*_4_) *δ* 8.18 (1H, d, *J* = 5.3 Hz, H-19), 8.12 (1H, d, *J* = 7.9 Hz, H-9), 7.91 (1H, d, *J* = 5.3 Hz, H-6), 7.58 (1H, d, *J* = 8.2 Hz, H-12), 7.53 (1H, m, H-11), 7.51 (1H, s, H-17), 7.24 (1H, t, *J* = 7.4 Hz, H-10), 5.84 (1H, dt, *J* = 18.1, 9.6 Hz, H-5), 5.73 (1H, d, *J* = 6.9 Hz, H-21), 3.91 (1H, d, *J* = 11.9 Hz, H-6ʹa), 3.69 (1H, dd, *J* = 12.1, 6.2 Hz, H-6ʹb), 3.56 (1H, q, *J* = 6.9 Hz, H-14a), 2.57 (1H, m, H-20). ^13^C NMR (150 MHz, Methanol-*d*_4_) *δ*C 169.6 (C-22), 154.2 (C-17), 144.8 (C-3), 142.3 (C-13), 137.8 (C-19), 136.5 (C-2), 135.2 (C-5), 130.0 (C-7), 129.5 (C-11), 122.6 (C-9), 122.3 (C-8), 120.8 (C-10), 119.6 (C-18), 114.3 (C-6), 112.9 (C-12), 110.9 (C-16), 100.1 (C-1ʹ), 97.5 (C-21), 78.3 (C-3ʹ), 77.7 (C-5ʹ), 74.5 (C-2ʹ), 71.4 (C-4ʹ), 62.6 (C-6ʹ), 51.9 (C-OCH_3_), 45.4 (C-20), 35.3 (C-14), 34.4 (C-15).

**Lyalosidie acid (19):** C_26_H_27_N_2_O_8_, pale yellow needles. ^1^H NMR (600 MHz, Methanol-*d*_4_) *δ*_H_ 8.10 (1H, d, *J* = 5.8 Hz, H-9), 8.05 (1H, d, *J* = 8.0 Hz, H-5), 7.96 (1H, d, *J* = 5.8 Hz, H-6), 7.60 (1H, d, *J* = 8.3 Hz, H-12), 7.51 (1H, ddd, *J* = 8.2, 7.0, 1.1 Hz, H-11), 7.43 (1H, s, H-17), 7.19 (1H, t, *J* = 7.5 Hz, H-10), 5.80 (1H, dt, *J* = 18.3, 9.3 Hz, H-19), 5.61 (1H, d, *J* = 7.3 Hz, H-21),4.77 (1H, s, H-1′), 4.74 (2H, m, H-18), 3.91 (1H, dd, *J* = 12.3, 2.2 Hz, H-6′a), 3.68 (1H, dd, *J* = 12.1, 6.4 Hz, H-6′b), 3.53 (1H, m, H-14), 3.45 (1H, dt, *J* = 9.1, 4.5 Hz, H-14), 3.35 (1H,ddd, *J* = 8.7, 6.3, 2.2 Hz, H-4′), 3.27 (1H, m, H-3′), 3.27 (1H, m, H-5′), 3.20 (1H, m, H-15), 3.20 (1H, m, H-2′) 2.52 (1H, td, *J* = 8.2, 5.6 Hz, H-20).^13^C NMR (150 MHz, Methanol-*d*_4_) *δ*_C_ 173.9 (C-COOH), 151.7 (C-17), 144.5 (C-3), 143.2 (C-2), 136.0 (C-5), 135.8 (C-13), 134.1 (C-19), 131.3 (C-7), 130.6 (C-11), 123.0 (C-9), 121.9 (C-8), 121.3 (C-10), 119.0 (C-18), 115.0 (C-6), 114.4 (C-16), 113.4 (C-12), 100.2 (C-1′), 97.0 (C-21), 78.4 (C-3′), 77.9 (C-5′), 74.6 (C-2′), 71.6 (C-4′), 62.9 (C-6′), 46.1(C-20), 35.9 (C-14), 35.9 (C-15).

**strictosidinic acid (20):** C_26_H_32_N_2_O_9_, light yellow powder. ^1^H NMR (600 MHz, Methanol-*d*_4_) *δ*H 7.54 (1H, s, H-17), 7.40 (1H, d, *J* = 7.9 Hz, H-9), 7.31 (1H, d, *J* = 8.2 Hz, H-12), 7.11 (1H, t, *J* = 7.6 Hz, H-11), 7.01 (1H, t, *J* = 7.5 Hz, H-10), 5.84 (1H, m, H-19), 5.80 (1H, d, *J* = 9.4 Hz, H-21), 5.28 (1H, d, *J* = 17.3 Hz, H-18a), 5.18 (1H, d, *J* = 10.7 Hz, H-18b), 4.84 (1H, d, *J* = 7.9 Hz, H-1ʹ), 4.29 (1H, m, H-3), 4.02 (1H, m, H-6ʹa), 3.69 (1H, dd, *J* = 11.9, 6.7 Hz, H-6ʹb), 3.46 (1H, t, *J* = 9.1 Hz, H-3ʹ), 3.41 (1H, ddd, *J* = 9.1, 6.7, 2.2 Hz, H-5ʹ), 3.27 (2H, dt, J = 17.0, 9.0 Hz, H-2ʹ, H-4ʹ), 2.98 (1H, dt, *J* = 12.5, 4.7 Hz, H-15), 2.83(1H, m, H-14a), 2.65 (1H, td, *J* = 8.4, 4.7 Hz, H-20), 2.07 (1H, ddd, *J* = 14.1, 11.8, 4.4 Hz, H-14b). ^13^C NMR (150 MHz, Methanol-*d*_4_) *δ*C 176.1 (C-22), 153.0 (C-17), 138.0 (C-13), 136.3 (C-19), 131.1 (C-2), 127.6 (C-8), 123.2 (C-11), 120.4 (C-10), 119.0 (C-9), 118.9 (C-18), 113.9 (C-16), 112.2 (C-12), 107.3 (C-7), 100.3 (C-1ʹ), 96.6 (C-21), 78.6 (C-5ʹ), 77.9 (C-3ʹ), 74.7 (C-2ʹ), 71.7 (C-4ʹ), 63.0 (C-6ʹ), 52.2 (C-3), 45.6 (C-20), 43.0 (C-5), 35.2 (C-14), 33.9 (C-15), 19.7 (C-6).

**5*α*-carboxystrictosidine** **(21):** C_28_H_34_N_2_O_11_, light yellow powder. ^1^H NMR (600 MHz, Methanol-*d*_4_) *δ*H 7.75 (1H, s, H-17), 7.43 (1H, d, *J* = 7.9 Hz, H-9), 7.26 (1H, d, *J* = 8.1 Hz, H-12), 7.07 (1H, t, *J* = 7.6 Hz, H-11), 6.99 (1H, t, *J* = 7.5 Hz, H-10), 5.88 (1H, d, *J* = 9.1 Hz, H-21), 5.83 (1H, ddd, *J* = 17.9, 10.7, 7.6 Hz, H-18), 5.34 (1H, dt, *J* = 17.4, 1.5 Hz, H-19a), 5.22 (1H, dt, *J* = 10.6, 1.3 Hz, H-19b), 4.79 (1H, d, *J* = 7.9 Hz, H-1′), 4.43 (1H, m, H-3), 3.98 (1H, dd, *J* = 11.8, 2.1 Hz, H-6′a), 3.76 (1H,s, H-OCH_3_),3.76 (1H,s, H-5), 3.65 (1H, dd, *J* = 11.9, 7.0 Hz, H-6′b), 3.41 (1H, m, H-3′),3.38 (1H, m, H-5′), 3.35 (1H, m, H-6a), 3.25 (1H, m, H-4′),3.21 (1H, m, H-2′), 3.09 (1H, dt, *J* = 12.2, 4.2 Hz, H-15), 2.93 (1H, t, *J* = 14.0 Hz, H-6b), 2.73 (1H, q, *J* = 8.0, 7.5 Hz, H-20), 2.33 (1H, t, *J* = 13.3 Hz, H-14a), 2.13 (1H, t, *J* = 13.0 Hz, H-14b). ^13^C NMR (150 MHz, Methanol-*d*_4_) *δ*C 171.3 (C-23), 170.3 (C-22), 156.6 (C-17), 138.4 (C-13), 135.5 (C-18), 127.9 (C-8), 122.9 (C-11), 120.3 (C-10), 119.6 (C-19), 119.0 (C-9), 112.1 (C-12), 108.8 (C-16), 108.7 (C-7), 100.4 (C-1′), 97.4 (C-21), 78.8 (C-5′), 78.0 (C-3′), 74.7 (C-2′), 71.8 (C-4′), 63.1 (C-6′), 59.8 (C-5), 52.9 (C-3), 52.7 (C-OCH_3_), 45.5 (C-20),35.4 (C-14), 32.7 (C-15), 24.8 (C-6).

**deoxystrictosamide *(*22*):*** C_26_H_32_N_2_O_7_, yellow amorphous powder. ^1^H NMR (600 MHz, Methanol-*d*_4_) *δ*_H_ 7.38 (1H, d, *J* = 7.8 Hz, H-9), 7.32 (1H, d, *J* = 8.0 Hz, H-12), 7.06 (1H, ddd, *J* = 8.2, 7.0, 1.2 Hz, H-11), 6.98 (1H, ddd, *J* = 8.1, 7.0, 1.0 Hz, H-10), 6.20 (1H, s, H-17), 5.79 (1H, dt, *J* = 17.2, 10.2 Hz, H-19), 5.29 (1H, d, *J* = 2.1 Hz, H-21), 5.25 (1H, d, *J* = 2.1 Hz, H-18a), 5.24 (1H, m, H-18b), 4.52 (1H, d, *J* = 8.0 Hz, H-1′), 4.47 (1H, dd, *J* = 5.4, 2.5 Hz, H-3), 3.85 (1H, dd, *J* = 11.8, 1.9 Hz, H-6ʹa), 3.63 (1H, dd, *J* = 11.8, 5.3 Hz, H-6ʹb), 3.44 (1H, dt, *J* = 13.2, 2.1 Hz, H-22a),3.28 (1H, m, H-3ʹ), 3.25 (1H, m, H-5ʹ), 3.24 (1H, m, H-4ʹ) 3.01 (1H,m,H-2ʹ), 2.98 (1H, m, H-22b), 2.63 (1H, m, H-20), 2.63 (1H, m, H-6b), 2.46 (1H, m, H-15), 2.30 (1H, m, H-14a), 1.90 (1H, m, H-14b). ^13^C NMR (150 MHz, Methanol-*d*_4_) *δ*_C_ 137.8 (C-13), 136.8 (C-19), 134.7 (C-17), 132.8 (C-2), 128.8 (C-8), 122.1 (C-11), 119.9 (C-10), 119.1 (C-18), 118.6 (C-9), 112.8 (C-16), 112.1 (C-12), 107.4 (C-7), 99.4 (C-1′), 97.2 (C-21), 78.1 (C-3′), 78.0 (C-5′), 74.4 (C-2′), 71.5 (C-4′), 62.6 (C-6′), 55.1 (C-3), 51.7 (C-5), 46.6 (C-20), 46.6 (C-22), 29.9 (C-14), 25.4 (C-15), 17.6 (C-6).

**turbinatine (23):** C_27_H_34_N_2_O_9_, pale yellow needles. ^1^H NMR (600 MHz, Methanol-*d*_4_) *δ*_H_ 7.75 (1H, s, H-17), 7.39 (1H, d, *J* = 7.9 Hz, H-9), 7.27 (1H, d, *J* = 8.2 Hz, H-12), 7.07 (1H, ddd, *J* = 8.2, 7.0, 1.1 Hz, H-11), 6.98 (1H, t, *J* = 7.5 Hz, H-10), 5.88 (1H, d, *J* = 9.2 Hz, H-21), 5.81 (1H, ddd, *J* = 17.8, 10.7, 7.4 Hz, H-19), 5.33 (1H, dt, *J* = 17.4, 1.4 Hz, H-18a), 5.21 (1H, dt, *J* = 10.7, 1.3 Hz, H-18b), 4.80 (1H, d, *J* = 8.0 Hz, H-1′), 4.45 (1H, dd, *J* = 9.9, 6.0 Hz, H-3), 3.98 (1H, dd, *J* = 11.9, 2.1 Hz, H-6′a), 3.80 (1H, dd, *J* = 12.0, 4.9 Hz, H-5a), 3.76 (3H, s, H-OCH_3_), 3.65 (1H, dd, *J* = 11.9, 7.0 Hz, H-4′), 3.40 (1H, m, H-6b′), 3.40 (1H, m, H-5b), 3.40 (1H, m, H-3′), 3.27 (1H, m, H-5′), 3.24 (1H, m, H-2′), 3.23 (1H, m, H-6a), 3.05 (1H, dt, *J* = 12.4, 4.3 Hz, H-15), 2.97 (1H, m, H-6b), 2.70 (1H, td, *J* = 8.0, 4.5 Hz, H-20), 2.36 (1H, ddd, *J* = 14.8, 12.2, 3.0 Hz, H-14a), 2.17 (1H, m, H-14b). ^13^C NMR (150 MHz, Methanol-*d*_4_) *δ*_C_ 171.6 (C-22), 157.0 (C-17), 138.4 (C-2), 138.4 (C-13), 135.3 (C-19), 127.6 (C-8), 123.2 (C-11), 120.5 (C-10), 119.8 (C-18), 119.1 (C-9), 112.2 (C-12), 108.8 (C-9), 108.4 (C-16), 100.4 (C-1′), 97.4 (C-21), 78.7 (C-5′), 77.9 (C-3′), 74.6 (C-2′), 71.7 (C-4′), 63.0(C-6′), 53.0 (C-3), 52.9 (C-OCH_3_), 49.8 (C-5), 45.3 (C-20), 34.9 (C-14), 32.6 (C-15), 24.3 (C-6).

**Computational details**

**S1 Computational details for compound 1**

**Table S1.** Energy analysis for conformers of **1-1~1-8** at M062X/def2svp level in the gas phase (T=298.15 K)

| Conformers | ***E*** | ***C*** | ***G*** | ***ΔG (kcal/mol)*** | ***PE%*** |
| --- | --- | --- | --- | --- | --- |
| 1-1 | -1333.36203 | 0.317516 | -1333.044514 | 0.000000 | 38.55% |
| 1-2 | -1333.362447 | 0.317962 | -1333.044486 | 0.017570 | 37.42% |
| 1-3 | -1333.363314 | 0.31906 | -1333.044254 | 0.163153 | 29.27% |
| 1-4 | -1333.361518 | 0.317502 | -1333.044016 | 0.312500 | 22.74% |
| 1-5 | -1333.36289 | 0.318878 | -1333.044012 | 0.315010 | 22.65% |
| 1-6 | -1333.362334 | 0.318522 | -1333.043813 | 0.439884 | 18.34% |
| 1-7 | -1333.361036 | 0.317725 | -1333.043311 | 0.754894 | 10.77% |
| 1-8 | -1333.361346 | 0.318135 | -1333.043211 | 0.817645 | 9.69% |

***E:***Electronic energy, ***C:*** Thermal correction to Gibbs free energy, ***G:*** Gibbs free energy(E+C),***ΔG:*** The relative Gibbs free energy;PE%:The Boltzmann distribution of each conformer.


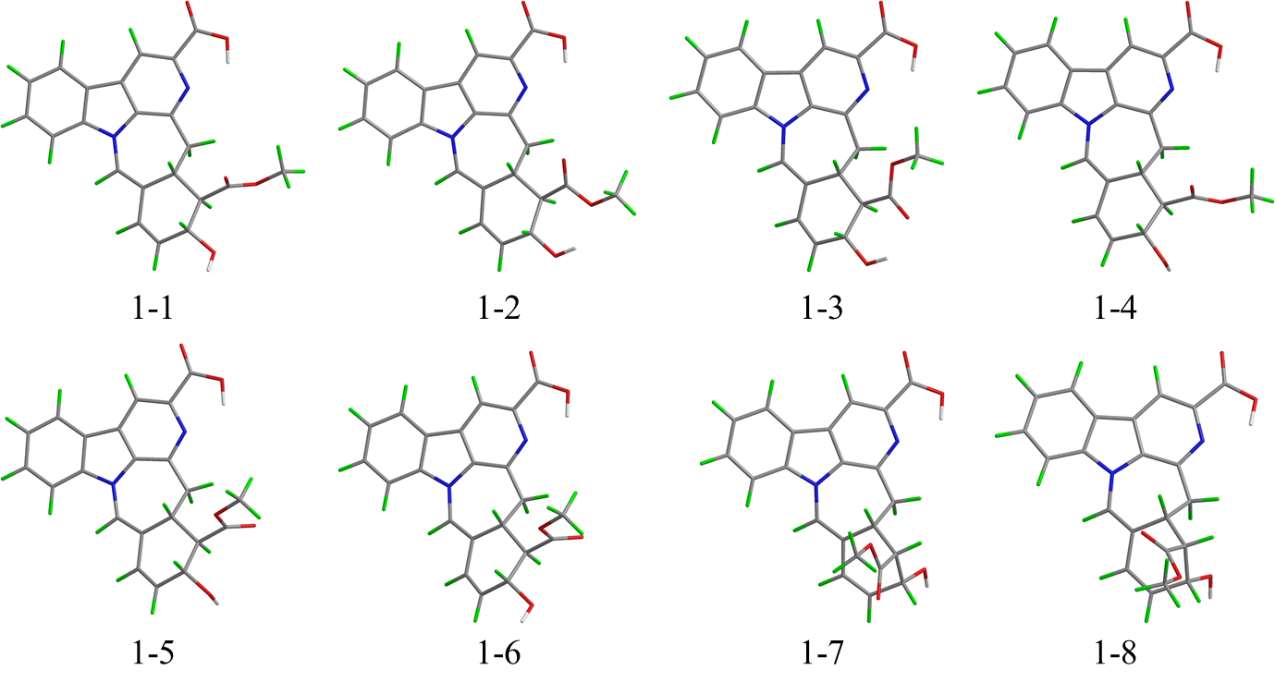


**Figure S1.** Optimized conformations of **1** (the relative populations are in parentheses)


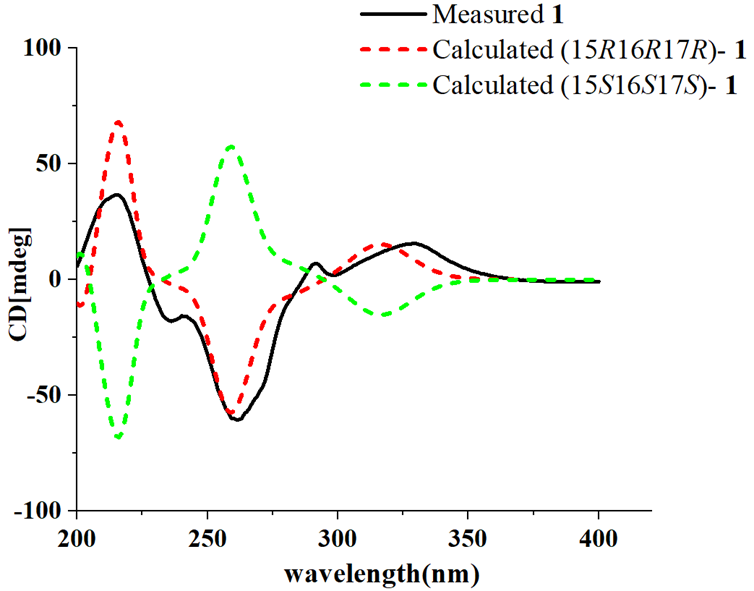


**Figure S2.** Calculated ECD spectra for **1** at the wB97xd/def2svp level in methanol with IEFPCM model (σ = 0.35 eV). Experimental CD spectra of **1** (black line) in MeOH.

**S2 Computational details for compound 2**

**Table S2.** Energy analysis for conformers of **2-1~2-2** at M062X/def2svp level in the gas phase (T=298.15 K)

| Conformers | ***E*** | ***C*** | ***G*** | ***ΔG (kcal/mol)*** | ***PE%*** |
| --- | --- | --- | --- | --- | --- |
| 2-1 | -1313.6270631 | 0.331823 | -1313.29524 | 0.000000 | 69.32% |
| 2-2 | -1313.6268636 | 0.332393 | -1313.294471 | 0.482547 | 30.68% |

***E:***Electronic energy, ***C:*** Thermal correction to Gibbs free energy, ***G:*** Gibbs free energy(E+C),***ΔG:*** The relative Gibbs free energy;***PE%***:The Boltzmann distribution of each conformer.


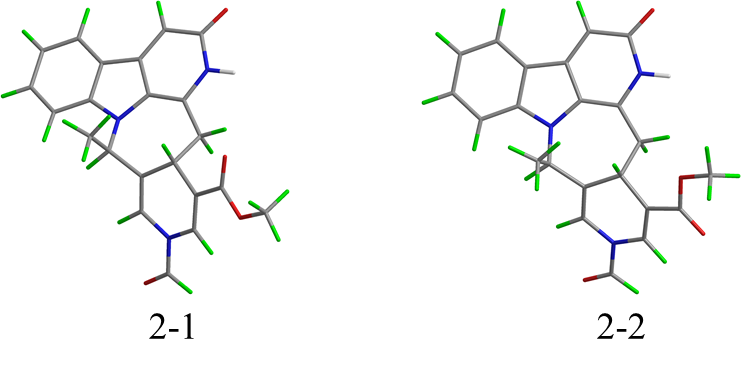


**Figure S3.** Optimized conformations of **2** (the relative populations are in parentheses)


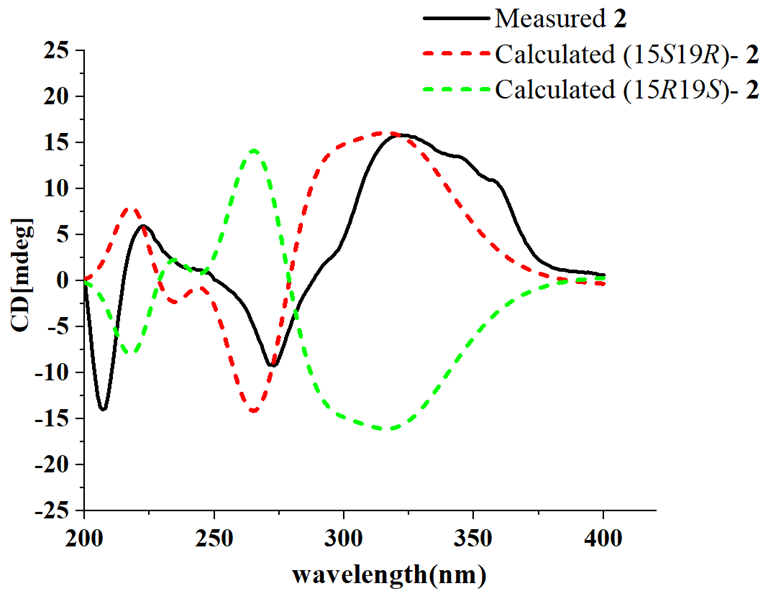


**Figure S4.** Calculated ECD spectra for **2** at the wB97xd/def2svp level in methanol with IEFPCM model (σ = 0.35 eV). Experimental CD spectra of **2** (black line) in MeOH.
